# Interventions to enhance in-home taking medication among older adults with multimorbidity/polypharmacy: a systematic review and meta-analysis

**DOI:** 10.3389/fpubh.2025.1701622

**Published:** 2026-01-28

**Authors:** María Durán-Luque, María Rocío Robles-Muñoz, María Eugenia Velasco-García, Celia Gómez-Peña, Ángel Cobos-Vargas, Maria Núñez-Núñez, Aurora Bueno-Cavanillas

**Affiliations:** 1 Department of Preventive Medicine and Public Health, University of Granada, Granada, Spain; 2 Hospital Universitario San Cecilio Unidad de Gestion Clinica de Farmacia Hospitalaria, Granada, Spain; 3 Department of Statistics and Operations Research, Universidad de Granada, Granada, Spain; 4 Instituto de Investigacion Biosanitaria de Granada, Granada, Spain; 5 Intensive Care Unit, Hospital Universitario San Cecilio, Granada, Spain; 6 Centro de Investigacion Biomedica en Red de Epidemiologia y Salud Publica, Madrid, Spain

**Keywords:** aged, drug therapy, frail older adults, home environment, medication therapy management, meta-analysis, multimorbidity, polypharmacy

## Abstract

**Introduction:**

Global population ageing is linked to increasing multimorbidity and polypharmacy. This shift places pressure on caregivers, who often lack training and face challenges like medication mismanagement. Our objective was to collate scientific evidence on interventions to enhance medication management among multimorbid older adults living in the community.

**Methods:**

We conducted a systematic review and meta-analysis (PROSPERO: CRD42024513056) following PRISMA guidelines. PubMed, Web of Science, CINAHL, and ClinicalTrials.gov were searched up to July 9, 2024. Eligible studies were RCTs or quasi-experimental designs involving home-dwelling adults aged ≥60 years with ≥2 chronic conditions or ≥5 medications, assessing adherence or health outcomes, and ≥30 days of follow-up. Screening, data extraction, and quality assessment were performed in duplicate. Random-effects meta-analyses were conducted using R. ORs (95% CI) were calculated for binary outcomes and SMDs (95% CI) for continuous variables.

**Results:**

Of 7,980 citations, 49 articles met the eligibility criteria, corresponding to 48 unique studies. Medication adherence measured with the MMAS-4 indicated a significant effect (OR = 1.55; 95% CI 1.08–2.28; I^2^ = 32.4%), while continuous measures showed no effect (SMD = 0.00; 95% CI = -0.08–0.09; I^2^ = 2%). Readmissions decreased at medium-term follow-up (OR = 0.41; 95% CI 0.25–0.69). Results for ED visits were inconclusive due to heterogeneity. Primary care contacts showed a weak, non-significant effect (SMD = 0.06; 95% CI = -0.04–0.16; I^2^ = 42%). No effect was found for quality of life or mortality. DRPs and costs lacked conclusive evidence. Most studies had a moderate to high risk of bias. Certainty of evidence was very low.

**Discussion:**

Interventions showed limited and variable effects. Adherence improvements were identified only for the MMAS-4, while other measures showed no benefit. A short-term reduction in readmissions was observed, but effects were not sustained, and the certainty of evidence was low. This review highlights evidence gaps, particularly the need for standardized outcomes, more sustained and multifactorial interventions, economic evaluation, and higher methodological quality, to support evidence-based policymaking and optimize future interventions.

**Systematic review registration:**

The systematic review was registered in PROSPERO (CRD42024513056). The registration link is: https://www.crd.york.ac.uk/PROSPERO/view/CRD42024513056.

## Introduction

Population ageing presents a significant health challenge in the 21st century (1, 2). The United Nations projects a doubling of older adults (≥65 years) by 2050, comprising 16% of the global population (3). This demographic shift will lead to an increase in chronic diseases, multimorbidity, and polypharmacy (4). Multimorbidity, defined as the coexistence of ≥2 chronic conditions, affects over 50% of older adults worldwide, representing a public health concern (5, 6) due to its association with reduced quality of life, high mortality, functional decline, and increased healthcare utilization (7, 8). These issues are compounded by polypharmacy, defined as the concurrent use of ≥5 medications (9), which affects 32.1% of Europeans aged ≥65 years (10) and increases the risk of adverse drug events, medication non-adherence, and ineffective treatment regimens (11, 12).

Healthcare systems, designed primarily for acute illnesses, struggle to meet the complex needs of patients with multimorbidity (13, 14). Consequently, 80–90% of care required by older adults is provided at home by informal caregivers (15), often family members, predominantly women (15–17). Shifting family structures and increased female participation in the workforce have strained families’ care capacity (15, 18–20), leading to reliance on migrant women, particularly in high-income countries (15, 20). These caregivers often lack formal training or adequate support, heightening risks of medication errors and adverse health outcomes (21).

Medication safety is a global priority (22), and strategies to improve adherence have involved various healthcare professionals. However, most studies have focused on isolated aspects of adherence, and robust evidence remains limited, particularly for home-dwelling older adults (23–25). The Cochrane systematic review by Cross et al. highlighted this gap, reporting insufficient evidence on the effectiveness of interventions in this population, primarily due to limited trial quality (26). This review aims to collate updated evidence on interventions to improve in-home medication management among multimorbid older adults living in the community (independently or with caregivers), and to assess the quality and the strength of the resulting recommendations.

## Materials and methods

This systematic review followed the Preferred Reporting Items for Systematic Review and Meta-Analysis (PRISMA) guidelines (27) (Supplementary Table S1) and was prospectively registered in PROSPERO with the registration number CRD42024513056.

### Search methods

A preliminary search was conducted in November and December 2023 using PubMed and Web of Science (WOS). The final search was updated on 9th July 2024 and expanded to include CINAHL to capture nursing and allied health literature relevant to medication management in older adults. For PUBMED and WOS, the following strategy was applied: (ELDERLY OR OLDER ADULTS OR DWELLING ADULTS OR CARER OR CAREGIVER) AND (POLYPHARMACY OR MULTIPLE MEDICATIONS) AND (MEDICATION MANAGEMENT OR MEDICATION ADHERENCE OR SAFE* MEDICATION) AND (HOME CARE OR TRANSITIONAL CARE OR PHARMACEUTICAL CARE) NOT CHILD*.

The CINAHL search strategy, adapted to the database’s specific indexing terms and syntax, is provided in the Supplementary Table S2.

References from included studies and prior systematic reviews were manually searched. ClinicalTrials.gov was searched using the keywords “home-dwelling,” “older adults,” “polypharmacy,” and “intervention,” without additional results. Studies published from 2000 onward were included without language restrictions.

### Inclusion and exclusion criteria

#### Study design

Eligible studies included Randomized Controlled Trials (RCTs), cluster RCTs, quasi-experimental studies, interrupted time series (ITS) analyses, and pre−/post-studies. Pilot RCTs, observational studies, or qualitative research, and those with <30 days follow-up were excluded.

#### Population

The review focused on older adults at high risk of Drug-Related Problems (DRPs), living independently in the community or with their caregivers, including those recently discharged from hospitals or nursing facilities to home-based care. Participants had a mean age of ≥ 60 years, with ≥ 2 chronic conditions or using ≥5 long-term medications. Those institutionalized or whose medication was managed by home nurses were excluded.

Community or transitional care settings were included; residential or nursing homes, or hospitals without post-discharge follow-up were excluded.

#### Intervention

Interventions aimed to enhance medication adherence or health outcomes; palliative care was excluded.

#### Comparator

Standard care practices were used as the comparator.

#### Outcomes

Studies that measured at least one of the following main outcomes were considered for inclusion:

- Adherence, measured by indirect methods, validated questionnaires (such as the Morisky Scale), pill count, electronic databases, Medication Events Monitoring System (MEMS), and medication ratios such as the Proportion of Days Covered (PDC) and Medication Possession Ratio (MPR). Other self-report adherence scales and indirect measures were also included.
- Adverse health outcomes: hospital (re)admissions, emergency department (ED) visits, length of stay, drug-related problems (DRPs), adverse drug events (ADEs), and drug withdrawal events (ADWES) were considered. Although not in the PROSPERO protocol, healthcare encounters (e.g., outpatient visits, specialist consultations, and primary care contacts) were also included as exploratory outcomes because they may indicate health complications, medication management challenges, or overuse of healthcare services.

Health-related quality of life (measured using the EuroQol-5D), as well as elders’ knowledge, attitudes, and behaviors related to medication; impact on functioning, cognition, urinary incontinence, falls, fractures, sleep quality, appetite, mortality, and costs related to pharmaceuticals and healthcare were considered as secondary outcomes. Studies reporting only secondary outcomes were excluded.

### Study selection

Two authors (M.D-L., M.R.R-M.) independently screened studies by title/abstract using the Rayyan web tool for systematic reviews, applying eligibility criteria. Relevant full-text articles were retrieved and assessed for eligibility, with the authors blinded to each other’s decisions. Disagreements were resolved through discussion, and a third author (C.G-P, A.B-C.) was consulted if necessary. Excluded studies were listed in an Excel spreadsheet with reasons (Supplementary Table S3). Selected studies were imported into Mendeley Reference Manager.

### Data extraction

Data extraction was performed independently by two review authors (M.D-L., M.R.R-M.). Discrepancies were resolved through discussion or consultation with a third author (C.G-P., A.B-C.) if necessary. All data were tabulated using a pre-designed Excel spreadsheet.

### Quality assessment

Risk of bias was independently evaluated by two authors (M.D-L., M.R.R-M.). We used the updated Cochrane Risk of Bias 2 (ROB 2) (28) for randomized studies and the Risk Of Bias In Non-randomized Studies of Interventions (ROBINS-I) (29) tool for non-randomized studies. To visually summarize the risk of bias, the robvis tool was employed. Studies were rated as high, low, or some concerns.

### Quantitative analysis

Meta-analysis was conducted using a random-effects model due to anticipated heterogeneity across studies. The analysis was performed in R software version 4.4.1, specifically using the metafor package version 8.0.1. Effect sizes were expressed as standardized mean differences (SMD) for continuous outcomes, and odds ratios (ORs) for dichotomous outcomes. Pooled estimates were calculated assigning study weights based on the inverse of the variance of the study effect size, which includes the intra- and inter-study variability.

Continuous adherence outcomes were measured using several instruments with different scales and score ranges (e.g., MMAS-4, MMAS-8, and other self-reported questionnaires); therefore, the standardized mean difference (SMD) was chosen over raw mean differences to allow the results to be expressed on a common, unitless scale, enabling comparison and pooling across studies. It was calculated by dividing the mean difference between the intervention and control groups by the standard deviation of post-intervention values.

Between-study variance (τ^2^) was estimated using the DerSimonian–Laird (DL) method, a widely used approach in random-effects meta-analyses. Where heterogeneity was moderate or high (I^2^ > 50%) and sufficient studies were available (≥5 per outcome), subgroup analyses (e.g., by follow-up duration or intervention setting) were conducted to explore potential sources of variability. Meta-regression was not performed due to the limited number of studies within each outcome. For outcomes with low or non-significant heterogeneity, further exploration was not undertaken.

Funnel plots were generated for outcomes including ≥10 studies and were visually examined for asymmetry to explore potential small-study effects, as recommended in the Cochrane Handbook for Systematic Reviews of Interventions (30). Peters’ test was applied for dichotomous outcomes, with *p* < 0.10 indicating potential asymmetry. For outcomes with <10 studies, neither funnel plots nor formal tests were conducted due to low power.

### Certainty of evidence

The certainty of evidence was assessed using the GRADE (Grading of Recommendations Assessment, Development and Evaluation) approach (31). GRADE was applied to outcomes considered clinically important or critical and sufficiently homogeneous to allow pooled quantitative estimation. The results were presented in a Summary of Findings table generated in GRADEpro.

## Results

**Figure 1:**
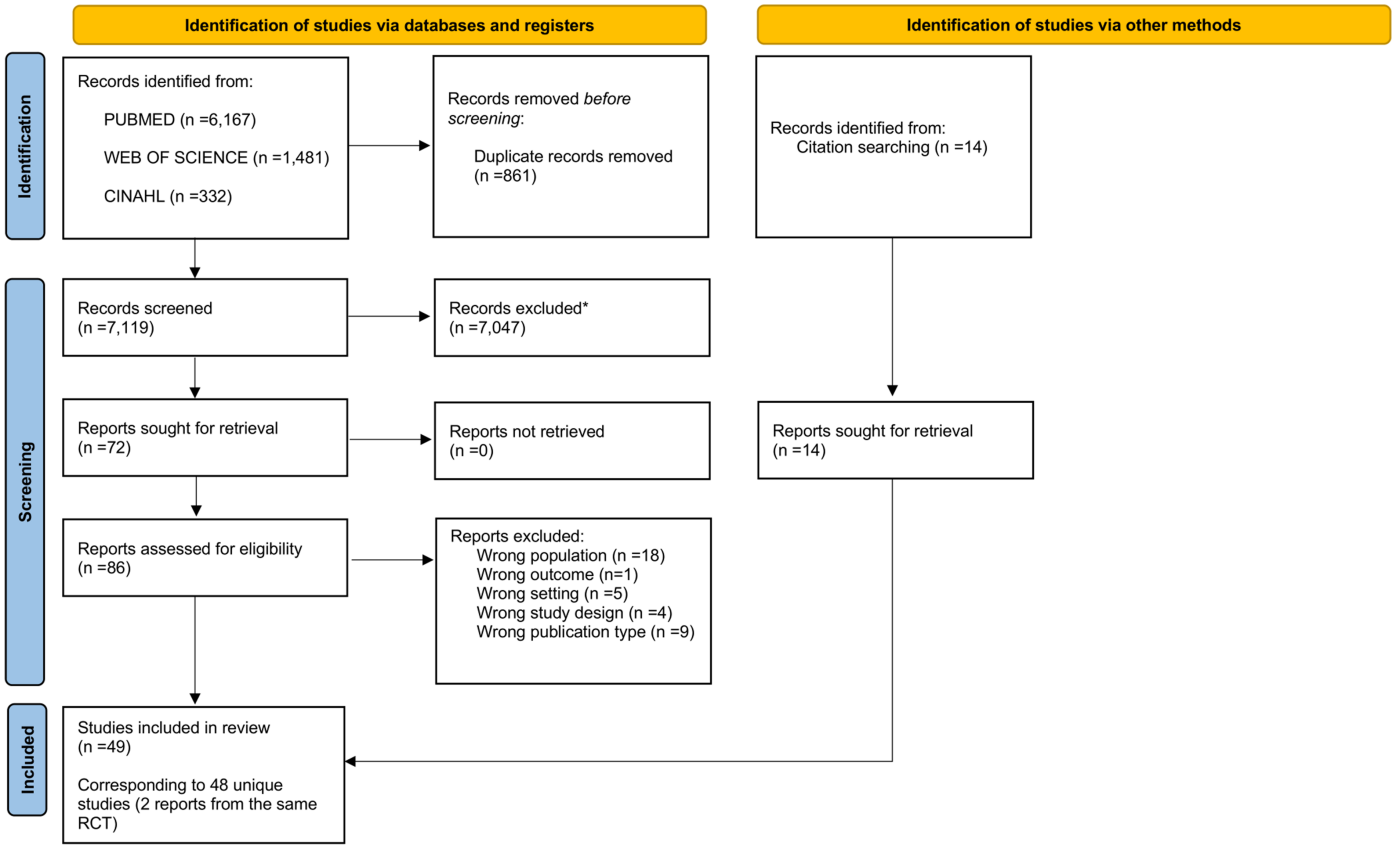
PRISMA flow diagram of study selection. Flow of records through identification, screening, eligibility, and inclusion phases according to PRISMA 2020 guidelines.

A total of 7,980 records were identified through database searches, and 14 through reverse searching. After removing duplicates and screening by title and abstract, 86 reports were assessed for eligibility. Following full-text review, 49 articles met the eligibility criteria, corresponding to 48 unique studies, as two independent publications reported different outcomes from the same RCT (32, 33). The screening process is summarized in Figure 1.

### Studies

Characteristics of the included studies, 37 RCTs and 11 quasi-experimental, are summarized in Table 1 and detailed in Supplementary Tables S4a,b. Published between 2001 (34, 35) and 2023 (36–38), most were conducted in the United States (11/48, 22.9%), the United Kingdom (6/48, 12.5%), and Spain (5/48, 10.4%). One involved several countries (34).

**Table 1:** Overview of the characteristics of the included studies.

Variable	Categories	Studies, *n* (%)	Variable	Categories	Studies, *n* (%)
Characteristics of the included studies					
Country	USA	11 (22.9%)	Setting	Community	**37 (77.1%)**
	United Kingdom	6 (12.5%)		Primary Care	19 (39.6%)
	Spain	5 (10.4%)		Community pharmacies	4 (8.3%)
	Netherlands	5 (10.4%)		Home-based	7 (14.6%)
	Ireland	3 (6.2%)		Home-Community dispensary	1 (2.1%)
	China	2 (4.2%)		Secondary Care	6 (12.5%)
	Denmark	2 (4.2%)		Transitional care	**11 (22.9%)**
	Germany	2 (4.2%)		Hospital Discharge	9 (18.7%)
	Multicountry^1^	1 (2.1%)		SNF Discharge	1 (2.1%)
	Other Countries^2^	11 (22.9%)		Emergency Department	1 (2.1%)
Intervention provider	Pharmacist	28 (58.3%)			
	Pharmacist- Physician	7 (14.6%)	Sample size (n)	≤100	6 (12.5%)
	Nurse	5 (10.4%)		101–300	16 (33.3%)
	General Practitioner	2 (4.2%)		301–500	10 (20.8%)
	Electronic tool	2 (4.2%)		>500	16 (33.3%)
	Physician-Nurse	1 (2.1%)	Follow-up period (months)	≤ 3	10 (20.8%)
	Pharmaceutical consultant	1 (2.1%)		4–6	17 (35.4%)
	Pharmacist-Nurse-Geriatrician	1 (2.1%)		7–12	18 (37.5%)
	Health and social care counsellor	1 (2.1%)		> 12	3 (6.2%)

Variable	Categories	Studies, *n* (%)	Variable	Categories	Studies *n*/*N* (%)
Patient characteristics					
Age	60–65	9 (18.7%)	Comorbidities (Mean/median)	Total:	**13/48 (27.1%)**
	66–75	19 (39.6%)			
	76–85	19 (39.6%)		< 4	5/13 (38.5%)
	> 85	1 (2.1%)		4–6	5/13 (38.5%)
Female gender	< 40%	4 (8.3%)		> 6	3/13 (23.1%)
	40–59%	28 (58.3%)			
	60–75%	13 (27.1%)	Comorbidities-Qualitative reporting	Cardiovascular disease	6/48 (12.5%)
	> 75%	2 (4.2%)		Respiratory disease	3/48 (6.2%)
	Not provided	1 (2.1%)		Hypertension	6/48 (12.5%)
Number of medications	3–5	4 (8.3%)		Diabetes	3/48 (6.2%)
	6–10	25 (52.1%)		Chronic Kidney Disease	2/48 (4.2%)
	>10	9 (18.7%)			
	Not provided	10 (20.8%)	Comorbidities	Not provided	21/48 (43.8%)

^1^Denmark, Germany, Netherlands, Northern Ireland, Republic of Ireland, Portugal, Sweden; ^2^Brazil, Chile, India, Iran, Malaysia, Sweden, Switzerland. Bold values highlight the two main analytical groups (community and transitional care) and the aggregated total for reported comorbidities.

### Setting

Most interventions were implemented in community-based settings (37/48, 77.1%), including primary care, secondary care, community pharmacies, and home-based interventions.

The remaining studies were conducted in transitional care (11/48, 22.9%), primarily initiated at hospital discharge with follow-up in the community.

### Participants

There was a wide range of sample sizes from 59 to 4,960 participants. A total of 28,146 participants were included; follow-up periods varied from 1 to 24 months. Of the 48 included studies, 41 (85.4%) reported the mean age of participants, ranging from 60.1 ± 11.7 to 85.5 ± 4.0 years. In four of these studies, the standard deviation was not reported, with a maximum mean age of 86.2 years (39). Six studies (12.5%) reported age as median [IQR], and one study (2.1%) provided age as percentage distributions. Gender was reported in 47 studies (97.9%), with the proportion of females ranging from 22.5 to 78.0%.

Comorbidities were quantitatively reported (mean or median) in 13 studies (31.2%), and 4 studies (8.3%) used comorbidity scores, such as ISAR, Charlson, or CIRS (40–43). Two studies (4.16%) reported comorbidities categorically, with most participants having one or two comorbidities (44), and another (45) reported that 53.0% of participants in the intervention group and 55.2% in the control group had at least one comorbid condition. Eight studies (16.6%) provided qualitative data on the distribution of chronic conditions, mainly cardiovascular or respiratory diseases, and hypertension, while 21 (39.6%) targeted multimorbid patients without providing details.

The mean number of medications was reported in 33 studies (68.7%), with a range of 3.7 ± 2.34 (46) to 17.55 ± 4.10 (47). Five studies (34, 45, 48–50) reported median values, ranging from 7 [5–8] (49) to 8.3 [7.4–9.3] (50). Ten studies (16.3%) did not provide data on medication numbers. Other variables, such as marital status, education level, dependency, and caregiver involvement, were also reported where available (Supplementary Tables S4a,b).

### Interventions

Most studies did not specify whether interventions targeted patients or caregivers. Details on interventions are in Supplementary Table S5. They were categorized as: medication management strategies, educational interventions, and digital/technology tools. Adherence support tools were considered as complementary strategies. One study used an integrated health and social care model, combining medication support with social, functional, and environmental components (39).

Medication management strategies were featured in 36 of the 48 included studies, with medication review being the most common (32–37, 40, 42, 43, 45, 47–49, 51–72). Educational interventions were central to 34 studies (32–35, 37, 38, 43–46, 48–52, 56, 57, 60–65, 67, 69–79). The use of technology and digital tools was less prevalent, appearing in five studies (38, 42, 55, 59, 74). Adherence support tools were employed in 25 studies (32–36, 38, 43, 45, 48, 49, 51, 52, 54–57, 62, 65, 72–77, 79, 80). Pharmacists were the main providers, leading 28 studies (58.3%) (Supplementary Tables S4a,b).

#### Quality appraisal

**Figure 2:**
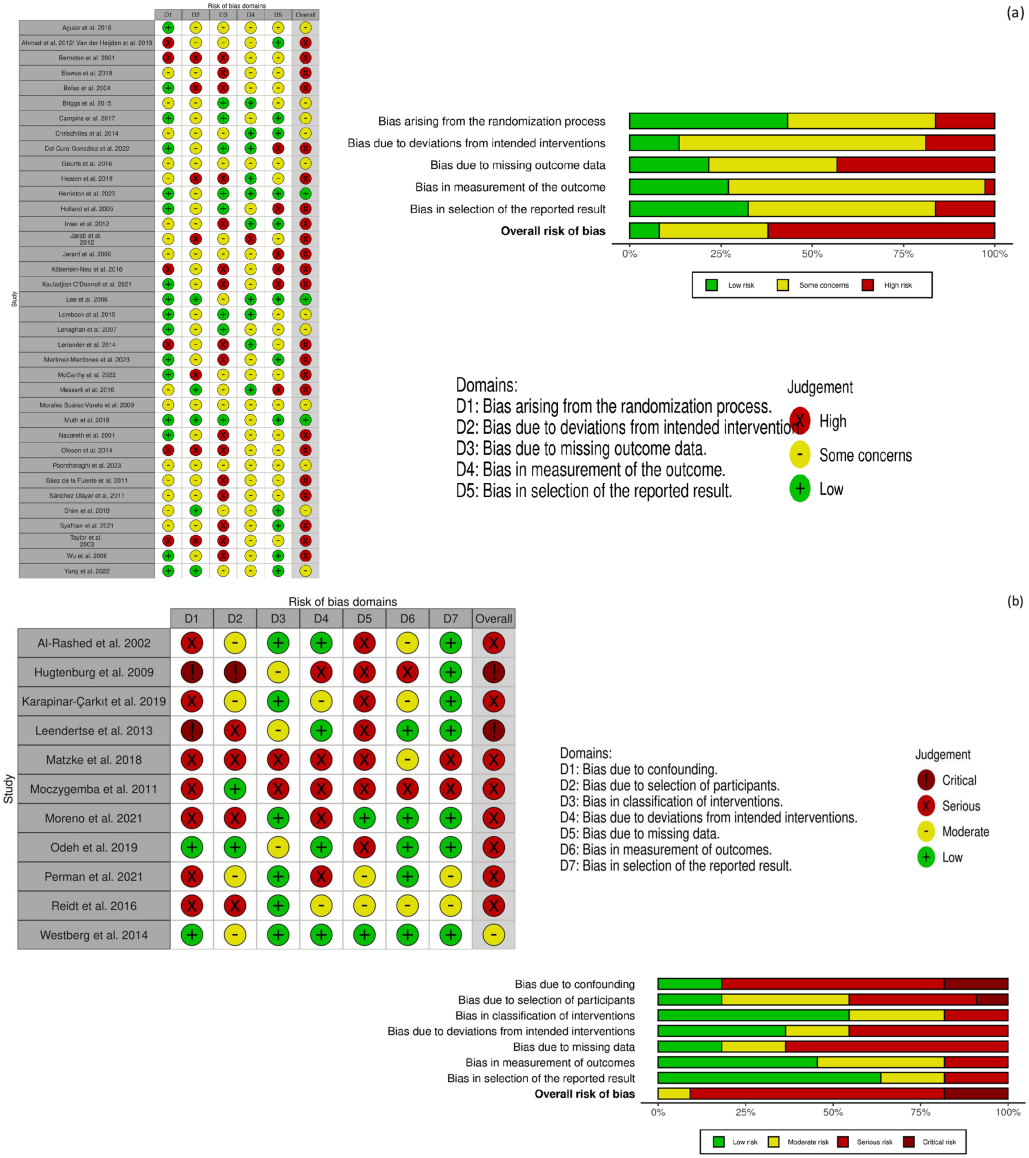
**(a)** Risk of bias summary for randomised controlled trials (RoB 2 tool). Visual representation of the risk of bias domains across included RCTs, assessed using the RoB 2 tool. **(b)** Risk of bias summary for quasi-experimental studies (ROBINS-I tool). Visual summary of risk of bias assessments across non-randomised studies, evaluated with the ROBINS-I tool.

Of 37 RCTs, 59.5% had a high risk of bias, 32.4% had some concerns, and 8.1% had a low risk of bias. Risk of bias related to randomization was the lowest, while the risk due to missing outcome data was the highest (Supplementary Table S6a; Figure 2a).

For the 11 quasi-experimental studies, 81.8% showed a high risk of bias: 54.5% had a serious risk of bias, while 27.3% were rated as critical risk of bias. Only 18.5% had a moderate risk of bias (Supplementary Table S6b; Figure 2b). The highest risk domains were related to confounding factors and missing data, whereas the lowest risk concerned outcome measurement and selective reporting of results.

#### Certainty of evidence

We graded the certainty of the evidence for adherence (continuous and dichotomous), readmissions, mortality, and quality of life. As shown in the GRADE Summary of Findings table (Table 2), all outcomes were rated as very low certainty, mainly due to serious risk of bias, inconsistency in results across studies, and imprecision in the effect estimates.

**Table 2:** Summary of findings for interventions aimed at improving medication management versus usual care for improve medication adherence and reduce other adverse health outcomes.

Outcome and follow-up	Patients (studies), *N*	Relative effect (95% CI)	Absolute effects (95% CI)			Certainty
			Usual care	Interventions aimed at improving medication management	Difference	
*Adherence*, MMAS-4 Follow-up: range 1 months to 12 months	1,414 (5 RCTs)	OR = 1.55 (1.05 to 2.28)	412 per 1,000	491 per 1,000 (0 to 0)	110 more per 1,000 (from 10 more to 200 more)	⨁◯◯◯ Very low^a^^,^^b^^,^^c^
*Adherence-Continuous Measures*, MMAS-4, VASAD, MARS, MARS-5 Follow-up: range 1 months to 9 months	2,496 (6 RCTs, 1 non-randomised study)	–	–	–	0.0 (−0.08 to 0.09)	⨁◯◯◯ Very low^d^^,^^e^^,^^f^
*Readmissions* (Number of patients with ≥ 1 readmission) Follow-up: range 1 months to 6 months	3,404 (7 RCTs, 4 non-randomised studies)	OR = 0.68 (0.41 to 1.10)	328 per 1,000	249 per 1,000 (167 to 349)	79 fewer per 1,000 (from 161 fewer to 21 more)	⨁◯◯◯ Very low^g^^,^^h^^,^^i^
*Quality of Life*, EQ-5D Follow-up: range 3 months to 12 months	2,229 (7 RCTs)	–	–	–	−0.03 (−0.14 to 0.08)	⨁◯◯◯ Very low^j^^,^^k^^,^^l^
*Deaths* Follow-up: range 1.6 months to 24 months	4,355 (7 RCTs, 4 non-randomised studies)	OR = 0.94 (0.77 to 1.15)	105 per 1,000	99 per 1,000 (83 to 119)	6 fewer per 1,000 (from 22 fewer to 14 more)	⨁◯◯◯ Very low^m^^,^^n^

CI, confidence interval; OR, odds ratio; SMD, standardised mean difference.

a 3 studies high risk, 2 some concerns; downgraded one level.

b Moderate, non-significant heterogeneity (I^2^ = 52.4%; *p* = 0.078) and variable MMAS-4 narrative results indicate inconsistency; downgraded one level.

c Borderline overall effect, wide confidence intervals and small total sample size reduce certainty; downgraded one level.

d 4 studies high risk, 2 some concerns, 1 low risk; downgraded one level.

e Despite low statistical heterogeneity (I^2^ = 2%), narrative synthesis showed clear inconsistency: several studies reported significant improvements in adherence, whereas most found no difference; downgraded one level.

f Confidence intervals including both minimal benefit and minimal harm and limited total sample size reduce certainty; downgraded one level.

g 7 studies high risk, 2 moderate risk, 2 some concerns; downgraded one level.

h Substantial heterogeneity (I^2^ = 82%) with effects varying from protective to null/reversed; subgroup differences significant, downgraded 1 level.

i The 95% CI crosses the line of no effect and includes both minimal benefit and harm; downgraded one level.

j 4 studies high risk, 1 low risk, 1 some concerns; downgraded one level.

k The EQ-5D is a broad, generic quality-of-life measure and may lack sensitivity to capture the specific effects expected from medication-management interventions; downgraded one level.

l The 95% CI crosses the line of no effect and includes both minimal benefit and harm; downgraded one level.

m 8 studies high risk, 2 some concerns, 1 moderate; downgraded 2 levels.

n The 95% CI crosses the line of no effect and includes both minimal benefit and harm; downgraded one level.

#### Outcomes

##### Primary outcomes

The findings regarding primary outcomes are detailed in Supplementary Table S7.

##### Adherence measurement

###### Morisky Medication Adherence Scale

**Figure 3:**
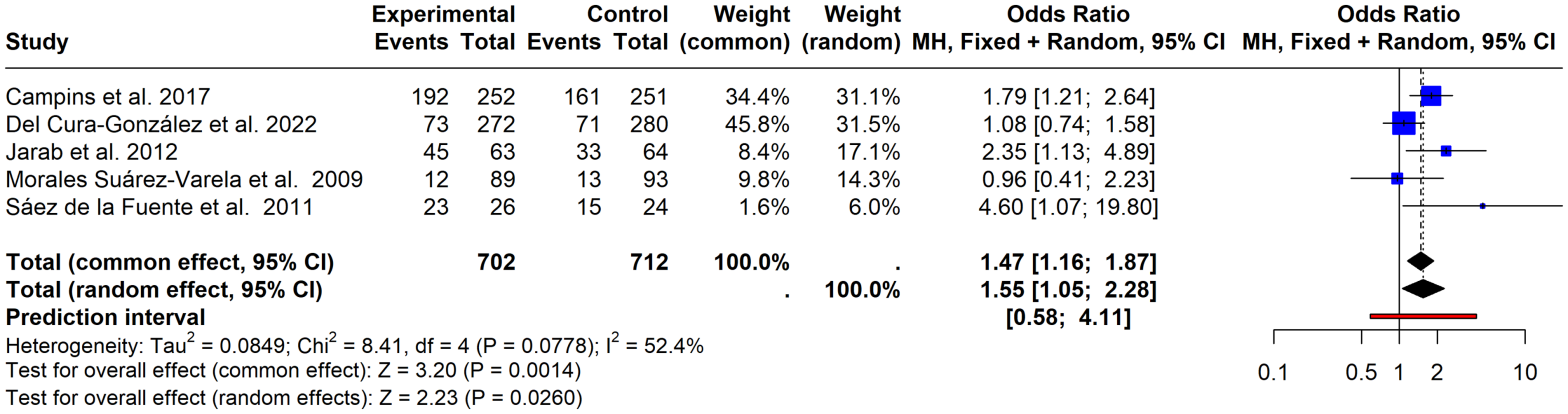
Forest plot of the effect of interventions on medication adherence (MMAS-4 scale). Pooled odds ratios with 95% confidence intervals from included studies using the MMAS-4 adherence measure. Random-effects model.

For the MMAS-4, five studies (45, 50, 53, 54, 77) were meta-analyzed, producing a statistically significant effect in favor of the intervention (OR = 1.55; 95% CI 1.05–2.28; *p* = 0.026). Heterogeneity was moderate but not statistically significant (I^2^ = 52.4%; *p* = 0.078), suggesting some variability among studies (Figure 3).

**Figure 4:**
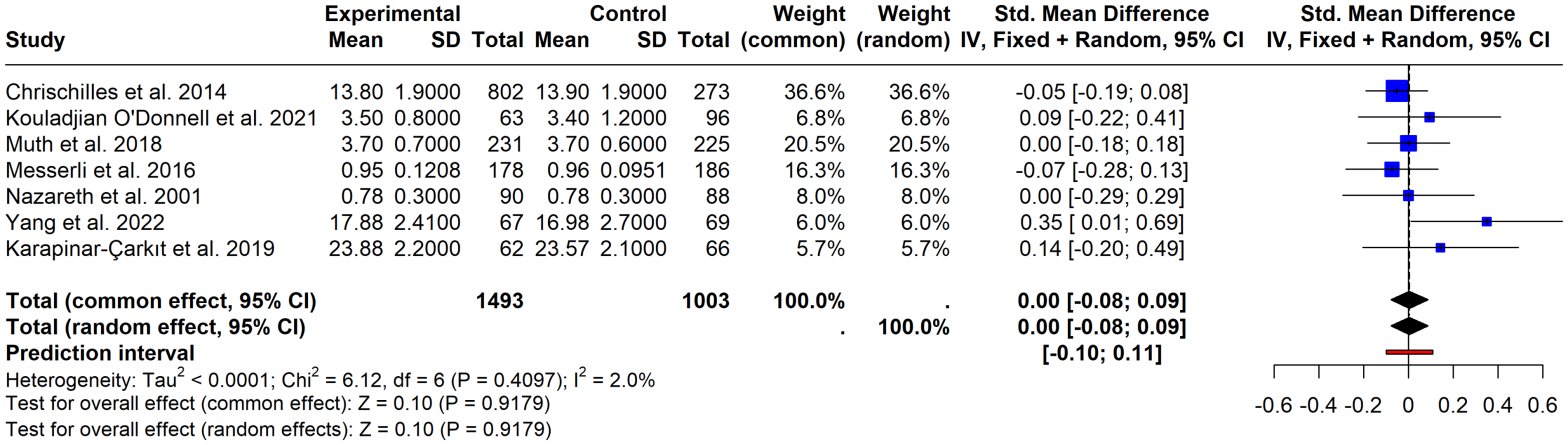
Forest plot of continuous adherence outcomes. Standardised mean differences (SMDs) with 95% confidence intervals from studies reporting adherence as a continuous variable.

###### Objective Methods

For objective methods, most studies did not provide an effect size. Methods like pill count or Medication Possession Ratios (MPR) showed comparable adherence between groups.

One study (42) reported a near-significant effect at 6 months regarding the Drug Score (OR = 0.7; 95% CI = 0.5–1.0), which disappeared at 9 months. Due to the variability in methods, these results were unsuitable for meta-analysis.

###### Other self-report tools and scales

Among the 12 (34, 35, 37, 46, 62–64, 70, 72, 73, 78, 80) studies employing other self-report tools to measure medication adherence, six demonstrated a beneficial effect (34, 37, 63, 70, 73, 78). Within this group, two studies used mixed methods to assess adherence: one combined self-report and refill records (72), and another used self-report, pill count, and computerized monitoring (78).

A meta-analysis was conducted focusing on continuous measures, including only 7 articles that provided sufficient data (35, 42, 46, 59, 62, 74, 80). The results, displayed in Figure 4 (SMD = 0.00; 95% CI = −0.08–0.09; *p* = 0.9179), with very low and non-significant heterogeneity (I^2^ = 2%; *p* = 0.4097), suggested no effect.

##### Adverse health outcomes measurement

Of the 38 studies reporting one or more adverse health outcomes, 33 addressed healthcare utilization.

###### Specialized care

Of the seven studies examining patients with one or more hospitalizations (33, 35, 38, 40, 44, 46, 60), two studies (39, 45) found statistically significant reduction in hospitalizations, while the other two (50, 51) noted trends suggesting increased hospitalizations in the intervention group.

Among the 10 studies assessing the total number of hospitalizations (42, 44, 46, 54, 60, 61, 64, 66, 67, 69), two (64, 67) reported statistically significant reductions in the intervention groups. A meta-analysis including three studies providing comparable quantitative data showed no significant effect (SMD = 0.06; 95% CI − 0.29 to 0.40; *p* = 0.74), with substantial and statistically significant heterogeneity (I^2^ = 69.5%; *p* = 0.038) (Supplementary Figure S1). One additional study, Perman et al. (39), assessed time to first hospitalization using electronic health records and reported a significantly lower hazard of first admission in the intervention group.

**Figure 5:**
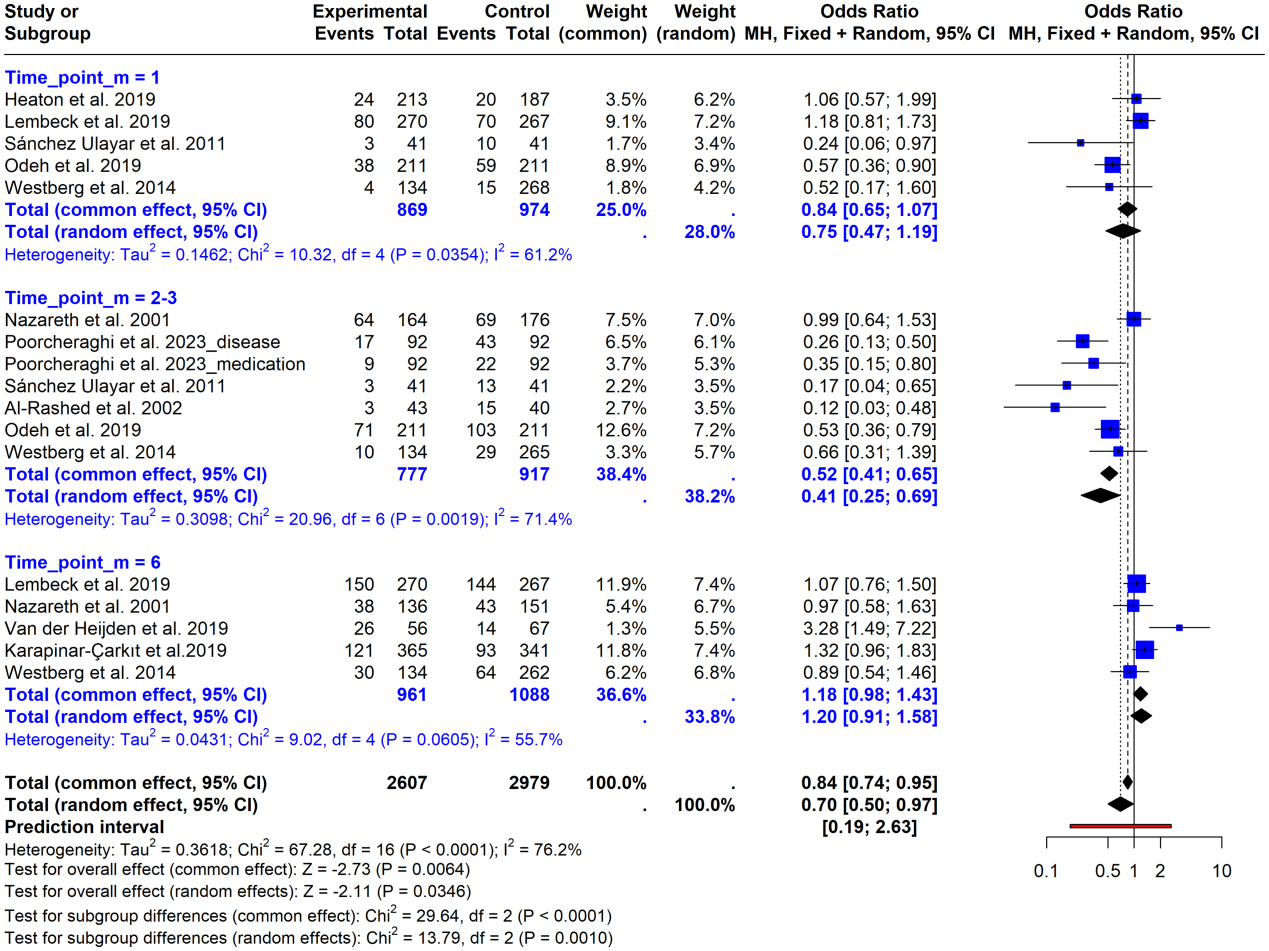
Forest plot of hospital readmissions stratified by follow-up duration. Odds ratios for readmissions at short-, medium-, and long-term follow-up intervals.

**Figure 6:**
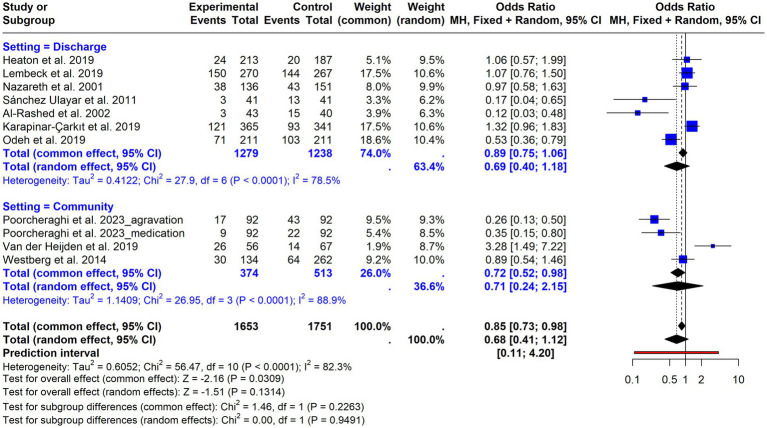
Forest plot of hospital readmissions stratified by intervention setting (community-based vs. discharge/transitional care).

For readmissions, the overall random-effects meta-analysis (Supplementary Figure S2) showed no statistically significant effect of the intervention (OR = 0.68; 95% CI = 0.41–1.10; *p* = 0.13), with substantial heterogeneity (I^2^ = 82.3%; *p* < 0.0001). Stratified analysis by follow-up duration (Figure 5) showed no significant differences for short-term follow-up (OR = 0.75; 95% CI = 0.47–1.19), with moderate and statistically significant heterogeneity (I^2^ = 61.2%; *p* = 0.035). A protective and clearly significant effect was observed for studies with a follow-up period of 2–3 months (OR = 0.41; 95% CI = 0.25–0.69), heterogeneity remained substantial and statistically significant (I^2^ = 71.4%; *p* = 0.0019). The effect reversed and lost significance for studies with a follow-up of 6 months (OR = 1.20; 95% CI = 0.91–1.58), with moderate but not statistically significant heterogeneity (I^2^ = 55.7%; *p* = 0.0605). The test for subgroup differences was statistically significant (Chi^2^ = 13.79; df = 2; *p* = 0.001), indicating that follow-up duration contributed to between-study variability. Additional stratified analysis by intervention setting (community vs. discharge; Figure 6) showed no significant differences between subgroups (Chi^2^ = 0.00; df = 1; *p* = 0.95), indicating that the setting did not explain the observed heterogeneity.

**Figure 7:**
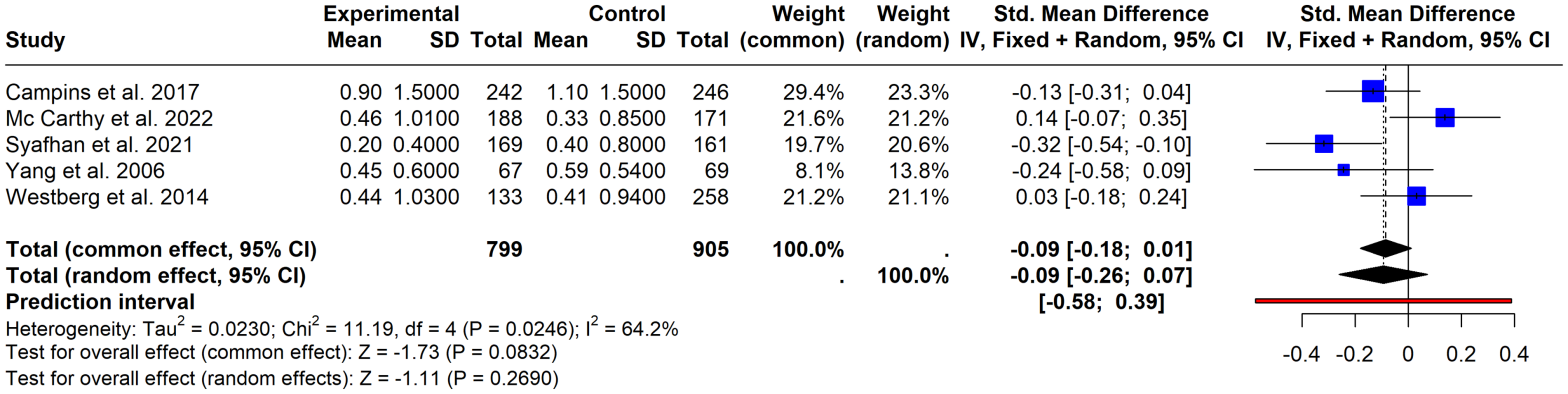
Forest plot of emergency department (ED) visits. Pooled effect of interventions on the frequency of ED visits. High heterogeneity prevented firm conclusions.

Regarding ED visits, only five studies (46, 47, 53, 64, 71) could be included in the meta-analysis, but moderate to strong heterogeneity prevented conclusive results (Figure 7).

Nine studies compared length of stay (40–42, 46, 47, 51, 61, 64, 70), mostly showing no significant differences between groups. Some suggested trends of longer stays in the intervention group (40, 41, 46, 47), and one found that the intervention group had a significantly longer length of stay compared to the control group (70).

###### Contacts with Primary Care

**Figure 8:**
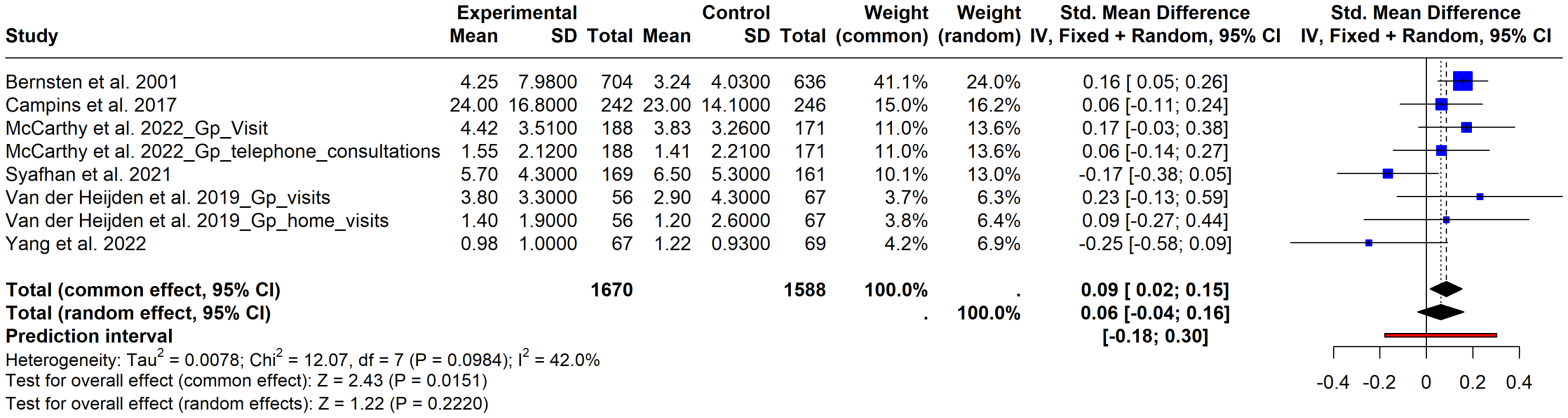
Forest plot of primary care contacts. Effect of interventions on the use of primary care services based on pooled data from included studies.

Under this category, we grouped: Primary Care visits (46, 50, 53, 61), contacts with the General Practitioner (GP) (32, 34, 35, 46, 47, 54), GP Home visits (32), Primary Care Nurse consultations (54), and telephone consultations (47, 64). Six studies provided data that could be synthesized (32, 34, 46, 47, 53, 64). The results (Figure 8) showed a weak but not significant effect in favor of an increased number of contacts in the intervention group (SMD = 0.06; 95% CI = −0.04- 0.16; *p* = 0.22), with moderate but not significant heterogeneity (I^2^ = 42%, *p* = 0.10).

DRPs (33, 55, 58, 61, 64, 74), ADRs (54), ADEs (61, 66), and ADWEs (36, 47) were also assessed. Some studies used related terminology or alternative classification systems (medication-related problems [MRPs], medication and health-related problems [MHRPs], or the Pharmaceutical Care Network Europe [PCNE] classification), while one study (72) reported medication misadventures, a composite outcome including (72) medication errors, adverse drug events, and adverse drug reactions. These differences limited comparability across studies.

Other adverse health outcomes, summarized in Supplementary Table S7, could not be meta-analyzed or did not provide results of interest (Supplementary Figures S3,S4).

#### Secondary outcomes

**Figure 9:**
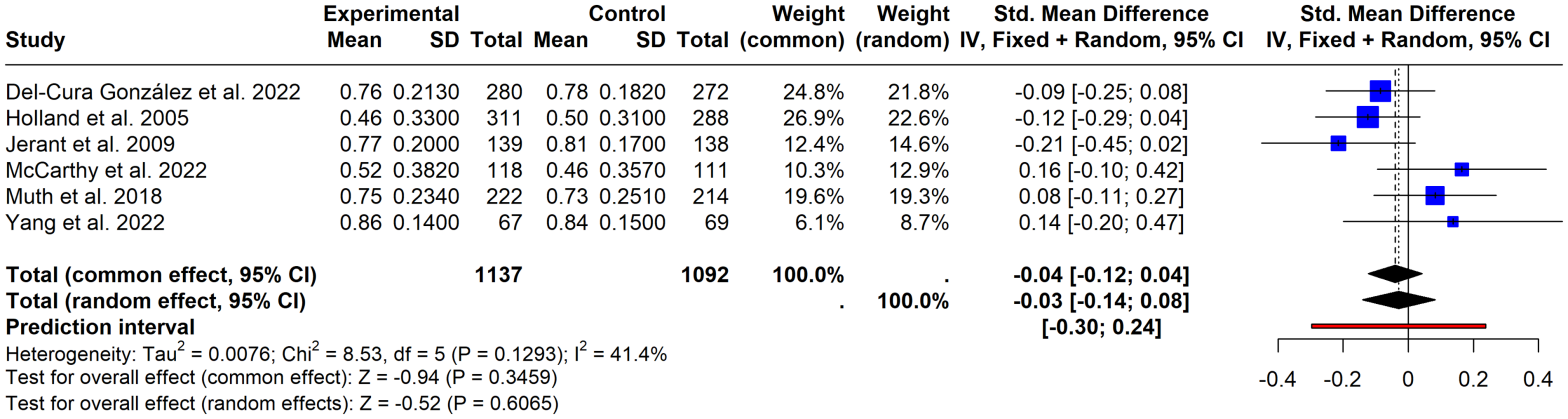
Forest plot of quality-of-life outcomes (EQ-5D). Pooled mean differences with 95% confidence intervals for EQ-5D scores reported across included studies.

Regarding quality of life, six (42, 44, 46, 47, 54, 57) out of 10 studies were meta-analyzed, revealing no significant effect of the interventions (SMD = −0.03; 95% CI = −0.14 to 0.08; *p* = 0.61), with moderate non-significant heterogeneity (I^2^ = 41.4%, *p* = 0.13) (Figure 9). Six studies evaluated patients’ beliefs and attitudes towards medication (42, 46, 62, 64, 70, 80), all using the Beliefs about Medicines Questionnaire (BMQ). Most found no significant differences between groups regarding patients’ beliefs and attitudes towards medication, although one (70) reported a significant positive effect on reducing concerns and improving the necessity-concerns balance (see Table 2).

**Figure 10:**
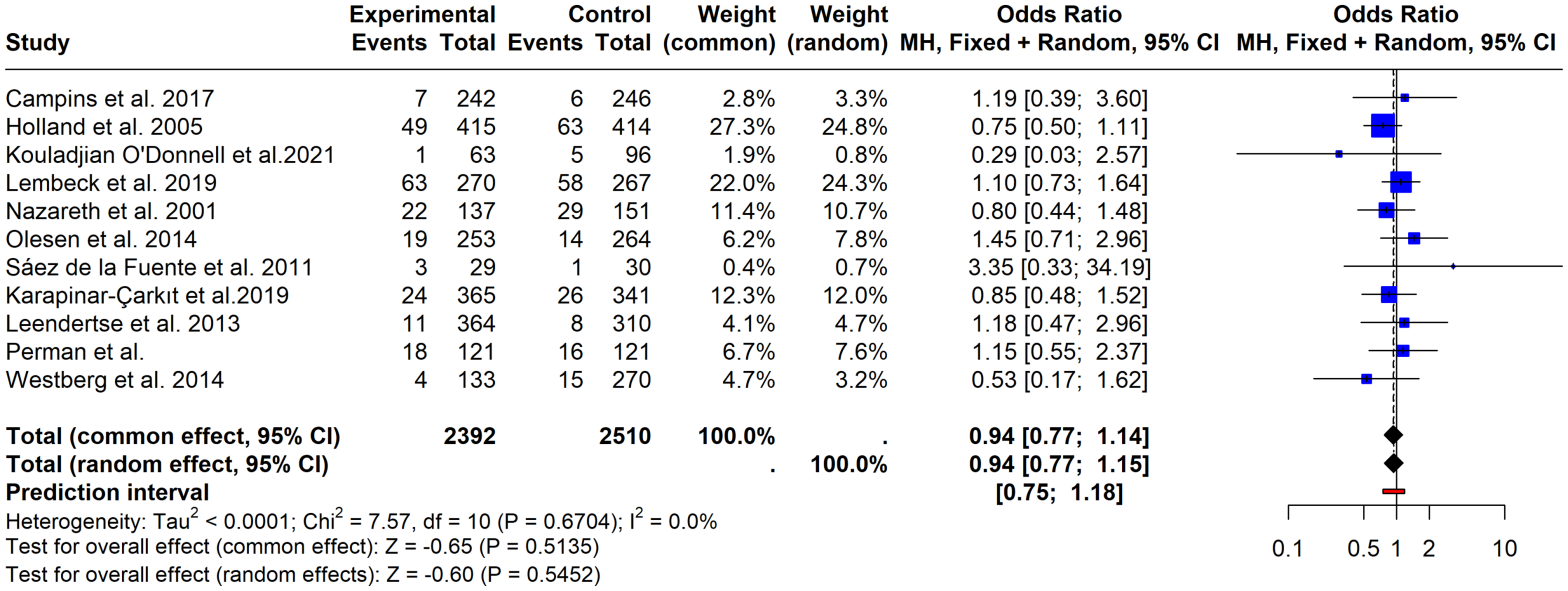
Forest plot of mortality outcomes. Pooled odds ratios with 95% confidence intervals for all-cause mortality across included studies.

Four studies assessed patients’ knowledge of medicines through various methods (34, 35, 46, 72). Most found no significant differences between groups, although Taylor et al. (72) reported a 36% higher mean knowledge score in the intervention group at 12 months, while the control group showed a 15% reduction. Three assessed mobility or functional status (42, 58, 59), and three analyzed falls (38, 59, 62), with only one (38) observed a positive impact. Nine studies addressed healthcare costs (32, 34, 44, 46, 61, 64, 67, 68, 70), but conceptual differences prevented results synthesis. Finally, twelve studies reported mortality (35, 39, 41, 49, 50, 53, 57, 59, 65, 66, 71, 80). Results from the meta-analysis, including eleven studies, showed no significant effect (OR = 0.94; 95% CI 0.77–1.15; *p* = 0.54), with no evidence of heterogeneity (I^2^ = 0%; *p* = 0.67). Given the consistency across studies, no subgroup analyses were conducted for this outcome (Figure 10). Secondary outcomes are summarized in Supplementary Table S8.

#### Publication bias

Funnel plots for readmissions (Supplementary Figure S5) and mortality (Supplementary Figure S6) appeared symmetrical, and Peters’ test for both outcomes suggested significant small-study effects (*p* > 0.10), indicating no evidence of publication bias.

Supplementary Table S9 summarizes the tools, methods, and metrics used for outcome assessment.

## Discussion

This review aimed to collate evidence on interventions to improve medication management for multimorbid older adults living in the community. Optimizing medication management in this population is a public health priority, given the challenges of ageing societies and polypharmacy. Overall, the interventions showed limited and variable effects. While a modest improvement in adherence was evident when measured by the MMAS-4, this benefit was not consistent across other adherence measures. A medium-term reduction in readmissions was observed; however, this effect dissipated and tended to reverse at 6 months, indicating that the initial benefit was not sustained over time. This attenuation may be related to the short duration and unidimensional nature of many interventions, which were generally limited in time, focused on a single aspect of care, and not adapted to patients’ changing needs. There was no significant effect on quality of life or mortality. Heterogeneity in ED visits prevented conclusive results, and primary care contacts showed a weak, non-significant increase. Inconsistent outcome measurement limited conclusions on DRPs and healthcare costs. Overall, methodological quality was low to moderate.

Strengths include broad outcome assessment and systematic quality evaluation using validated tools (28, 29), including the GRADE (31) approach to assess the certainty of evidence. Diverse intervention types provided insights across various healthcare settings. No language restrictions reduced language bias. Meta-analyses identified trends where possible.

Some limitations must be acknowledged. Although the search strategy covered PubMed, Web of Science, and CINAHL, and was expanded to include ClinicalTrials.gov and manual reference screening, studies from other databases or grey literature may have been missed, so publication bias cannot be excluded. The inclusion of exploratory outcomes, healthcare encounters not specified in the PROSPERO protocol, may have introduced selective reporting bias, though it also provided complementary insights into medication-related healthcare use.

Further limitations include heterogeneity and a lack of standardized classifications for interventions and outcomes, complicating overall conclusions and assessment of multifaceted interventions. Variations in settings and resources, small sample sizes, short follow-up, and methodological differences limited comparability. Lack of stratification by gender, socioeconomic status, or caregiver type may have masked subgroup effects. Random-effects models provided conservative estimates. Given these limitations, findings should be interpreted with caution, and future research must aim for greater methodological rigor and standardization.

The potential influence of bias on the results cannot be quantified, but it can affect results in different ways depending on the extent of missing information and group differences. In addition, many trials had some concerns for selective reporting, which could overestimate positive findings. Overall, they are likely to have reduced the certainty of the evidence.

As most studies had a moderate to high risk of bias, conducting sensitivity analyses was not feasible without substantially reducing the available evidence. Instead, the certainty of the evidence was downgraded to reflect these methodological limitations and to ensure that the potential impact of bias was considered in the interpretation of the findings.

Findings on adherence align with Burgos-Alonso et al. (81) and Cross et al. (26), both reporting inconclusive results, likely due to similar inclusion criteria. In contrast, Roncal-Belzunce et al. (82) reported some benefits, especially with collaborative nursing approaches, possibly due to a combined analysis of objective and subjective measures. For ED visits, we found no significant reduction and high heterogeneity, in contrast to other authors (26, 82) who suggested reductions in ED visits with mixed interventions, combining ED and hospital admissions data (26). Overall, these discrepancies across studies underscore the influence of methodological heterogeneity, particularly in the content, intensity, and delivery format of interventions, as well as variability in adherence measurement tools, healthcare settings, types of providers involved, and length of follow-up. In addition, the absence of a core outcome set for multimorbidity and polypharmacy research limits comparability and may partly explain the inconsistent results observed.

Our results for mortality and quality of life are consistent with Roncal-Belzunce et al. (82) who found no significant effects. While they reported DRP improvements, we could not meta-analyze due to inconsistent reporting.

From an economic perspective, our findings align with Laberge et al. (83), who found inconclusive evidence on nursing intervention costs. Roncal-Belzunce et al. (82). also noted potential reductions in medication-related costs, but not total care costs. While economic aspects are essential for clinical decision-making, detailed cost-effectiveness analyses were beyond the scope of this review and should be addressed in future studies.

### Implications for clinical practice and research

Improving adherence and outcomes requires addressing patient- and system-level factors. Health status, beliefs, behaviors, and motivations shape adherence (84, 85) and require tailored strategies, while system factors, including the healthcare environment, care accessibility, and provider support, are equally important (85).

At the core lies the patient-caregiver dyad, bridging the patient-system gap. Informal caregivers, central to home medication management, often lack training and support (86). Effective interventions should be collaborative, tailored to socio-health needs, and reflect varying health literacy (87–89), ensuring active involvement of both patients and caregivers in decision-making (90). Social, demographic, and cultural factors, especially gender, as women often assume unsupported caregiving roles, must also be considered (91).

Caregivers must be integrated into healthcare strategies (92) through training and tailored tools (86). Overcoming structural barriers, improving provider training, and ensuring transparent, well-coordinated transitional care are crucial (93), particularly in home settings where oversight is limited and caregiver dependence is high (94).

This review highlights persistent gaps in the evidence and offers insights to guide future research and clinical practice. Future research should standardize outcomes, evaluate subgroup and caregiver program impacts, and conduct economic evaluations to guide policy.

## Data Availability

The original contributions presented in the study are included in the article/Supplementary material, further inquiries can be directed to the corresponding author.
