# Supplementary material for: Interventions to enhance in-home taking medication among older adults with multimorbidity/polypharmacy: a systematic review and meta-analysis
Source: Front Public Health. 2026 Jan 28;13:1701622. doi: 10.3389/fpubh.2025.1701622 (PMC12891206; doi:10.3389/fpubh.2025.1701622)
Supplement: Supplementary file 1 [file Data_Sheet_1.zip › Supplementary Table 1.PRISMA_2020_checklist.pdf]

PRISMA 2020 Checklist

Section and Topic	Item #	Checklist item	Location where item is reported
**TITLE**			
Title	1	Identify the report as a systematic review.	Title page
**ABSTRACT**			
Abstract	2	See the PRISMA 2020 for Abstracts checklist.	Abstract, lines 1-26
**INTRODUCTION**			
Rationale	3	Describe the rationale for the review in the context of existing knowledge.	Introduction, lines 31-52
Objectives	4	Provide an explicit statement of the objective(s) or question(s) the review addresses.	Introduction, lines 52-55
**METHODS**			
Eligibility criteria	5	Specify the inclusion and exclusion criteria for the review and how studies were grouped for the syntheses.	Methods, Inclusion and exclusion criteria (Study design, population, intervention, comparator, outcomes), lines 74-107
Information sources	6	Specify all databases, registers, websites, organisations, reference lists and other sources searched or consulted to identify studies. Specify the date when each source was last searched or consulted.	Methods, Search methods, lines 60-73
Search strategy	7	Present the full search strategies for all databases, registers and websites, including any filters and limits used.	Methods, Search methods lines 60-73
Selection process	8	Specify the methods used to decide whether a study met the inclusion criteria of the review, including how many reviewers screened each record and each report retrieved, whether they worked independently, and if applicable, details of automation tools used in the process.	Methods, Study selection, lines 108-114
Data collection process	9	Specify the methods used to collect data from reports, including how many reviewers collected data from each report, whether they worked independently, any processes for obtaining or confirming data from study investigators, and if applicable, details of automation tools used in the process.	Methods, Data extraction, lines 115-118
Data items	10a	List and define all outcomes for which data were sought. Specify whether all results that were compatible with each outcome domain in each study were sought (e.g. for all measures, time points, analyses), and if not, the methods used to decide which results to collect.	Methods, Inclusion and exclusion criteria (Outcomes), lines

PRISMA 2020 Checklist

Section and Topic	Item #	Checklist item	Location where item is reported
			92-107
	10b	List and define all other variables for which data were sought (e.g. participant and intervention characteristics, funding sources). Describe any assumptions made about any missing or unclear information.	Methods, Data extraction, lines 115-118
Study risk of bias assessment	11	Specify the methods used to assess risk of bias in the included studies, including details of the tool(s) used, how many reviewers assessed each study and whether they worked independently, and if applicable, details of automation tools used in the process.	Methods, Quality assessment, lines 119-123
Effect measures	12	Specify for each outcome the effect measure(s) (e.g. risk ratio, mean difference) used in the synthesis or presentation of results.	Methods, Quantitative analysis, lines 124-147
Synthesis methods	13a	Describe the processes used to decide which studies were eligible for each synthesis (e.g. tabulating the study intervention characteristics and comparing against the planned groups for each synthesis (item #5)).	Methods, study selection, lines 108-114
	13b	Describe any methods required to prepare the data for presentation or synthesis, such as handling of missing summary statistics, or data conversions.	Not reported
	13c	Describe any methods used to tabulate or visually display results of individual studies and syntheses.	Methods, Quantitative analysis, lines 124-147
	13d	Describe any methods used to synthesize results and provide a rationale for the choice(s). If meta-analysis was performed, describe the model(s), method(s) to identify the presence and extent of statistical heterogeneity, and software package(s) used.	Methods, Quantitative analysis, lines 124-147
	13e	Describe any methods used to explore possible causes of heterogeneity among study results (e.g. subgroup analysis, meta-regression).	Methods, Quantitative analysis, lines 124-147
	13f	Describe any sensitivity analyses conducted to assess robustness of the synthesized results.	Methods, Quantitative analysis, lines 124-147
Reporting bias assessment	14	Describe any methods used to assess risk of bias due to missing results in a synthesis (arising from reporting biases).	Methods, Quantitative analysis, lines 124-147

PRISMA 2020 Checklist

Section and Topic	Item #	Checklist item	Location where item is reported
Certainty assessment	15	Describe any methods used to assess certainty (or confidence) in the body of evidence for an outcome.	Methods, Certainty of evidence, lines 148-152
**RESULTS**			
Study selection	16a	Describe the results of the search and selection process, from the number of records identified in the search to the number of studies included in the review, ideally using a flow diagram.	Results, lines 108-114
	16b	Cite studies that might appear to meet the inclusion criteria, but which were excluded, and explain why they were excluded.	Supplementary Table 3 (Supplementary materials)
Study characteristics	17	Cite each included study and present its characteristics.	Results, Studies, Setting, Participants, Interventions, lines 153-201<br>Table 1,<br>Supplementary Tables 4a-4b, Supplementary Table 5 (Supplementary materials)
Risk of bias in studies	18	Present assessments of risk of bias for each included study.	Results, Quality appraisal, lines 202-209, Figures 2a-2b<br>Supplementary Table 6a-6b (Supplementary materials)
Results of individual studies	19	For all outcomes, present, for each study: (a) summary statistics for each group (where appropriate) and (b) an effect estimate and its precision (e.g. confidence/credible interval), ideally using structured tables or plots.	Results, Outcomes, lines 215-302 Supplementary Tables 7,8

PRISMA 2020 Checklist

Section and Topic	Item #	Checklist item	Location where item is reported
Results of syntheses	20a	For each synthesis, briefly summarise the characteristics and risk of bias among contributing studies.	Results, Outcomes, lines 215-302<br>Study characteristics, lines 153-201<br>Quality appraisal, lines 202-209
	20b	Present results of all statistical syntheses conducted. If meta-analysis was done, present for each the summary estimate and its precision (e.g. confidence/credible interval) and measures of statistical heterogeneity. If comparing groups, describe the direction of the effect.	Results, Outcomes, lines 215-302, Figures 3-10, Supplementary Figures 1-4
	20c	Present results of all investigations of possible causes of heterogeneity among study results.	Results, Outcomes, 251-263, Figure 5 and 6
	20d	Present results of all sensitivity analyses conducted to assess the robustness of the synthesized results.	Discussion, Limitations, lines 343-346
Reporting biases	21	Present assessments of risk of bias due to missing results (arising from reporting biases) for each synthesis assessed.	Results, Publication bias, lines 303-307; Discussion, limitations, 339-346
Certainty of evidence	22	Present assessments of certainty (or confidence) in the body of evidence for each outcome assessed.	Results, Certainty of evidence. Lines 210-214, Table 2
**DISCUSSION**			
Discussion	23a	Provide a general interpretation of the results in the context of other evidence.	Discussion, lines 348-366
	23b	Discuss any limitations of the evidence included in the review.	Discussion, lines 333-347
	23c	Discuss any limitations of the review processes used.	Discussion, lines

PRISMA 2020 Checklist

Section and Topic	Item #	Checklist item	Location where item is reported
			327-332
	23d	Discuss implications of the results for practice, policy, and future research.	Discussion, Implications for clinical practice, and Research, lines 367-384
**OTHER INFORMATION**			
Registration and protocol	24a	Provide registration information for the review, including register name and registration number, or state that the review was not registered.	Methods, lines 56-59
	24b	Indicate where the review protocol can be accessed, or state that a protocol was not prepared.	Methods, lines 56-59
	24c	Describe and explain any amendments to information provided at registration or in the protocol.	Discussion, limitations, lines 330-332
Support	25	Describe sources of financial or non-financial support for the review, and the role of the funders or sponsors in the review.	Funding section, lines 395-396
Competing interests	26	Declare any competing interests of review authors.	Competing interests section, lines 393-394
Availability of data, code and other materials	27	Report which of the following are publicly available and where they can be found: template data collection forms; data extracted from included studies; data used for all analyses; analytic code; any other materials used in the review.	Supplementary Materials

From: Page MJ, McKenzie JE, Bossuyt PM, Boutron I, Hoffmann TC, Mulrow CD, et al. The PRISMA 2020 statement: an updated guideline for reporting systematic reviews. BMJ 2021;372:n71. doi: 10.1136/bmj.n71
