# Supplementary material for: Interventions to enhance in-home taking medication among older adults with multimorbidity/polypharmacy: a systematic review and meta-analysis
Source: Front Public Health. 2026 Jan 28;13:1701622. doi: 10.3389/fpubh.2025.1701622 (PMC12891206; doi:10.3389/fpubh.2025.1701622)
Supplement: Supplementary file 1 [file Data_Sheet_1.zip › Supplementary Table 2. Search Strategy_CINAHL.PDF]

S#: S35
Query (user-entered): S31 AND S32 AND S21
Query (expanded/display term): (XB elderly OR XB older adults OR XB geriatric OR XB aged OR XB seniors OR MH aged OR XB informal caregivers OR XB relatives OR XB informal carers OR family caregivers OR MH family caregivers) AND (XB medication adherence OR XB medication management OR XB medication compliance OR XB safe medication) AND (XB home care OR XB transitional care OR XB pharmaceutical care)
Search run Date and Time: 2025-10-13T14:09:28.114Z
Results (count): 332
Search Mode: Proximidad
Expander(s): Buscar también dentro del texto completo de los artículos, Aplicar materias equivalentes
Filter(-s):
Fecha de publicación (start date): 2000-01-01
Fecha de publicación (end date): 2024-07-09
Interface:
Database(s): CINAHL Complete

S#: S34
Query (user-entered): S31 AND S32 AND S21
Query (expanded/display term): (XB elderly OR XB older adults OR XB geriatric OR XB aged OR XB seniors OR MH aged OR XB informal caregivers OR XB relatives OR XB informal carers OR family caregivers OR MH family caregivers) AND (XB medication adherence OR XB medication management OR XB medication compliance OR XB safe medication) AND (XB home care OR XB transitional care OR XB pharmaceutical care)
Search run Date and Time: 2025-10-13T14:08:38.225Z
Results (count): 381
Search Mode: Proximidad
Expander(s): Buscar también dentro del texto completo de los artículos, Aplicar materias equivalentes
Interface:
Database(s): CINAHL Complete

S#: S33
Query (user-entered): XB (medication adherence) OR XB (medication management) OR XB (medication compliance) OR XB (safe medication) OR MH (medication adherence)
Query (expanded/display term): XB (medication adherence) OR XB (medication management) OR XB (medication compliance) OR XB (safe medication) OR MH (medication adherence)
Search run Date and Time: 2025-10-13T14:07:54.798Z
Results (count): 22248
Search Mode: Proximidad
Expander(s): Buscar también dentro del texto completo de los artículos, Aplicar materias equivalentes
Interface:

Database(s): CINAHL Complete

S#: S32
Query (user-entered): XB (medication adherence) OR XB (medication management) OR XB (medication compliance) OR XB (safe medication)
Query (expanded/display term): XB (medication adherence) OR XB (medication management) OR XB (medication compliance) OR XB (safe medication)
Search run Date and Time: 2025-10-13T14:07:39.894Z
Results (count): 22248
Search Mode: Proximidad
Expander(s): Buscar también dentro del texto completo de los artículos, Aplicar materias equivalentes
Interface:
Database(s): CINAHL Complete

S#: S31
Query (user-entered): XB (elderly) OR XB (older adults) OR XB (geriatric) OR XB (aged) OR XB (seniors) OR MH (aged) OR XB (informal caregivers) OR XB (relatives) OR XB (informal carers) OR family caregivers OR MH (family caregivers)
Query (expanded/display term): XB (elderly) OR XB (older adults) OR XB (geriatric) OR XB (aged) OR XB (seniors) OR MH (aged) OR XB (informal caregivers) OR XB (relatives) OR XB (informal carers) OR family caregivers OR MH (family caregivers)
Search run Date and Time: 2025-10-13T14:06:51.247Z
Results (count): 1394858
Search Mode: Proximidad
Expander(s): Buscar también dentro del texto completo de los artículos, Aplicar materias equivalentes
Interface:
Database(s): CINAHL Complete

S#: S30
Query (user-entered): XB (elderly) OR XB (older adults) OR XB (geriatric) OR XB (aged) OR XB (seniors) OR MH (aged) OR XB (informal caregivers) OR XB (relatives) OR XB (informal carers) OR family caregivers
Query (expanded/display term): XB (elderly) OR XB (older adults) OR XB (geriatric) OR XB (aged) OR XB (seniors) OR MH (aged) OR XB (informal caregivers) OR XB (relatives) OR XB (informal carers) OR family caregivers
Search run Date and Time: 2025-10-13T14:06:32.092Z
Results (count): 1394858
Search Mode: Proximidad
Expander(s): Buscar también dentro del texto completo de los artículos, Aplicar materias equivalentes
Interface:

Database(s): CINAHL Complete

S#: S29
Query (user-entered): XB (elderly) OR XB (older adults) OR XB (geriatric) OR XB (aged) OR XB (seniors) OR XB (family caregivers) OR XB (informal caregivers) OR XB (relatives) OR XB (informal carers)
Query (expanded/display term): XB (elderly) OR XB (older adults) OR XB (geriatric) OR XB (aged) OR XB (seniors) OR XB (family caregivers) OR XB (informal caregivers) OR XB (relatives) OR XB (informal carers)
Search run Date and Time: 2025-10-13T14:05:47.793Z
Results (count): 644349
Search Mode: Proximidad
Expander(s): Buscar también dentro del texto completo de los artículos, Aplicar materias equivalentes
Interface:
Database(s): CINAHL Complete

S#: S28
Query (user-entered): S27 AND S20 AND S21
Query (expanded/display term): (XB elderly OR XB older adults OR XB geriatric OR XB aged OR XB seniors OR XB family caregivers OR XB informal caregivers OR XB relatives OR XB informal carers) AND (XB medication adherence OR XB medication management OR XB medication compliance OR XB safe medication) AND (XB home care OR XB transitional care OR XB pharmaceutical care)
Search run Date and Time: 2025-10-13T13:59:59.460Z
Results (count): 230
Search Mode: Proximidad
Expander(s): Buscar también dentro del texto completo de los artículos, Aplicar materias equivalentes
Interface:
Database(s): CINAHL Complete

S#: S27
Query (user-entered): XB (elderly) OR XB (older adults) OR XB (geriatric) OR XB (aged) OR XB (seniors) OR XB (family caregivers) OR XB (informal caregivers) OR XB (relatives) OR XB (informal carers)
Query (expanded/display term): XB (elderly) OR XB (older adults) OR XB (geriatric) OR XB (aged) OR XB (seniors) OR XB (family caregivers) OR XB (informal caregivers) OR XB (relatives) OR XB (informal carers)
Search run Date and Time: 2025-10-13T13:58:46.421Z
Results (count): 644349
Search Mode: Proximidad
Expander(s): Buscar también dentro del texto completo de los artículos, Aplicar materias equivalentes
Interface:

Database(s): CINAHL Complete

S#: S26
Query (user-entered): XB (elderly) OR XB (older adults) OR XB (geriatric) OR XB (aged) OR XB (seniors) OR XB (family caregivers) OR XB (informal caregivers)
Query (expanded/display term): XB (elderly) OR XB (older adults) OR XB (geriatric) OR XB (aged) OR XB (seniors) OR XB (family caregivers) OR XB (informal caregivers)
Search run Date and Time: 2025-10-13T13:58:00.285Z
Results (count): 489327
Search Mode: Proximidad
Expander(s): Buscar también dentro del texto completo de los artículos, Aplicar materias equivalentes
Interface:
Database(s): CINAHL Complete

S#: S25
Query (user-entered): XB (elderly) OR XB (older adults) OR XB (geriatric) OR XB (family caregivers) OR XB (informal caregivers)
Query (expanded/display term): XB (elderly) OR XB (older adults) OR XB (geriatric) OR XB (family caregivers) OR XB (informal caregivers)
Search run Date and Time: 2025-10-13T13:56:44.127Z
Results (count): 241688
Search Mode: Proximidad
Expander(s): Buscar también dentro del texto completo de los artículos, Aplicar materias equivalentes
Interface:
Database(s): CINAHL Complete

S#: S24
Query (user-entered): XB (elderly) OR XB (older adults) OR XB (geriatric) OR XB (family caregivers) OR XB (informal caregivers)
Query (expanded/display term): XB (elderly) OR XB (older adults) OR XB (geriatric) OR XB (family caregivers) OR XB (informal caregivers)
Search run Date and Time: 2025-10-13T13:53:49.589Z
Results (count): 241688
Search Mode: Proximidad
Expander(s): Buscar también dentro del texto completo de los artículos, Aplicar materias equivalentes
Interface:
Database(s): CINAHL Complete

S#: S23
Query (user-entered): S19 AND S20 AND S21

Query (expanded/display term): (XB elderly OR XB older adults OR XB geriatric OR XB family caregivers OR XB informal caregivers) AND (XB medication adherence OR XB medication management OR XB medication compliance OR XB safe medication) AND (XB home care OR XB transitional care OR XB pharmaceutical care)
Search run Date and Time: 2025-10-12T17:48:23.564Z
Results (count): 166
Search Mode: Proximidad
Expander(s): Buscar también dentro del texto completo de los artículos, Aplicar materias equivalentes
Interface:
Database(s): CINAHL Complete

S#: S22
Query (user-entered): (XB (home care) OR XB (transitional care) OR XB (pharmaceutical care)) AND (S19 AND S20 AND S21)
Query (expanded/display term): (XB (home care) OR XB (transitional care) OR XB (pharmaceutical care)) AND ((XB elderly OR XB older adults OR XB geriatric OR XB family caregivers OR XB informal caregivers) AND (XB medication adherence OR XB medication management OR XB medication compliance OR XB safe medication) AND (XB home care OR XB transitional care OR XB pharmaceutical care))
Search run Date and Time: 2025-10-12T17:47:29.641Z
Results (count): 166
Search Mode: Proximidad
Expander(s): Buscar también dentro del texto completo de los artículos, Aplicar materias equivalentes
Interface:
Database(s): CINAHL Complete

S#: S21
Query (user-entered): XB (home care) OR XB (transitional care) OR XB (pharmaceutical care)
Query (expanded/display term): XB (home care) OR XB (transitional care) OR XB (pharmaceutical care)
Search run Date and Time: 2025-10-12T17:46:42.487Z
Results (count): 48977
Search Mode: Proximidad
Expander(s): Buscar también dentro del texto completo de los artículos, Aplicar materias equivalentes
Interface:
Database(s): CINAHL Complete

S#: S20
Query (user-entered): XB (medication adherence) OR XB (medication management) OR XB (medication compliance) OR XB (safe medication)

Query (expanded/display term): XB (medication adherence) OR XB (medication management) OR XB (medication compliance) OR XB (safe medication)
Search run Date and Time: 2025-10-12T17:46:03.283Z
Results (count): 22246
Search Mode: Proximidad
Expander(s): Buscar también dentro del texto completo de los artículos, Aplicar materias equivalentes
Interface:
Database(s): CINAHL Complete

S#: S19
Query (user-entered): XB (elderly) OR XB (older adults) OR XB (geriatric) OR XB (family caregivers) OR XB (informal caregivers)
Query (expanded/display term): XB (elderly) OR XB (older adults) OR XB (geriatric) OR XB (family caregivers) OR XB (informal caregivers)
Search run Date and Time: 2025-10-12T17:44:50.211Z
Results (count): 241673
Search Mode: Proximidad
Expander(s): Buscar también dentro del texto completo de los artículos, Aplicar materias equivalentes
Interface:
Database(s): CINAHL Complete

S#: S18
Query (user-entered): TX (aged) OR TX (elderly) OR TX (older adults) OR TX (geriatric) OR TX (seniors) OR TX (caregivers) OR TX (family members) OR TX (relatives) OR TX (informal caregivers)
Query (expanded/display term): TX (aged) OR TX (elderly) OR TX (older adults) OR TX (geriatric) OR TX (seniors) OR TX (caregivers) OR TX (family members) OR TX (relatives) OR TX (informal caregivers)
Search run Date and Time: 2025-10-12T17:41:28.821Z
Results (count): 2618380
Search Mode: Proximidad
Expander(s): Buscar también dentro del texto completo de los artículos, Aplicar materias equivalentes
Interface:
Database(s): CINAHL Complete

S#: S17
Query (user-entered): TX (aged) OR TX (elderly) OR TX (older adults) OR TX (geriatric) OR TX (seniors) OR TX (caregivers) OR TX (family members) OR TX (relatives) OR TX (informal caregivers)
Query (expanded/display term): TX (aged) OR TX (elderly) OR TX (older adults) OR TX (geriatric) OR TX (seniors) OR TX (caregivers) OR TX (family members) OR TX (relatives) OR TX (informal caregivers)
Search run Date and Time: 2025-10-12T17:38:54.674Z

Results (count): 2618380
Search Mode: Proximidad
Expander(s): Buscar también dentro del texto completo de los artículos, Aplicar materias equivalentes
Interface:
Database(s): CINAHL Complete

S#: S16
Query (user-entered): TX (aged) OR TX (elderly) OR TX (older adults) OR TX (geriatric) OR TX (seniors) OR MH (caregivers) OR TX (family members) OR TX (relatives) OR TX (informal caregivers)
Query (expanded/display term): TX (aged) OR TX (elderly) OR TX (older adults) OR TX (geriatric) OR TX (seniors) OR MH (caregivers) OR TX (family members) OR TX (relatives) OR TX (informal caregivers)
Search run Date and Time: 2025-10-12T17:38:45.728Z
Results (count): 2566839
Search Mode: Proximidad
Expander(s): Buscar también dentro del texto completo de los artículos, Aplicar materias equivalentes
Interface:
Database(s): CINAHL Complete

S#: S15
Query (user-entered): S6 AND S10 AND S11
Query (expanded/display term): (TX aged OR TX elderly OR TX older adults OR TX geriatric OR TX seniors OR MH caregivers OR TX family members OR TX relatives OR TX informal caregivers) AND (TX medication adherence OR TX medication management OR TX medication compliance OR TX safe medication) AND (home care OR transitional care OR pharmaceutical care)
Search run Date and Time: 2025-10-12T17:36:54.756Z
Results (count): 8044
Search Mode: Proximidad
Expander(s): Buscar también dentro del texto completo de los artículos, Aplicar materias equivalentes
Filter(-s):
Fecha de publicación (start date): 2000-01-01
Fecha de publicación (end date): 2024-07-09
Interface:
Database(s): CINAHL Complete

S#: S14
Query (user-entered): S6 AND S10 AND S11
Query (expanded/display term): (TX aged OR TX elderly OR TX older adults OR TX geriatric OR TX seniors OR MH caregivers OR TX family members OR TX relatives OR TX informal caregivers) AND (TX medication adherence OR TX

medication management OR TX medication compliance OR TX safe medication) AND (home care OR transitional care OR pharmaceutical care)
Search run Date and Time: 2025-10-12T17:36:11.682Z
Results (count): 8684
Search Mode: Proximidad
Expander(s): Buscar también dentro del texto completo de los artículos, Aplicar materias equivalentes
Interface:
Database(s): CINAHL Complete

S#: S13
Query (user-entered): (S6 AND S10) AND (S11)
Query (expanded/display term): ((TX aged OR TX elderly OR TX older adults OR TX geriatric OR TX seniors OR MH caregivers OR TX family members OR TX relatives OR TX informal caregivers) AND (TX medication adherence OR TX medication management OR TX medication compliance OR TX safe medication)) AND ((home care OR transitional care OR pharmaceutical care))
Search run Date and Time: 2025-10-12T17:35:22.010Z
Results (count): 8684
Search Mode: Proximidad
Expander(s): Buscar también dentro del texto completo de los artículos, Aplicar materias equivalentes
Interface:
Database(s): CINAHL Complete

S#: S12
Query (user-entered): (S6 AND S10 AND S11)
Query (expanded/display term): ((TX aged OR TX elderly OR TX older adults OR TX geriatric OR TX seniors OR MH caregivers OR TX family members OR TX relatives OR TX informal caregivers) AND (TX medication adherence OR TX medication management OR TX medication compliance OR TX safe medication) AND (home care OR transitional care OR pharmaceutical care))
Search run Date and Time: 2025-10-12T17:33:34.036Z
Results (count): 8684
Search Mode: Proximidad
Expander(s): Buscar también dentro del texto completo de los artículos, Aplicar materias equivalentes
Interface:
Database(s): CINAHL Complete

S#: S11
Query (user-entered): home care OR transitional care OR pharmaceutical care
Query (expanded/display term): home care OR transitional care OR pharmaceutical care
Search run Date and Time: 2025-10-12T17:32:10.951Z
Results (count): 175278

Search Mode: Proximidad
Expander(s): Buscar también dentro del texto completo de los artículos, Aplicar materias equivalentes
Interface:
Database(s): CINAHL Complete

S#: S10
Query (user-entered): TX (medication adherence) OR TX (medication management) OR TX (medication compliance) OR TX (safe medication)
Query (expanded/display term): TX (medication adherence) OR TX (medication management) OR TX (medication compliance) OR TX (safe medication)
Search run Date and Time: 2025-10-12T17:31:17.368Z
Results (count): 80381
Search Mode: Proximidad
Expander(s): Buscar también dentro del texto completo de los artículos, Aplicar materias equivalentes
Interface:
Database(s): CINAHL Complete

S#: S9
Query (user-entered): TX (medication adherence) OR medication management OR medication compliance OR safe medication OR MH (medication adherence)
Query (expanded/display term): TX (medication adherence) OR medication management OR medication compliance OR safe medication OR MH (medication adherence)
Search run Date and Time: 2025-10-12T17:30:58.465Z
Results (count): 80381
Search Mode: Proximidad
Expander(s): Buscar también dentro del texto completo de los artículos, Aplicar materias equivalentes
Interface:
Database(s): CINAHL Complete

S#: S8
Query (user-entered): TX (medication adherence) OR medication management OR medication compliance OR safe medication
Query (expanded/display term): TX (medication adherence) OR medication management OR medication compliance OR safe medication
Search run Date and Time: 2025-10-12T17:30:42.994Z
Results (count): 80381
Search Mode: Proximidad
Expander(s): Buscar también dentro del texto completo de los artículos, Aplicar materias equivalentes
Interface:

Database(s): CINAHL Complete

S#: S7
Query (user-entered): MH (medication adherence) OR medication management OR medication compliance OR safe medication
Query (expanded/display term): MH (medication adherence) OR medication management OR medication compliance OR safe medication
Search run Date and Time: 2025-10-12T17:30:32.749Z
Results (count): 65509
Search Mode: Proximidad
Expander(s): Buscar también dentro del texto completo de los artículos, Aplicar materias equivalentes
Interface:
Database(s): CINAHL Complete

S#: S6
Query (user-entered): TX (aged) OR TX (elderly) OR TX (older adults) OR TX (geriatric) OR TX (seniors) OR MH (caregivers) OR TX (family members) OR TX (relatives) OR TX (informal caregivers)
Query (expanded/display term): TX (aged) OR TX (elderly) OR TX (older adults) OR TX (geriatric) OR TX (seniors) OR MH (caregivers) OR TX (family members) OR TX (relatives) OR TX (informal caregivers)
Search run Date and Time: 2025-10-12T17:29:20.723Z
Results (count): 2566839
Search Mode: Proximidad
Expander(s): Buscar también dentro del texto completo de los artículos, Aplicar materias equivalentes
Interface:
Database(s): CINAHL Complete

S#: S5
Query (user-entered): TX (aged) OR TX (elderly) OR TX (older adults) OR TX (geriatric) OR TX (seniors) OR MH (caregivers) OR TX (family members) OR TX (relatives) OR TX (informal caregivers) OR MH (aged)
Query (expanded/display term): TX (aged) OR TX (elderly) OR TX (older adults) OR TX (geriatric) OR TX (seniors) OR MH (caregivers) OR TX (family members) OR TX (relatives) OR TX (informal caregivers) OR MH (aged)
Search run Date and Time: 2025-10-12T17:29:01.343Z
Results (count): 2566839
Search Mode: Proximidad
Expander(s): Buscar también dentro del texto completo de los artículos, Aplicar materias equivalentes
Interface:
Database(s): CINAHL Complete

S#: S4
Query (user-entered): TX (aged) OR TX (elderly) OR TX (older adults) OR TX (geriatric) OR TX (seniors) OR MH (caregivers) OR TX (family members) OR TX (relatives) OR TX (informal caregivers) OR TX (aged)
Query (expanded/display term): TX (aged) OR TX (elderly) OR TX (older adults) OR TX (geriatric) OR TX (seniors) OR MH (caregivers) OR TX (family members) OR TX (relatives) OR TX (informal caregivers) OR TX (aged)
Search run Date and Time: 2025-10-12T17:28:48.571Z
Results (count): 2566839
Search Mode: Proximidad
Expander(s): Buscar también dentro del texto completo de los artículos, Aplicar materias equivalentes
Interface:
Database(s): CINAHL Complete

S#: S3
Query (user-entered): TX (aged) OR TX (elderly) OR TX (older adults) OR TX (geriatric) OR TX (seniors) OR MH (caregivers) OR TX (family members) OR TX (relatives) OR TX (informal caregivers)
Query (expanded/display term): TX (aged) OR TX (elderly) OR TX (older adults) OR TX (geriatric) OR TX (seniors) OR MH (caregivers) OR TX (family members) OR TX (relatives) OR TX (informal caregivers)
Search run Date and Time: 2025-10-12T17:27:13.123Z
Results (count): 2566839
Search Mode: Proximidad
Expander(s): Buscar también dentro del texto completo de los artículos, Aplicar materias equivalentes
Interface:
Database(s): CINAHL Complete

S#: S2
Query (user-entered): TX (aged) OR TX (elderly) OR TX (older adults) OR TX (geriatric) OR TX (seniors) OR MH (caregivers) OR TX (family members) OR TX (relatives) OR TX (informal caregivers)
Query (expanded/display term): TX (aged) OR TX (elderly) OR TX (older adults) OR TX (geriatric) OR TX (seniors) OR MH (caregivers) OR TX (family members) OR TX (relatives) OR TX (informal caregivers)
Search run Date and Time: 2025-10-12T17:27:01.127Z
Results (count): 2566839
Search Mode: Proximidad
Expander(s): Buscar también dentro del texto completo de los artículos, Aplicar materias equivalentes
Interface:
Database(s): CINAHL Complete

S#: S1

Query (user-entered): MH (aged) OR TX (elderly) OR TX (older adults) OR TX (geriatric) OR TX (seniors) OR MH (caregivers) OR TX (family members) OR TX (relatives) OR TX (informal caregivers)
Query (expanded/display term): MH (aged) OR TX (elderly) OR TX (older adults) OR TX (geriatric) OR TX (seniors) OR MH (caregivers) OR TX (family members) OR TX (relatives) OR TX (informal caregivers)
Search run Date and Time: 2025-10-12T17:26:44.164Z
Results (count): 1902092
Search Mode: Proximidad
Expander(s): Buscar también dentro del texto completo de los artículos, Aplicar materias equivalentes
Interface:
Database(s): CINAHL Complete
