# Supplementary material for: Interventions to enhance in-home taking medication among older adults with multimorbidity/polypharmacy: a systematic review and meta-analysis
Source: Front Public Health. 2026 Jan 28;13:1701622. doi: 10.3389/fpubh.2025.1701622 (PMC12891206; doi:10.3389/fpubh.2025.1701622)
Supplement: Supplementary file 1 [file Data_Sheet_1.zip › Supplementary Table 3. Excluded_Reasons.pdf]

REASON FOR EXCLUSION	EXCLUDED STUDIES
**WRONG POPULATION (n= 18)**	
Nursing Home residents included	Bosch-Lenders D, Jansen J, Stoffers HEJHJ, Winkens B, Aretz K, Twellaar M, Schols JMGA, van der Kuy PM, Knottnerus JA, van den Akker M. The Effect of a Comprehensive, Interdisciplinary Medication Review on Quality of Life and Medication Use in Community Dwelling Older People with Polypharmacy. J Clin Med. 2021 Feb 5;10(4):600. doi: 10.3390/jcm10040600. PMID: 33562702; PMCID: PMC7915595.
Nursing Home residents included	Gustafsson M, Sjölander M, Pfister B, Jonsson J, Schneede J, Lövheim H. Pharmacist participation in hospital ward teams and hospital readmission rates among people with dementia: a randomized controlled trial. Eur J Clin Pharmacol. 2017 Jul;73(7):827-835. doi: 10.1007/s00228-017-2249-8. Epub 2017 Apr 8. PMID: 28391409; PMCID: PMC5486919.
Nursing Home residents included	Ponjee GHM, van de Meerendonk HWPC, Janssen MJA, Karapinar-Çarkit F. The effect of an inpatient geriatric stewardship on drug-related problems reported by patients after discharge. Int J Clin Pharm. 2021 Feb;43(1):191-202. doi: 10.1007/s11096-020-01133-x. Epub 2020 Sep 10. PMID: 32909222.
Nursing Home residents included	Schäfer I, Kaduszkiewicz H, Mellert C, Löffler C, Mortsiefer A, Ernst A, Stolzenbach CO, Wiese B, Abholz HH, Scherer M, van den Bussche H, Altiner A. Narrative medicine-based intervention in primary care to reduce polypharmacy: results from the cluster-randomised controlled trial MultiCare AGENDA. BMJ Open. 2018 Jan 23;8(1):e017653. doi: 10.1136/bmjopen-2017-017653. PMID: 29362248; PMCID: PMC5786138.
Nursing Home residents included	Legrain S, Tubach F, Bonnet-Zamponi D, et al. A new multimodal geriatric discharge-planning intervention to prevent emergency visits and rehospitalizations of older adults: the optimization of medication in AGEd multicenter randomized controlled trial. J Am Geriatr Soc. 2011;59(11):2017-2028. doi:10.1111/j.1532-5415.2011.03628.x.
Mean age <60	Phatak A, Prusi R, Ward B, Hansen LO, Williams MV, Vetter E, Chapman N, Postelnick M. Impact of pharmacist involvement in the transitional care of high-risk patients through medication reconciliation, medication education, and postdischarge call-backs (IPITCH Study). J Hosp Med. 2016 Jan;11(1):39-44. doi: 10.1002/jhm.2493. Epub 2015 Oct 5. PMID: 26434752.
Mean age <60	Dunn TE, Desai KJ, Krajewski MP, Jacobs DM, Lu CH, Paul S, Paladino JA. Pharmacists and transitions of care from emergency department to home. Am J Manag Care. 2023 Dec;29(12):715-719.
Mean age <60	Jameson JP, VanNoord GR. Pharmacotherapy consultation on polypharmacy patients in ambulatory care. Ann Pharmacother. 2001 Jul-Aug;35(7-8):835-40.
Mean age <60	Stefanovic SM, Jankovic SM. Knowledge of the pharmacological profile of a patient improves the quality of prescribing, the outcomes of treatment, and the utilization of health services in primary health care. Eur J Clin Pharmacol. 2011 Nov;67(11):1091-101.
Mean age <60	Walker PC, Bernstein SJ, Jones JN, Piersma J, Kim HW, Regal RE, Kuhn L, Flanders SA. Impact of a pharmacist-facilitated hospital discharge program: a quasi-experimental study. Arch Intern Med. 2009 Nov 23;169(21):2003-10.
Mean age <60	Bailey JE, Surbhi S, Wan JY, Munshi KD, Waters TM, Binkley BL, Ugwueke MO, Graetz I. Effect of Intensive Interdisciplinary Transitional Care for High-Need, High-Cost Patients on Quality, Outcomes, and Costs: a Quasi-Experimental Study. J Gen Intern Med. 2019 Sep;34(9):1815-1824.
Medication managed by nurses	Ravn-Nielsen LV, Duckert ML, Lund ML, Henriksen JP, Nielsen ML, Eriksen CS, Buck TC, Pottegård A, Hansen MR, Hallas J. Effect of an In-Hospital Multifaceted Clinical Pharmacist Intervention on the Risk of Readmission: A Randomized Clinical Trial. JAMA Intern Med. 2018 Mar 1;178(3):375-382. doi: 10.1001/jamainternmed.2017.8274. PMID: 29379953; PMCID: PMC5885912.
Home health patients	Zillich AJ, Snyder ME, Frail CK, et al. A randomized, controlled pragmatic trial of telephonic medication therapy management to reduce hospitalization in home health patients. Health Serv Res. 2014;49(5):1537-1554. doi:10.1111/1475-6773.12176.
Multimorbidity (≥2 chronic conditions) and/or polypharmacy (≥5 medications) criteria not met	Linn AJ, van Dijk L, van Weert JCM, Gebeyehu BG, van Bodegraven AA, Smit EG. Creating a synergy effect: A cluster randomized controlled trial testing the effect of a tailored multimedia intervention on patient outcomes. Patient Educ Couns. 2018 Aug;101(8):1419-1426. doi: 10.1016/j.pec.2018.03.017. Epub 2018 Mar 17. PMID: 29609899.
Multimorbidity (≥2 chronic conditions) and/or polypharmacy (≥5 medications) criteria not met	Cao XY, Tian L, Chen L, Jiang XL. Effects of a hospital-community partnership transitional program in patients with coronary heart disease in Chengdu, China: A randomized controlled trial. Jpn J Nurs Sci. 2017;14(4):320-331. doi:10.1111/jjns.12160.
Multimorbidity (≥2 chronic conditions) and/or polypharmacy (≥5 medications) criteria not met	Magny-Normilus C, Nolido NV, Borges JC, et al. Effects of an Intensive Discharge Intervention on Medication Adherence, Glycemic Control, and Readmission Rates in Patients With Type 2 Diabetes. J Patient Saf. 2021;17(2):73-80. doi:10.1097/PTS.0000000000000601.
Multimorbidity (≥2 chronic conditions) and/or polypharmacy (≥5 medications) criteria not met	Chen L, Sit JW, Shen X. Quasi-experimental evaluation of a home care model for patients with stroke in China. Disabil Rehabil. 2016; 38(23):2271-2276. doi:10.3109/09638288.2015.1123305.
Multimorbidity (≥2 chronic conditions) and/or polypharmacy (≥5 medications) criteria not met	Tu Q, Xiao LD, Ullah S, Fuller J, Du H. A transitional care intervention for hypertension control for older people with diabetes: A cluster randomized controlled trial. J Adv Nurs. 2020;76(10):2696-2708. doi:10.1111/jan.14466
**WRONG OUTCOME (n=1)**	
Physicians' adherence	Hohmann C, Neumann-Haefelin T, Klotz JM, Freidank A, Radziwill R. Providing systematic detailed information on medication upon hospital discharge as an important step towards improved transitional care. J Clin Pharm Ther. 2014;39(3):286-291. doi:10.1111/jcpt.12140
**WRONG SETTING (n=5)**	
Hospital Ward	Blum MR, Sallevelt BTGM, Spinewine A, O'Mahony D, Moutzouri E, Feller M, Baumgartner C, Roumet M, Jungo KT, Schwab N, Bretagne L, Beglinger S, Aubert CE, Wilting I, Thevelin S, Murphy K, Huibers CJA, Drenth-van Maanen AC, Boland B, Crowley E, Eichenberger A, Meulendijk M, Jennings E, Adam L, Roos MJ, Gleeson L, Shen Z, Marien S, Meinders AJ, Baretella O, Netzer S, de Montmollin M, Fournier A, Mouzon A, O'Mahony C, Aujesky D, Mavridis D, Byrne S, Jansen PAF, Schwenkglenks M, Spruit M, Dalleur O, Knol W, Trelle S, Rodondi N. Optimizing Therapy to Prevent Avoidable Hospital Admissions in Multimorbid Older Adults (OPERAM): cluster randomised controlled trial. BMJ. 2021 Jul 13;374:n1585. doi: 10.1136/bmj.n1585. Erratum in: BMJ. 2022 Dec 1; 379:o2859. doi: 10.1136/bmj.o2859. PMID: 34257088; PMCID: PMC8276068.
Hospital Ward	Löffler C, Drewelow E, Paschka SD, Frankenstein M, Eger J, Jatsch L, Reisinger EC, Hallauer JF, Drewelow B, Heidorn K, Schröder H, Wollny A, Kundt G, Schmidt C, Altiner A. Optimizing polypharmacy among elderly hospital patients with chronic diseases--study protocol of the cluster randomized controlled POLITE-RCT trial. Implement Sci. 2014 Oct 6;9:151. doi: 10.1186/s13012-014-0151-7. PMID: 25287853; PMCID: PMC4192341.
Hospital Ward	Nielsen TRH, Honoré PH, Rasmussen M, Andersen SE. Clinical Effects of a Pharmacist Intervention in Acute Wards - A Randomized Controlled Trial. Basic Clin Pharmacol Toxicol. 2017 Oct;121(4):325-333. doi: 10.1111/bcpt.12802. Epub 2017 Jun 19. PMID: 28457021.
Hospital Ward	Van der Linden L, Decoutere L, Walgraeve K, Milisen K, Flamaing J, Spriet I, Tournoy J. Combined Use of the Rationalization of Home Medication by an Adjusted STOPP in Older Patients (RASP) List and a Pharmacist-Led Medication Review in Very Old Inpatients: Impact on Quality of Prescribing and Clinical Outcome. Drugs Aging. 2017 Feb;34(2):123-133. doi: 10.1007/s40266-016-0424-8. PMID: 27915457.
Intermediate care facility	González-Bueno J, Sevilla-Sánchez D, Puigoriol-Juvanteny E, Molist-Brunet N, Codina-Jané C, Espaulella-Panicot J. Improving medication adherence and effective prescribing through a patient-centered prescription model in patients with multimorbidity. Eur J Clin Pharmacol. 2022 Jan;78(1):127-137. doi: 10.1007/s00228-021-03207-9. Epub 2021 Aug 27. PMID: 34448906.
**WRONG STUDY DESIGN (n=4)**	
Pilot study	Odeh M, Scullin C, Hogg A, Fleming G, Scott MG, McElnay JC. A novel approach to medicines optimisation post-discharge from hospital: pharmacist-led medicines optimisation clinic. Int J Clin Pharm. 2020 Aug;42(4):1036-1049.
Pilot study	Yoon J, Wu F, Chang E. Impact of primary care intensive management on medication adherence and adjustments. Am J Manag Care. 2020;26(8):e239-e245. Published 2020 Aug 1. doi:10.37765/ajmc.2020.44073.

REASON FOR EXCLUSION	EXCLUDED STUDIES
Retrospective RCT analysis	Von Buedingen F, Hammer MS, Meid AD, Müller WE, Gerlach FM, Muth C. Changes in prescribed medicines in older patients with multimorbidity and polypharmacy in general practice. BMC Fam Pract. 2018;19(1):131. Published 2018 Jul 28. doi:10.1186/s12875-018-0825-3.
Observational study	Leguelinel-Blache G, Dubois F, Bouvet S, et al. Improving Patient's Primary Medication Adherence: The Value of Pharmaceutical Counseling. Medicine (Baltimore). 2015;94(41):e1805. doi:10.1097/MD.0000000000001805.
**WRONG PUBLICATION TYPE (n=9)**	
Thesis	Zhao Y. Effects of a discharge planning intervention for elderly patients with coronary heart disease in Tianjin, China: A randomized controlled trial. Dissertation. Hong Kong Polytechnic University; 2004. Available from: ProQuest Dissertations & Theses (No. 3181588).
Abstract	Hastings S, Kembel L, Arain M, Ahmad A. A randomized controlled trial of a medication dispensing system to support individuals on multiple medications. Int J Integr Care. 2022;22(Suppl 1):14. Conference poster abstract.
RCT Protocol	Prados-Torres A, Del Cura-González I, Prados-Torres D, López-Rodríguez JA, Leiva-Fernández F, Calderón-Larrañaga A, López-Verde F, Gimeno-Feliu LA, Escortell-Mayor E, Pico-Soler V, Sanz-Cuesta T, Bujalance-Zafra MJ, Morey-Montalvo M, Boxó-Cifuentes JR, Poblador-Plou B, Fernández-Arquero JM, González-Rubio F, Ramiro-González MD, Coscollar-Santaliestra C, Martín-Fernández J, Barnestein-Fonseca MP, Valderas-Martínez JM, Marengoni A, Muth C; Multi-PAP Group. Effectiveness of an intervention for improving drug prescription in primary care patients with multimorbidity and polypharmacy: study protocol of a cluster randomized clinical trial (Multi-PAP project). Implement Sci. 2017 Apr 27;12(1):54.
	Ahmad A, Hugtenburg J, Welschen LM, Dekker JM, Nijpels G. Effect of medication review and cognitive behaviour treatment by community pharmacists of patients discharged from the hospital on drug related problems and compliance: design of a randomized controlled trial. BMC Public Health. 2010 Mar 15;10:133. doi: 10.1186/1471-2458-10-133. PMID: 20230611; PMCID: PMC2850341.
	McCarthy C, Clyne B, Corrigan D, et al. Supporting prescribing in older people with multimorbidity and significant polypharmacy in primary care (SPPiRE): a cluster randomised controlled trial protocol and pilot. Implement Sci. 2017;12(1):99. Published 2017 Aug 1. doi:10.1186/s13012-017-0629-1.
	Kouladjian O'Donnell L, Sawan M, Reeve E, Gnjidic D, Chen TF, Kelly PJ, Bell JS, Hilmer SN. Implementation of the Goal-directed Medication review Electronic Decision Support System (G-MEDSS)© into home medicines review: a protocol for a cluster-randomised clinical trial in older adults. BMC Geriatr. 2020 Feb 12;20(1):51.
	Chrischilles EA. Personal Health Records and Elder Medication Use Quality - Final Report. (Prepared by the University of Iowa under Grant No. R18 HS017034). Rockville, MD: Agency for Healthcare Research and Quality, 2012.
	Bernal DD, Stafford L, Bereznicki LR, Castelino RL, Davidson PM, Peterson GM. Home medicines reviews following acute coronary syndrome: study protocol for a randomized controlled trial. Trials. 2012;13:30. Published 2012 Apr 2. doi:10.1186/1745-6215-13-30.
	Haramiova Z, Stasko M, Hulin M, Tesar T, Kuzelova M, Morisky DM. The effectiveness of daily SMS reminders in pharmaceutical care of older adults on improving patients' adherence to antihypertensive medication (SPPA): study protocol for a randomized controlled trial. Trials. 2017;18(1):334. Published 2017 Jul 18. doi:10.1186/s13063-017-2063-8.
**RCT RESULTS IDENTIFIED BY REFERENCES (n=6):**	
Muth C, Uhlmann L, Haefeli WE, et al. Effectiveness of a complex intervention on Prioritising Multimedication in Multimorbidity (PRIMUM) in primary care: results of a pragmatic cluster randomised controlled trial. BMJ Open. 2018;8(2):e017740. Published 2018 Feb 24. doi:10.1136/bmjopen-2017-017740.	
Del Cura-González I, López-Rodríguez JA, Leiva-Fernández F, Gimeno-Miguel A, Poblador-Plou B, López-Verde F, Lozano-Hernández C, Pico-Soler V, Bujalance-Zafra MJ, Gimeno-Feliu LA, Aza-Pascual-Salcedo M, Rogero-Blanco M, González-Rubio F, García-de-Blas F, Polentinos-Castro E, Sanz-Cuesta T, Castillo-Jimena M, Alonso-García M, Calderón-Larrañaga A, Valderas JM, Marengoni A, Muth C, Prados-Torres JD, Prados-Torres A; Multi-Pap Group. How to Improve Healthcare for Patients with Multimorbidity and Polypharmacy in Primary Care: A Pragmatic Cluster-Randomized Clinical Trial of the MULTIPAP Intervention. J Pers Med. 2022 May 6;12(5):752.	
Ahmad A, Nijpels G, Dekker JM, Kostense PJ, Hugtenburg JG. Effect of a pharmacist medication review in elderly patients discharged from the hospital. Arch Intern Med. 2012;172(17):1346-1347. doi:10.1001/archinternmed.2012.2816.	
McCarthy C, Clyne B, Boland F, et al. GP-delivered medication review of polypharmacy, deprescribing, and patient priorities in older people with multimorbidity in Irish primary care (SPPiRE Study): A cluster randomised controlled trial. PLoS Med. 2022;19(1):e1003862. Published 2022 Jan 5. doi:10.1371/journal.pmed.1003862.	
Kouladjian O'Donnell L, Gnjidic D, Sawan M, Reeve E, Kelly PJ, Chen TF, Bell JS, Hilmer SN. Impact of the Goal-directed Medication Review Electronic Decision Support System on Drug Burden Index: A cluster-randomised clinical trial in primary care. Br J Clin Pharmacol. 2021 Mar;87(3):1499-1511.	
Chrischilles EA, Hourcade JP, Doucette W, Eichmann D, Gryzlak B, Lorentzen R, Wright K, Letuchy E, Mueller M, Farris K, Levy B. Personal health records: a randomized trial of effects on elder medication safety. J Am Med Inform Assoc. 2014 Jul-Aug;21(4):679-86. doi: 10.1136/amiajnl-2013-002284. Epub 2013 Dec 10. PMID: 24326536; PMCID: PMC4078278.	
**IDENTIFIED BY HANDSEARCHING (n=8):**	
Bernsten C, Björkman I, Caramona M, et al. Improving the well-being of elderly patients via community pharmacy-based provision of pharmaceutical care: a multicentre study in seven European countries. Drugs Aging. 2001;18(1):63-77. doi:10.2165/00002512-200118010-00005.	
Holland R, Lenaghan E, Harvey I et al. Does home based medication review keep older people out of hospital? The HOMER randomised controlled trial. BMJ 2005; 330: 293 – 8.	
Nazareth I, Burton A, Shulman S, Smith P, Haines A, Timberal H. A pharmacy discharge plan for hospitalized elderly patients--a randomized controlled trial. Age Ageing. 2001;30(1):33-40. doi: 10.1093/ageing/30.1.33.	
Bolas H, Brookes K, Scott M, McElnay J. Evaluation of a hospital-based community liaison pharmacy service in Northern Ireland. Pharm World Sci. 2004;26(2):114-120. doi:10.1023/b:phar.0000018601.11248.89.	
Al-Rashed SA, Wright DJ, Roebuck N, Sunter W, Chrystyn H. The value of inpatient pharmaceutical counselling to elderly patients prior to discharge. Br J Clin Pharmacol. 2002 Dec;54(6):657-64. doi: 10.1046/j.1365-2125.2002.01707.x. PMID: 12492615; PMCID: PMC1874498.	
Moral, R.R.; Torres, L.A.; Ortega, L.P.; Larumbe, M.C.; Villalobos, A.R.; García, J.A.; Rejano, J.M. Effectiveness of motivational interviewing to improve therapeutic adherence in patients over 65 years old with chronic diseases: A cluster randomized clinical trial in primary care. Patient Educ. Couns. 2015, 98, 977–983.	
Morales Suárez-Varela, M.T. Estudio sobre la utilidad del pastillero para mejorar el cumplimiento terapéutico. Atención Primaria 2009, 41, 185–191.	
Biswas, A.; Sinha, N.; Ray, K.; Tripathi, S.K. A study on drug use and medication management perspectives among elderly and the impact of professional oversight. J. Clin. Diagn. Res. 2018, 12, FC1–FC7.	
