# Supplementary material for: Interventions to enhance in-home taking medication among older adults with multimorbidity/polypharmacy: a systematic review and meta-analysis
Source: Front Public Health. 2026 Jan 28;13:1701622. doi: 10.3389/fpubh.2025.1701622 (PMC12891206; doi:10.3389/fpubh.2025.1701622)
Supplement: Supplementary file 1 [file Data_Sheet_1.zip › Supplementary Table 4a-4b.Characteristics of included studies.pdf]

SUPPLEMENTARY TABLE 4a. RCTs								
Author/ Year	Study design	Country/ Setting	Sample (n)	Intervention group characteristics	Control group characteristics	Intervention provider	Intervention type	Follow-up period (months)
				Frequency (percentage), mean ± SD/ (range) or median [IQR], mean (95% CI)				
Aguiar et al. 2018	RCT	Brazil/ Secondary care clinic	I: 40<br>C: 40	• Age : 61.1 ± 7.9 years<br>• Female gender: 69.4% women<br>• Comorbidities:<br>Hypertension: 86.1%<br>Dyslipidemia: 88.9%<br>• Number of medications: 7 [5.8-9]	• Age: 62.4 ± 8.2 years<br>• Female gender: 64.9% women<br>• Comorbidities:<br>Hypertension: 89.2%<br>Dyslipidemia: 89.2%<br>• Number of medications: 7 [6-8]	Pharmacist-Physician	-Education<br>-Medication review<br>-Face-to-face pharmaceutical consultations<br>-Follow-up: telephone<br>-Support tool: educational leaflets	12
Ahmad et al. 2012; Van der Heijden et al. 2019	Cluster- RCT<br><br>Unit of randomisation: pharmacy	Netherlands/ Community Pharmacy	I: 180<br>C: 160	• Age: 75.5 ± 9.2 years<br>• Female gender: 48.1% women<br>• Comorbidities: 2.7 ± 1.4<br>• Number of medications: 8.9 ± 2.7<br>• Educational level:<br>Low: 33.3%<br>Medium: 45.7%<br>High: 21.0%	• Age: 73.9 ± 8.3 years<br>• Female gender: 56.4% women<br>• Comorbidities: 3.3 ± 1.7<br>• Number of medications: 8.4 ± 3.0<br>• Educational level:<br>Low: 31.2%<br>Medium: 51.6 %<br>High: 17.2 %	Community pharmacist	-Medication review<br>-Counseling, Education, Motivational Interviewing<br>-Follow-up: face-to-face (home/pharmacy)<br>-Support tool: written outlines of drug regimens	12
Bernsten et al. 2001	RCT<br><br>Unit of randomisation: pharmacy	Denmark, Germany, Netherlands, Northern Ireland, Republic of Ireland, Portugal, Sweden/ Community Pharmacy	I: 1290<br>C: 1164	• Age : 74 [8] years<br>• Female gender: 57.9% women<br>• Number of medications: 7.1 ± 2.5<br>• Patients living alone: 37.2%<br>• Patients requiring help with daily activities: 50.9%	• Age : 74 [8] years<br>• Female gender: 57.3% women<br>• Number of medications: 7.0 ± 2.5<br>• Patients living alone: 37.7%<br>• Patients requiring help with daily activities: 47.4%	Community pharmacist	-Education<br>-Medication Review<br>-Deprescribing<br>-Support tool: drug-reminder charts	18
Biswas et al. 2018	RCT	India/ Community (Home-Community dispensary)	I: 40<br>C: 35	• Age : 68.4 ± 5.77 years<br>• Female gender: 22.5%<br>• Number of medications: 3.27<br>• Marital status:<br>Married: 70%<br>Others: 30%<br>• Number of family members: 2.93±1.83<br>• Educational status:<br>Illiterate:17.50%<br>Read and write: 27.50%<br>Up to primary: 15.00%<br>Primary to High school: 7.50%<br>University: 32.50%	• Age : 66.89 ± 6.77 years<br>• Female gender: 45.70%<br>• Number of medications: 3.05<br>• Marital status:<br>Married: 68.57%<br>Others: 31.43%<br>• Number of family members:3.74± 1.73<br>• Educational status:<br>Illiterate: 22.86%<br>Read and write: 28.57%<br>Up to primary: 8.57%<br>Primary to High school: 14.28%<br>University: 25.71%	Clinical pharmacist	-Counselling<br>-Support tools: time table charting<br>-Follow-up: face-to-face/ telephone	12
Bolas et al. 2004	RCT	Ireland/ Hospital discharge	I: 119<br>C: 124	• Age: 73 (1–27) years<br>• Female gender: 50.62% women<br>• Number of medications: 6.79 (2–16) at discharge<br>• Living alone: 33.30%<br>• Living with spouse: 48.15%<br>• Living with family: 18.52%	• Age: 75 (1–37) years<br>• Female gender: 51.85% women<br>• Number of medications: 6.73 (2-16) at discharge<br>• Living alone: 41.98%<br>• Living with spouse: 44.40%<br>• Living with family: 13.58%	Community liaison pharmacist	-Medication Reconciliation<br>-Medication Review<br>-Couselling<br>-Discharge coordination<br>-Support tool: personalized medication record sheet /helpline card	3
Briggs et al. 2015	RCT	Ausatralia/ Emergency Department	I: 525<br>C: 496	• Age: 82 ± 6 years<br>• ISAR score[1]: 3 ± 1	• Age: 81 ± 6 years<br>• ISAR score[1]: 3 ± 1	Clinical pharmacist	-Medication review<br>-Collaborative care	4
Campins et al. 2017	RCT	Spain/ Primary Care	I: 252<br>C: 251	• Age: 79.16±5.50 years<br>• Female gender: 60.3% women<br>• Number of medications: 10.79 ± 2.52<br>• Educational level:<br>No formal schooling: 41.0%<br>Primary education:44.2%<br>Secondary education: 11.6%<br>University: 3.2%	• Age: 78.78±5.46 years<br>• Female gender: 57.4% women<br>• Number of medications: 10.91 ± 2.65<br>• Educational level:<br>No formal schooling: 42.3%<br>Primary education: 46.4%<br>Secondary education:8.9%<br>University: 2.4%	Clinical pharmacist-Physician	-Medication Review<br>-Shared Decision-Making	12
Chrischilles et al. 2014	RCT	United States/ Home-based	I: 802<br>C: 273	• Age: 72.5 ± 6.0 years<br>• Female gender: 57.5% women<br>• Comorbidities: 3.6 ± 2.3<br>• Educational level:<br>Some high school or less: 1.8%<br>High school diploma: 23.2%<br>Technical or trade school: 34.6%<br>Bachelor's degree: 23.0%<br>Master's degree or higher: 17.4%	• Age:72.0 ± 6.3 years<br>• Female gender: 54.9% women<br>• Comorbidities: 3.6 ± 2.2<br>• Educational level:<br>Some high school or less: 0.4%<br>High school diploma: 28.3%<br>Technical or trade school: 32.4%<br>Bachelor's degree: 20.2%<br>Master's degree or higher: 18.8%	Electronic tool	-Personal Health Record<br>-Web-based application<br>-Education<br>-Follow-up: email/telephone	6

SUPPLEMENTARY TABLE 4a. RCTs								
Author/ Year	Study design	Country/ Setting	Sample (n)	Intervention group characteristics	Control group characteristics	Intervention provider	Intervention type	Follow-up period (months)
				Frequency (percentage), mean ± SD/ (range) or median [IQR], mean (95% CI)				
Del Cura-González et al. 2022	Cluster- RCT<br><br>Unit of randomisation: GP practices	Spain/ Primary Care	I: 298<br>C: 295	• Age: 69.6 ± 2.7 years<br>• Female gender: 61.0%<br>• Comorbidities: 5.0 (4.0, 7.0)<br>• Number of medications: 7.0 (6.0, 9.0)<br>• Education level:<br>Did not complete primary studies: 46%<br>Completed primary studies: 30.9%<br>Bachelor or higher:23.2%<br>• Marital status:<br>Single: 3.7%<br>Married: 74.8%<br>Separated: 5.4%<br>Widower: 16.1%	• Age: 69.9 ± 2.7 years<br>• Female gender: 70.7%<br>• Comorbidities: 5.0 (4.0, 6.0)<br>• Number of medications: 7.0 (5.0, 8.0)<br>• Education level:<br>Did not complete primary studies: 48.1%<br>Completed primary studies: 35.3%<br>Bachelor or higher:16.6%<br>• Marital status:<br>Single: 4.1%<br>Married: 75.9%<br>Separated: 4.4%<br>Widower: 15.6%	Physician- Nurse	-Medication review<br>-Shared-decision making<br>-Support tool: printed copy of the plan<br>-Follow-up: face-to-face	12
Geurts et al. 2016	RCT	Netherlands/Primary Care	I: 248<br>C: 264	Intervention patients with intervention<br>• Age: 72.5 ± 7.74 years<br>• Female gender: 53.9% women<br>• Number of medications: 8.3 ± 2.72<br><br>Intervention patients without intervention:<br>• Age: 71.8 ± 8.37 years<br>• Female gender: 47.1% women<br>• Number of medications: 8.0 ± 3.28	• Age: 73.1 ± 7.80 years<br>• Female gender: 52.7% women<br>• Number of medications: 7.9 ± 2.93	Community pharmacist	-Medication review<br>-Personalized care planning<br>-Shared-decision making<br>-Web-based application	12
Heaton et al. 2019	RCT	United States/ Hospital discharge	I: 213<br>C: 187	• Age: 60.3 ± 12.9 years<br>• Female gender: 51.6 %<br>• Number of medications: 14.9 ± 7.5<br>• Marital status:<br>Married 16.4%<br>Not married 17.4%<br>Unknown 66.2%	• Age: 63.2 ± 12.3 years<br>• Female gender: 42.2%<br>• Number of medications: 14.9 ± 6.8<br>• Marital status:<br>Married 12.8%<br>Not married 16.0%<br>Unknown 71.1%	Community Pharmacist	-Motivational Interviewing<br>-Medication Review<br>-Reconciliation<br>-Education<br>-Counselling<br>-Support tool: pillboxes/ personal medication list/medication action plan<br>-Follow-up: face-to-face/ telephone	6
Herrinton et al. 2023	RCT	United States / Primary Care	I: 1237<br>C: 1233	• Age:<br>76-79 years: 41%<br>80-84 years: 32.25%<br>≥85 years: 26.7%<br>• Female gender: 52.1% women<br>• Number of medications: 13.6 (95% CI: 13.4-13.8)	• Age:<br>76-79 years: 42.5%<br>80-84 years: 35%<br>≥85 years: 22.5%<br>• Female gender: 50.9% women<br>• Number of medications: 13.6 (95% CI: 13.4-13.8)	Community pharmacist-Physician	- Medication Review<br>-Deprescribing<br>-Shared decision-making<br>-Follow-up: telephone/ video encounters	12
Holland et al. 2005	RCT	United Kingdom/ Home-based	I: 437<br>C: 435	• Age: 85.4 ± 4.0 years<br>• Female gender: 61.1% women<br>• Number of medications: 6.0 ± 2.7<br>• Living alone: 61.3%<br>• Monitored dose system: 19.8%<br>• Disease status:<br>Cardiovascular: 31.2%<br>Muskuloeskeletal: 14.2%<br>Gastrointestinal:11.0%<br>Respiratory: 11.2%<br>Neurological: 9.3%<br>Senility/Dementia: 3.7%<br>Genitourinary: 4.0%<br>Cancer: 3.5%<br>Other: 15.6%	• Age: 85.5 ± 4.0 years<br>• Female gender: 63.8% women<br>• Number of medications: 5.8 ± 2.3<br>• Living alone: 62.9%<br>• Monitored dose system: 17.4%<br>• Disease status:<br>Cardiovascular: 33.8%<br>Muskuloeskeletal: 15.3%<br>Gastrointestinal:12.7%<br>Respiratory: 11.5%<br>Neurological: 5.9%<br>Senility/Dementia: 1.4%<br>Genitourinary: 3.8%<br>Cancer: 1.6%<br>Other: 15.5%	Pharmacist	-Medication review<br>-Education<br>-Follow-up: home visits<br>-Support tool: compliance aid	6
Insel et al. 2012	RCT	United States/ Home-based	I: 63<br>C: 59	• Age: 76.8 ± 7.4 years<br>• Female gender: 77.7% women<br>• Number of medications: 4.6 ± 3.8 medicines<br>• Education level:<br>< High school: 14.28%<br>High school:14.28%<br>Post-high school:28.60%<br>College degree: 42.86%	• Age: 77.2 ± 7.6 years<br>• Female gender: 74.6% women<br>• Number of medications: 8.6 ± 5.4<br>• Education level:<br>< High school: 3.40%<br>High school: 13.56%<br>Post-high school: 27.12%<br>College degree: 55.93%	Nurse	-Memory strategies<br>-Education<br>-Support tool: pill organizer<br>-Follow-up: home visits	6

SUPPLEMENTARY TABLE 4a. RCTs								
Author/ Year	Study design	Country/ Setting	Sample (n)	Intervention group characteristics	Control group characteristics	Intervention provider	Intervention type	Follow-up period (months)
				Frequency (percentage), mean ± SD/ (range) or median [IQR], mean (95% CI)				
Jarab et al. 2012	RCT	Jordania/ Secondary care (Outpatient COPD specialist clinic)	I: 66<br>C: 67	•Age: 61 [14] years<br>•Female gender: 60.6%<br>•Comorbidities: 53.0% had ≥1 comorbid condition<br>•Number of medications: 8 [5]<br>•Education level:<br>High (University) 10.6%<br>Low 89.4%<br>•Marital status:<br>Married 84.8%<br>Other 15.2%<br>•Living arrangements:<br>Alone 77.3%<br>Not alone 22.7%	•Age: 64 years [15] years<br>•Female gender: 58.2%<br>•Comorbidities: 55.2% had ≥1 comorbid condition<br>•Number of medications: 8 [5]<br>•Education level:<br>High (University) 9.0%<br>Low 91.0%<br>•Marital status:<br>Married 82.1%<br>Other 17.9%<br>Living arrangements:<br>Alone 80.6%<br>Not alone 19.4%	Pharmacist	-Medication review<br>-Motivational interviewing<br>-Education: structured education including medication adherence component<br>-Support tool: educational booklet (partly addressing medication adherence)<br>-Follow-up: face-to-face visit	6
Jerant et al. 2009	RCT	United States / Primary Care	I: 139<br>C: 138	Telephone participants:<br>• Age: 61.2 ± 11.6 years<br>• Female gender: 78.0% women<br>• Comorbidities:<br>1:51.0%<br>2: 29.0%<br>3: 15.0%<br>>4: 4.0%<br>• Education level:<br>High school: 14.0%<br>Some college: 36.0%<br>College graduate or greater: 47.0%<br>Declined to answer: 5.0%<br>• Marital status: 57.0% married	• Age: 60.1 ± 11.7 years<br>• Female gender: 75.0% women<br>• Comorbidities:<br>1:31.0%<br>2: 47.0%<br>3: 15.0%<br>>4: 7.0%<br>• Education level:<br>High school: 16.0%<br>Some college: 42.0%<br>College graduate or greater: 41.0%<br>Declined to answer: 1.0%<br>• Marital status: 55.0% married	Nurse	-Education<br>-Self-management skills<br>-Follow up: telephone/Home-visits	12
Köberlein-Neu et al. 2016	Cluster-RCT (stepped wedge design)<br><br>Unit of randomisation: physician	Germany/ Primary Care	Cohort 1: 66<br>Cohort 2: 49<br>Cohort 3: 47	Cohort 1: (start intervention after end of recruitment period, n= 59 patients)<br>• Age: 76.4 ± 6.1 years<br>• Female gender: 48.75% women<br>• Marital status:<br>Single: 3.4 %<br>Married: 64.4%<br>Divorced: 1.7%<br>Widowed: 30.5%<br>• Comorbidities: 12.61 ± 6.1<br>• Number of medications: 10.3 ± 3.6	Cohort 2: (start intervention after 3 months, n= 40 patients)<br>• Age: 78.5 ± 6.2 years<br>• Female gender: 75.0% women<br>• Comorbidities: 12.3 ± 4.6<br>• Number of medications: 9.0 ± 2.8<br>• Marital status:<br>SIngle: 5.9 %<br>Married: 35.3 %<br>Divorced: 14.7 %<br>Widowed: 44.1 %<br><br>Cohort 3: (start intervention after 6 months, n= 43 patients)<br>• Age:75.5 ± 5.4 years<br>• Female gender: 58.1 % women<br>• Comorbidities: 13.2 ± 6.3<br>• Number of medications: 8.8 ± 2.4<br>• Marital status:<br>Single: 0.0%<br>Married: 74.4%<br>Divorced: 4.6%<br>Widowed: 21%	Pharmacist-Physician	-Medication Review<br>-Follow-up: home visits	6
Kouladjian O'Donnell et al. 2021	Cluster-RCT<br><br>Unit of Randomization: Clinical pharmacists	Australia/ Primary Care	I: 88<br>C: 113	• Age: 77.9 ± 8.0 years<br>• Female gender: 67.0%<br>• Number of medications: 10.9 ± 3.8<br>• Education level:<br>Incomplete secondary education:60.0%<br>Secondary education completed: 18.0%<br>Tertiary education completed: 22.0%<br>• Marital status:<br>Never married/single: 8.0%<br>Widowed: 41.0%<br>Divorced/separated: 14.0%<br>Married/de-facto: 37.0%	• Age: 78.6 ± 7.3 years<br>• Female gender: 55.0%<br>• Number of medications: 10.5 ± 3.6<br>• Education level:<br>Incomplete secondary education 71.0%<br>Secondary education completed 15.0%<br>Tertiary education completed 15.0%<br>• Marital status:<br>Never married/single: 4.0%<br>Widowed: 37.0%<br>Divorced/separated: 9.0%<br>Married/de-facto: 50.0%	Clinical Pharmacist	-Medication review<br>-Deprescribing<br>-Shared-decision making<br>-Support Tool: electronic decision support system	3

SUPPLEMENTARY TABLE 4a. RCTs								
Author/ Year	Study design	Country/ Setting	Sample (n)	Intervention group characteristics	Control group characteristics	Intervention provider	Intervention type	Follow-up period (months)
				Frequency (percentage), mean ± SD/ (range) or median [IQR], mean (95% CI)				
Lee et al. 2006	RCT	United States/ Secondary Care (Military Medical Center)	I: 83<br>C:76	• Age: 77 ± 10.5 years<br>• Female gender: 25.3%<br>• Number of medications: 9.1 ± 3.2<br>• Education level:<br>< High school: 3.7%<br>High school graduate: 32.1%<br>Some college: 39.5%<br>College graduate: 24.7%<br>Unknown: 2.4%	• Age: 78 ± 6.2 years<br>• Female gender: 26.3%<br>• Number of medications: 8.3 ± 2.8<br>• Education level:<br>< High school: 12.9%<br>High school graduate: 38.6%<br>Some college: 30.0%<br>College graduate: 18.6%<br>Unknown: 5.5%	Clinical pharmacist	-Education<br>-Support tool: customized blister packs.<br>-Follow-up: face-to-face	6
Lembeck et al. 2019	RCT	Denmark/ Hospital discharge	I: 270<br>C: 267	• Age: 82.5 ± 7.6 years<br>• Female gender: 56% women<br>• Charlson comorbidity score²:<br>0 : 13%, 1: 20% 2: 24%, 3: 16% , 4–11: 26%<br>• Marital status:<br>Married 28%<br>Divorced 12%<br>Unmarried 6%<br>Widowed 53%	• Age: 82.2 ± 7.3 years<br>• Female gender: 64% women<br>• Charlson comorbidity score²:<br>0 : 16%, 1 : 26%, 2 : 19%, 3: 14%, 4–11: 24%<br>• Marital status:<br>Married 29%<br>Divorced 11%<br>Unmarried 6%<br>Widowed 55%	Project Nurses-Municipal Nurses	-Structured discharge planning<br>-Follow-up: home visit	6
Lenaghan et al. 2007	RCT	United Kingdom/ Primary care	I: 69<br>C: 67	• Age: 84.5 years<br>• Female gender: 67.6% women<br>• Number of medications: 7.1<br>• Living alone: 64.7%<br>• Social class (I,II, IIInm): 48.5%	• Age: 84.1<br>• Female gender: 63.6% women<br>• Number of medications: 7.7<br>• Living alone: 65.1%<br>• Social class (I,II, IIInm): 43.9%	Community pharmacist	-Medication Review (Home)<br>-Education<br>-Follow-up: Face-to-face visits	6
Lenander et al. 2014	RCT	Sweden/ Primary Care	I: 107<br>C: 102	• Age: 79.0 years (95% CI: 77.8-80.2)<br>• Female gender: 65.4% women<br>• Number of medications: 8.5 (95% CI: 7.9-9.1)<br>• Comorbidities: 5.1 (95% CI: 4.7-5.4)	• Age: 79.7 years (96% CI: 78.4, 81.1)<br>• Female gender: 68.6% women<br>• Number of medications: 7.4 (95% CI: 6.9, 8.0)<br>• Comorbidities: 4.5 (95% CI: 4.2, 4.9)	Clinical pharmacist	-Medication review<br>-Counselling<br>-Shared-Decision making	12
Martínez-Mardones et al. 2023	Cluster-RCT<br><br>Unit of randomisation: GP practices	Chile/ Primary Care	I: 174<br>C: 150	• Age: 73.2 ± 5.82 years<br>• Female gender: 72.5% women<br>• Comorbidities: 4.17± 1.39<br>• Number of medications: 8.31 ± 2.48<br>• Educational level:<br>No studies: 45.4%<br>Primary: 32.8%<br>Secondary : 19.0%<br>Tertiary: 2.8%<br>• Marital status:<br>With partner: 59.2%<br>Without partner: 40.8%	• Age: 74.1 ± 5.99 years<br>• Female gender: 71.4% women<br>• Comorbidities: 4.17 ± 1.39<br>• Number of medications: 7.86 ± 2.27<br>• Educational level:<br>No studies: 47.4%<br>Primary: 38.0%<br>Secondary : 13.3 %<br>Tertiary: 1.3 %<br>• Marital status:<br>With partner: 57.3%<br>Without partner: 42.7%	Primary Care Pharmacist	-Medication Review<br>-Patient Education<br>-Follow-up: face-to-face visits	12
McCarthy et al. 2022	Cluster-RCT<br><br>Unit of randomisation: GP practices	Ireland/ Primary Care	I: 208<br>C: 196	• Age: 76.67 ± 6.80 years<br>• Female gender: 57.21% women<br>• Number of medications: 16.02 ± 3.93<br>• Education:<br>No schooling: 0.00%<br>Primary school: 33.17%<br>Some secondary education: 25.48%<br>Complete secondary education: 19.23%<br>Some third level education: 9.62%<br>Complete third level education: 7.69%<br>Unknown: 4.81%	• Age: 76.33 ± 3.89 years<br>• Female gender: 57.14% women<br>• Number of medications:<br>17.55 ± 4.10<br>• Education:<br>No schooling: 1.53%<br>Primary school: 43.88%<br>Some secondary education: 22.45%<br>Complete secondary education: 10.20%<br>Some third level education: 9.69%<br>Some third level education: 6.63%<br>Unknown: 5.61%	General Practitioner	-Medication Review<br>-Deprescribing<br>-Shared-Decision Making	6
Messerli et al. 2016	RCT	Switzerland/ Community Pharmacy	I: 218<br>C: 232	• Age: 67.2 ± 11.52 years<br>• Female gender: 54.1% women<br>• Number of medications: 6.8 ± 2.92<br>Living alone: 36.5%<br>Used a weekly dosing aid: 31.5%	• Age: 67.1 ± 11.56 years<br>• Female gender: 53.9% women<br>• Number of medications:<br>not available<br>• Living alone: 31.9 %<br>• Use of weekly dosing aid: not available	Community Pharmacist	-Medication review<br>-Counselling<br>-Education<br>-Collaborative care<br>-Support tool: medication plan<br>-Follow-up: face-to-face visits/ Telephone	7

SUPPLEMENTARY TABLE 4a. RCTs								
Author/ Year	Study design	Country/ Setting	Sample (n)	Intervention group characteristics	Control group characteristics	Intervention provider	Intervention type	Follow-up period (months)
				Frequency (percentage), mean ± SD/ (range) or median [IQR], mean (95% CI)				
Morales Suárez-Varela et al. 2009	RCT	Spain/ Home-based	I: 89<br>C: 93	• Age : 77.08 years<br>• Female gender: 71.9%<br>• Comorbidities:<br>Diabetes: 44.9%<br>Hypertension: 85.4%<br>COPD: 18.0%<br>Coronary Heart disease: 39.3%<br>• Number of medications: 8.35<br>• Self-managed medication: 29.2 %<br>• Educational level:<br>Iliterate: 76.4%<br>Middle: 23.6%	• Age : 77.39 years<br>• Female gender: 68.8%<br>• Comorbidities:<br>Diabetes: 41.9%<br>Hypertension: 83.9%<br>COPD: 20.4%<br>Coronary Heart disease: 43.0%<br>• Number of medications: 7.83<br>• Self-managed medication: 37.6 %<br>• Educational level:<br>Iliterate: 77.4%<br>Middle: 22.6%	Nurse	-Education<br>-Support tool: Medication package Pillbox<br>-Follow-up: telephone/ home-vistis	2
Muth et al. 2018	Cluster-RCT<br>Unit of randomisation: GP practices	Germany/ Primary Care	I: 252<br>C: 253	• Age: 72.5±6.5 years,<br>• Female gender: 53.0% women<br>• Number of comorbidities:<br>CIRS[3] number of affected organ systems 4.6 ± 2.4<br>• Patients living with spouse: 61.0%<br>• Patients fending for themselves: 94.0 %<br>• Educational level:<br>High: 6.0%<br>Middle: 27.0%<br>Low: 68.0%	• Age: 71.7±7.4 years<br>• Female gender: 52.0% women<br>• Number of comorbidities:<br>CIRS[3] number of affected organ systems 4.4 ± 2.3<br>• Patients living with spouse: 67.0%<br>• Patients fending for themselves: 94.0 %<br>• Educational level:<br>High: 10.0%<br>Middle: 32.0%<br>Low: 58.0%	General Practitioner	-Medication Review<br>-Medication Reconciliation<br>-Support tool: Computerised Decision Support System<br>-Follow-up: telephone/face-to-face	9
Nazareth et al. 2001	RCT	United Kingdom/ Hospital discharge	I: 181<br>C: 181	• Age: 84 ±5.2 years<br>• Female gender: 62.0% women<br>• Comorbidities: 3<br>• Number of medications: 6 ± 2<br>• Social status:<br>63.0% social class III<br>11.0% social class II	• Age: 84± 5.4 years<br>• Female gender: 66.0% women<br>• Comorbidities: 3<br>• Number of medications: 6 ± 2<br>• Social status:<br>57.0% social class III<br>9.0% social class II	Clinical-Community pharmacists	-Pharmacy discharge plan<br>-Medication Review<br>-Counselling<br>-Follow-up: home visit<br>-Support tool: medication plan	6
Olesen et al. 2014	RCT	Denmark/ Home-based	I: 315<br>C: 315	• Age: 74 [70-80] years<br>• Female gender: 53.0% women<br>• Number of medications: 7 [5-8]	• Age: 74 [70-80] years<br>• Female gender: 51.0 % women<br>• Number of medications: 7 [5-8]	Community Pharmacist	-Medication review<br>-Deprescribing<br>-Education<br>-Support tool: information leaflets<br>-Follow-up: telephone	24
Poorcheraghi et al. 2023	RCT	Iran/ Secondary care (Geriatric Hospital)	I: 96<br>C: 96	• Age: 68.9 ± 5.2 years<br>• Female gender: 48.96% women<br>• Disease status:<br>Cardiovascular disease: 56.25%<br>Hypertension: 66.67%<br>Diabetes: 48.96%<br>COPD: 48.96%<br>• Number of medications: 6.67±2.4<br>• Education level:<br>Primary school: 7.2%<br>Junior school: 15.6%<br>High school: 28.1%<br>Diploma: 26.2%<br>Academic education: 22.9%<br>• Dependent: 13.54%	• Age: 69 ± 5.6 years<br>• Female gender: 44.8% women<br>• Disease status:<br>Cardiovascular disease:53.12%<br>Hypertension: 46.58%<br>Diabetes: 57.29%<br>COPD: 39.58%<br>• Number of medications: 6.53±2.3<br>• Education level:<br>Primary school: 11.46%<br>Junior school: 16.67%<br>High school: 28.12%<br>Diploma: 30.21%<br>Academic education: 13.54%<br>• Dependent: 11.46%	Electronic tool	-Mobile application<br>-Reminders<br>-Education and counselling<br>-Follow-up: telephone/ face-to-face	2
Sáez de la Fuente et al. 2011	RCT	Spain/ Hospital discharge	I: 29<br>C: 30	• Age: 73[28-93] years<br>• Female gender: 34.5% women<br>• Number of medications: 8.3 [7.4-9.3]<br>• 69% caregivers at discharge and 57.7% at telephone interview (85% family caregiver)<br>• Education level:<br>Iliterate: 17.2%<br>Primary studies: 41.4%<br>Secondary/ university education: 41.4%<br>• Barthel Index at discharge: 80 (80-100)	• Age: 75 [14-96] years<br>• Female gender: 36.7% women<br>• Number of medications: 7.6 [6.5-8.7]<br>• 60% caregivers at dicharge and 70.8% at telephone interview (88.9% family caregiver)<br>• Education level:<br>Iliterate: 26.7%<br>Primary studies: 56.7%<br>Secondary/ university education: 16.7%<br>• Barthel Index at discharge: 100 (70-100)	Clinical Pharmacist	-Education	1-1.6

SUPPLEMENTARY TABLE 4a. RCTs								
**Author/ Year**	**Study design**	**Country/ Setting**	**Sample (n)**	**Intervention group characteristics**	**Control group characteristics**	**Intervention provider**	**Intervention type**	**Follow-up period (months)**
				**Frequency (percentage), mean ± SD/ (range) or median [IQR], mean (95% CI)**				
Sánchez Ulayar et al. 2011	RCT	Spain/ Hospital Discharge	I: 50<br>C: 50	• Age: 75 ± 11 years<br>• Female gender: 42% women<br>• Number of medications: 9.7 ± 2.9<br>• 20% used Personalised Dosage System (PDS) at discharge<br>• 46% caregiver at interview	• Age: 75 ± 11 years<br>• Female gender: 42% women<br>• Number of medications: 10 ±3.5<br>• 18% used Personalised Dosage System (PDS) at discharge<br>• 44% caregiver at interview	Clinical Pharmacist	-Counselling<br>-Support tool: personalised medication plan<br>-Follow-up: telephone	2
Shim et al. 2018	RCT	Malaysia/ Secondary care (Medical Outpatient Department+ Hospital Pharmacy)	I: 80<br>C: 80	• Age: 72 [7.0] years<br>• Female gender: 42.5% women<br>• Comorbidities: 4.0 [1.0]<br>• Marital status:<br>Married: 64.4%<br>Single/ divorced/ widow(er): 35.6%<br>• Education level:<br>No formal education: 26%<br>Primary education: 42.5%<br>Secondary education/ diploma/ tertiary education: 31.5%	• Age: 71.0 [6.0] years<br>• Female gender: 43.0% women<br>• Comorbidities: 5.0 [3.0]<br>• Marital status:<br>Married: 70.9%<br>Single/ divorced/ widow(er): 29.1%<br>• Education level:<br>No formal education: 35.4%<br>Primary education: 38.0 %<br>Secondary education/ diploma/ tertiary education: 26.6%	Pharmacist-Physician	-Medication review<br>-Reconciliation<br>-Counselling<br>-Follow-up: face-to-face	6
Syafhan et al. 2021	RCT	United Kingdom/ Primary care	I: 181<br>C: 175	• Age: 68.5 ± 13.5 years<br>• Female gender: 51.9% women<br>• Comorbidities: 7.3± 3.3<br>• Number of medications: 10.3 ± 3.7	• Age: 67.5 ± 12.6 years<br>• Female gender: 56.0 % women<br>• Comorbidities: 7.9 ± 3.6<br>• Number of medications: 10.3 ± 3.5	Clinical pharmacist	-Medication review<br>-Deprescribing<br>-Counselling<br>-Follow-up: face-to-face	6
Taylor et al. 2003	RCT	United States/ Primary care	I: 33<br>C: 36	• Age: 64.4 ± 13.7 years<br>• Female gender: 63.6% women<br>• Number of medications: 6.3 ± 2.2<br>• Education level: 12 years [4–16]<br>• Marital status:<br>Married: 75.8%<br>Widowed/Divorced: 24.2%	• Age: 66.7 ± 12.3 years<br>•Female gender: 72.2% women<br>•Number of medications: 5.7 ± 1.7<br>•Education level: 12 years [8–16]<br>•Marital status:<br>Married: 72.2%<br>Widowed/Divorced: 27.8%	Pharmacist	-Medication review<br>-Counselling / Education<br>-Compliance strategies<br>-Support tools: written materials<br>-Follow-up: face-to-face	12
Wu et al. 2006	RCT	China/ Secondary care (Specialist medical clinic)	I: 219<br>C: 223	• Age: 71.2 ± 9.4 years<br>• Female gender: 51% women<br>• Number of medications: 6 ± 1.3 chronic medications<br>• 95% administered drugs themselves<br>•14% lived alone	• Age: 70.5 ± 11.1 years<br>• Female gender: 52% women<br>• Number of medications: 5.9 ± 1.2<br>• 91% administered drugs themselves<br>• 5% lived alone	Pharmacist	-Education<br>-Counselling<br>-Follow-up: telephone<br>-Support tool: education materials at the screening visit	24
Yang et al. 2022	RCT	China/ Primary care	I: 67<br>C: 69	• Age: 70.76 ±7.49 years<br>• Female gender: 68.7% women<br>• Comorbidities:<br>Hypertension: 76.1%<br>Coronary heart disease: 59.7%<br>Stroke and cerebrovascular disease: 35.8%<br>Inflammatory connective tissue disorders: 38.8%<br>Lipid disorder: 49.3 %<br>Chronic painful condition: 26.9 %<br>• Number of medications: 4.36 ±2.11<br>• Education level:<br>Iliteracy: 10.4%<br>Elementary school: 28.3%<br>Junior high school: 29.9%<br>Senior high school: 25.4%<br>Technical school or college: 6.0%<br>• Marital status:<br>Married: 74.6%<br>Widowed/Divorced: 25.4%	• Age: 72.67 ±7.64 years<br>• Female gender: 52.2% women<br>• Comorbidities:<br>Hypertension: 65.2%<br>Stroke and cerebrovascular disease: 40.6%<br>Inflammatory connective tissue disorders: 34.8%<br>Lipid disorder: 23.2%<br>Coronary heart disease: 43.5%<br>Chronic painful condition: 40.6%<br>• Number of medications: 3.70 ±2.34<br>• Education level:<br>Iliteracy: 11.6%<br>Elementary school: 30.4%<br>Junior high school: 29.0%<br>Senior high school: 17.4%<br>Technical school or college: 11.6%<br>• Marital status:<br>Married: 74.6%<br>Widowed/Divorced: 25.4%	Community Nurse	-Education<br>-Motivational Interviewing<br>-Self-Management Skills training<br>-Follow-up: telephone	3

SUPPLEMENTARY TABLE 4a. RCTs								
Author/ Year	Study design	Country/ Setting	Sample (n)	Intervention group characteristics	Control group characteristics	Intervention provider	Intervention type	Follow-up period (months)
				Frequency (percentage), mean ± SD/ (range) or median [IQR], mean (95% CI)				

I: Intervention
C: Control
1. The ISAR screening tool was used to stratify patients into
two groups: those more likely to represent to an ED or be admitted (ISAR positive = score of 3 or more) and those less likely (ISAR negative = score of 2 or less)
2. The Charlson Comorbidity Score is a tool used to assess the overall burden of comorbidities (coexisting medical conditions) in a patient. Each condition is assigned a weight based on its association with mortality, and these weights are summed to create a total score. Higher scores indicate a greater burden of illness and a higher risk of mortality.
3. The Cumulative Illness Rating Scale (CIRS) is a tool used to assess the severity of a patient's comorbid conditions. It provides a detailed assessment by categorizing diseases into multiple body systems and assigning severity scores for each condition. Higher scores reflect more severe illness in multiple organ systems, helping to determine the overall health status of a patient.

SUPPLEMENTARY TABLE 4b. QUASIEXPERIMENTAL STUDIES								
**Author/ Year**	**Study design**	**Country/ Setting**	**Sample (n)**	**Intervention Group characteristics**	**Control Group characteristics**	**Intervention provider**	**Intervention type**	**Follow-up period (months)**
				**Frequency (percentage), mean ± SD/ (range) or median [IQR], mean (95% CI)**				
Al-Rashed et al. 2002	Quasiexperimental	United Kingdom Hospital discharge	I: 45<br>C: 44	• Age: 80.2 ±5.7 years<br>• Female gender: 37.2% women<br>• Number of medications:7.1± 1.8	• Age: 81.1 ±5.8 years<br>• 50.0% women<br>• Number of medications: 7.1 ±2.3	Clinical pharmacist	-Counselling<br>-Support tools: MIDS (Medication and Information Discharge Summary sheets)/ Medication reminder cards<br>-Follow-up: Home visits	3
Hugtenburg et al. 2009	Quasiexperimental	Netherlands/ Community pharmacy	I: 336<br>C: 379	• Age: 69.7 ± 15.0 years<br>• Female gender: 51.2% women<br>•Disease status:<br>- Patients from cardiologic, internal and pulmonary departments: 81.6%<br>•Number of medications: 7.8 ± 2.6	• Age: 72.7 ± 11.2 years<br>• Female gender: 53.3% women<br>• Disease status:<br>- Patients from cardiologic, internal and pulmonary departments: 83.7%<br>• Number of medications: 7.1 ± 2.3	Community pharmacist	-Medication review<br>-Counselling<br>-Follow-up: Home-visits<br>-Support tools: daily medication intake scheme	9
Karapinar-Çarkıt et al. 2019	Quasiexperimental ITS	Netherlands/ Hospital discharge	I: 365 (After)<br>C: 341 (Before)	• Age: 66.7 ± 16.0 years<br>• Female gender: 52.3 % women<br>• Number of medications: 6.9 ± 4.0<br>• Comorbidities: 3.9 ± 2.4<br>• Education level: no or low: 82.1%<br>• Marital status: married/partner: 42.3%<br>• Help with medication use: 30.8%	• Age: 64.3 ± 16.7 years<br>• Female gender: 48.4 % women<br>• Number of medications: 6.5 ± 3.5<br>• Comorbidities: 3.4 ± 2.1<br>• Education level: no or low: 78.5%<br>• Marital status: married/partner: 44.6%<br>• Help with medication use: 18.8%	Pharmaceutical consultants, supervised by clinical pharmacists	-Medication reconciliation<br>-Support tool: written medication summary	6
Leendertse et al. 2013	Quasiexperimental	Netherlands / Primary care	I: 364<br>C: 310	• Age: 75.8 (74.9–76.4) years<br>• Female gender: 56.0 % women<br>• Number of medications: 7.8 (7.7– 8.2)<br>• Comorbidities: 5.2 (4.7–5.6)	• Age: 75.7 (75.1–76.7) years<br>• Female gender: 60.0 % women<br>• Number of medications: 7.9 (7.5–8.2)<br>• Comorbidities: 3.4 (3.0–3.9)	Pharmacist	-Medication Review<br>-Pharmaceutical care plan<br>-Follow-up: face-to-face visits	8
Matzke et al. 2018	Quasiexperimental	United States / Primary care	I: 2,480<br>C: 2,480	• Age: 65.2 ± 13.0<br>• Female gender: 57.4% women<br>• Comorbidities: 3.3 ±1.3	• Age: 65.5 ± 14.2<br>• Female gender: 57.9% women<br>• Comorbidities: 3.2 ±1.2	Pharmacist-Physician	-Medication Review<br>-Empowerment and self- management<br>-Follow-up: face-to-face/ telephone	12
Moczygemba et al. 2011	Quasiexperimental	United States / Home-based	I: 60<br>C: 60	• Age: 71.2 ± 7.5 years<br>• Female gender: 51.7% women<br>• Number of medications: 13.0 ± 3.2<br>• Comorbidities: 6.5 ± 2.3	• Age: 73.9 ± 8.0 years<br>• Female gender: 28.3% women<br>• Number of medications: 13.2 ± 3.4 medications<br>• Comorbidities: 7.0 ± 2.1	Clinical pharmacist	-Medication Review<br>-Follow-up: telephone	6
Moreno et al. 2021	Quasiexperimental	United States / Primary care	I: 648<br>C: 1,944	• Age: 70 [60–79] years<br>• Female gender: 54.8% women<br>• Disease status (%):<br>Congestive Heart Failure: 21.9%<br>Hypertension: 87.0%<br>Chronic Kidney disease: 34.6%	• Age: 69 [60–79] years<br>• Female gender: 54.6% women<br>• Disease status (%):<br>Congestive Heart Failure: 18.3%<br>Hypertension: 87.0%<br>Chronic Kidney disease: 32.8%	Pharmacist-Physician	-Medication Reconciliation<br>-Motivational Interviewing<br>-Follow-up: face-to-face visits	12
Odeh et al. 2019	Quasiexperimental	Ireland / Hospital discharge	I: 211<br>C: 211	• Age: 68.8 years ± 12.4 years<br>• Female gender: 48.34% women<br>• Number of medications: 12.6 ± 3.4	• Age: 68.8 ± 12.4 years<br>• Female gender: 48.34% women<br>• Number of medications: 12.5 ± 3.8	Clinical pharmacist	-Medication review<br>-Education<br>-Follow-up: telephone	3

SUPPLEMENTARY TABLE 4b. QUASIEXPERIMENTAL STUDIES								
**Author/ Year**	**Study design**	**Country/ Setting**	**Sample (n)**	**Intervention Group characteristics**	**Control Group characteristics**	**Intervention provider**	**Intervention type**	**Follow-up period (months)**
				**Frequency (percentage), mean ± SD/ (range) or median [IQR], mean (95% CI)**				
Perman et al. 2021	Quasiexperimental	Argentina/ Home-based	I: 121<br>C: 121	• Age: 84.5 years<br>• Female gender: 62.8 % women<br>• Diabetes mellitus: 13.2 %<br>• Dyslipidaemia: 48.8 %<br>• Hypertension: 85.1 %<br>• Coronary heart disease: 17.4 %<br>• Cerebrovascular disease: 19.8 %<br>• Ischaemic peripheral artery disease: 7.4 %<br>• Heart failure: 26.5 %<br>• Chronic kidney disease: 18.2 %<br>• Dementia: 10.7 %<br>• Depression: 24.8 %<br>• Chronic obstructive pulmonary disease: 9.1 %<br>• Malignant neoplasm: 22.3 %<br>• Home-based primary care: 44.6 %	• Age: 86.2 years<br>• Female gender: 73.6 % women<br>• Diabetes mellitus: 15.7 %<br>• Dyslipidaemia: 49.6 %<br>• Hypertension: 84.3 %<br>• Coronary heart disease: 21.5 %<br>• Cerebrovascular disease: 25.6 %<br>• Ischaemic peripheral artery disease: 9.1 %<br>• Heart failure: 24.8 %<br>• Chronic kidney disease: 14.1 %<br>• Dementia: 12.4 %<br>• Depression: 28.9 %<br>• Chronic obstructive pulmonary disease: 9.9 %<br>• Malignant neoplasm: 23.1 %<br>• Home-based primary care: 55.4 %	Health and social care counsellor	-Comprehensive assessment: social, functional, and biological domains<br>-Medication reconciliation and adherence<br>-Coordination with health and social services<br>-Follow-up: home visits (depending on needs)	12
Reidt et al. 2016	Quasiexperimental	United States/ SNF discharge	I: 87<br>C: 189	• Age: 70.8 years<br>• Female gender: 57% women<br>• Charlson Comorbidity Index: 1.0	• Age: 69.7 years<br>• Female gender: 60% women<br>• Charlson Comorbidity Index: 1.3	Pharmacist-Nurse-Geriatrician	-Medication review<br>-Education / counselling<br>-Support tool: updated EHR / medication list<br>-Follow-up: home visit or telephone	1
Westberg et al. 2014	Quasiexperimental	United Kingdom / Primary Care	I: 135<br>C: 270	• Age: 75.9 ±7.0 years<br>• Female gender: 48.1% women<br>• Number of medications: 8 ±7.6	• Age: 76.2 ± 7.9 years<br>• Female gender: 48.1% women<br>• Number of medications: 14.3± 7.6	Pharmacist	-Medication review<br>-Education<br>-Follow-up: telephone/face- to-face	6

I: Intervention
C: Control
SNF: Skilled Nursing Facility
