# Supplementary material for: Interventions to enhance in-home taking medication among older adults with multimorbidity/polypharmacy: a systematic review and meta-analysis
Source: Front Public Health. 2026 Jan 28;13:1701622. doi: 10.3389/fpubh.2025.1701622 (PMC12891206; doi:10.3389/fpubh.2025.1701622)
Supplement: Supplementary file 1 [file Data_Sheet_1.zip › Supplementary Table 5.Interventions - Supplementary table 5-Interventions.pdf]

SUPPLEMENTARY TABLE 5. INTERVENTIONS

Author, Year	INTERVENTION												
	MEDICATION MANAGEMENT					EDUCATIONAL INTERVENTIONS				TECHNOLOGY AND DIGITAL TOOLS			HEALTH AND SOCIAL INTERVENTION
	MEDICATION REVIEW	MEDICATION RECONCILIATION	DEPRESCRIBING	PHARMACEUTICAL CARE PLAN	STRUCTURED DISCHARGE PLANNING	EDUCATION	COUNSELLING	MOTIVATIONAL INTERVIEWING	SKILLS-TRAINING	WEB-BASED APPLICATION	PERSONAL HEALTH RECORD	ELECTRONIC DECISION SYSTEM	SOCIAL, FUNCTIONAL AND BIOLOGICAL ASSESSMENT
Aguiar et al. 2018	X					X							
Ahmad et al. 2012/ Van der Heijden et al. 2019	X					X	X	X					
Bernsten et al. 2001	X		X			X							
Biswas et al. 2018							X						
Bolas et al. 2004	X	X			X		X						
Briggs et al. 2015	X												
Campins et al. 2017	X												
Chrischilles et al. 2014						X				X	X		
Del Cura-González et al. 2022	X												
Geurts et al. 2016	X									X			
Heaton et al. 2019	X	X				X	X	X					
Herrinton et al. 2023	X		X										
Holland et al. 2005	X					X							
Insel et al. 2012						X							
Jarab et al. 2012	X					X		X					
Jerant et al. 2009						X			X				
Köberlein-Neu et al. 2016	X												
Kouladjian O'Donnell et al. 2021	X		X									X	
Lee et al. 2006						X							
Lembeck et al. 2019					X								
Lenaghan et al. 2007	X					X							
Lenander et al. 2014	X						X						
Martínez-Mardones et al. 2023	X					X							
McCarthy et al. 2022	X		X										
Messerli et al. 2016	X					X	X						
Morales Suárez-Varela et al. 2009						X							
Muth et al. 2018	X	X										X	
Nazareth et al. 2001	X				X		X						
Olesen et al. 2014	X		X			X		X					
Poorcheraghi et al. 2023						X	X			X			
Sáez de la Fuente et al. 2011						X							

Author, Year	ADHERENCE SUPPORT TOOLS										FOLLOW-UP				
	MEMORY STRATEGIES	EDUCATIONAL LEAFLETS	MEDICATION PACKAGING	MEDICATION PLAN	MEDICINES HELPLINE (CARD)	REMINDERS	TIME-TABLE CHARTING	WRITTEN MEDICATION SUMMARY	WRITTEN MATERIALS	EDUCATIONAL BOOKLET	TELEPHONE	VIDEO	E-MAIL	FACE-TO-FACE	HOME VISIT
Aguiar et al. 2018		X									X				
Ahmad et al. 2012/ Van der Heijden et al. 2019				X										X	X
Bernsten et al. 2001						X									
Biswas et al. 2018							X				X			X	
Bolas et al. 2004				X	X										
Briggs et al. 2015															
Campins et al. 2017															
Chrischilles et al. 2014											X		X		
Del Cura-González et al. 2022				X										X	
Geurts et al. 2016						X									
Heaton et al. 2019		X	X	X							X			X	
Herrinton et al. 2023											X	X			
Holland et al. 2005			X												X
Insel et al. 2012	X		X												X
Jarab et al. 2012										X				X	
Jerant et al. 2009											X				X
Köberlein-Neu et al. 2016															X
Kouladjian O'Donnell et al. 2021															
Lee et al. 2006			X											X	
Lembeck et al. 2019															X
Lenaghan et al. 2007														X	
Lenander et al. 2014															
Martínez-Mardones et al. 2023														X	
McCarthy et al. 2022															
Messerli et al. 2016				X							X			X	
Morales Suárez-Varela et al. 2009			X								X				X
Muth et al. 2018											X			X	
Nazareth et al. 2001				X											X
Olesen et al. 2014		X									X				
Poorcheraghi et al. 2023						X					X			X	
Sáez de la Fuente et al. 2011															

SUPPLEMENTARY TABLE 5. INTERVENTIONS

Author, Year	INTERVENTION												
	MEDICATION MANAGEMENT					EDUCATIONAL INTERVENTIONS				TECHNOLOGY AND DIGITAL TOOLS			HEALTH AND SOCIAL INTERVENTION
	MEDICATION REVIEW	MEDICATION RECONCILIATION	DEPRESCRIBING	PHARMACEUTICAL CARE PLAN	STRUCTURED DISCHARGE PLANNING	EDUCATION	COUNSELLING	MOTIVATIONAL INTERVIEWING	SKILLS-TRAINING	WEB-BASED APPLICATION	PERSONAL HEALTH RECORD	ELECTRONIC DECISION SYSTEM	SOCIAL, FUNCTIONAL AND BIOLOGICAL ASSESSMENT
Sánchez Ulayar et al. 2011							X						
Shim et al. 2018	X	X					X						
Syafhan et al. 2021	X		X				X						
Taylor et al. 2003	X					X	X						
Wu et al. 2006						X	X						
Yang et al. 2022						X		X	X				
Al-Rashed et al. 2002							X						
Hugtenburg et al. 2009	X						X						
Karapinar-Çarkıt et al. 2019		X											
Leendertse et al. 2013	X			X									
Matzke et al. 2018	X								X				
Moczygemba et al. 2011	X												
Moreno et al.2021	X	X						X					
Odeh et al. 2019	X					X							
Perman et al. 2021		X											X
Reidt et al. 2016	X						X						
Westberg et al. 2014	X					X							

**Author, Year**	**ADHERENCE SUPPORT TOOLS**										**FOLLOW-UP**				
	MEMORY STRATEGIES	EDUCATIONAL LEAFLETS	MEDICATION PACKAGING	MEDICATION PLAN	MEDICINES HELPLINE (CARD)	REMINDERS	TIME-TABLE CHARTING	WRITTEN MEDICATION SUMMARY	WRITTEN MATERIALS	EDUCATIONAL BOOKLET	TELEPHONE	VIDEO	E-MAIL	FACE-TO-FACE	HOME VISIT
Sánchez Ulayar et al. 2011				**X**							**X**				
Shim et al. 2018														**X**	
Syafhan et al. 2021														**X**	
Taylor et al. 2003									**X**						
Wu et al. 2006		**X**									**X**				
Yang et al. 2022											**X**				
Al-Rashed et al. 2002				**X**		**X**									**X**
Hugtenburg et al. 2009							**X**								**X**
Karapinar-Çarkıt et al. 2019								**X**							
Leendertse et al. 2013														**X**	
Matzke et al. 2018											**X**			**X**	
Moczygemba et al. 2011											**X**				
Moreno et al.2021														**X**	
Odeh et al. 2019											**X**				
Perman et al. 2021															**X**
Reidt et al. 2016								**X**			**X**				**X**
Westberg et al. 2014											**X**			**X**	
