# Supplementary material for: Interventions to enhance in-home taking medication among older adults with multimorbidity/polypharmacy: a systematic review and meta-analysis
Source: Front Public Health. 2026 Jan 28;13:1701622. doi: 10.3389/fpubh.2025.1701622 (PMC12891206; doi:10.3389/fpubh.2025.1701622)
Supplement: Supplementary file 1 [file Data_Sheet_1.zip › Supplementary Table 6a-6b.Risk of Bias of Included Studies.pdf]

SUPPLEMENTARY TABLE 6a. Risk Of Bias RCTs

Author/Year	BIAS ARISING FROM THE RANDOMISATION PROCESS (R)		BIAS DUE TO DEVIATIONS FROM INTENDED INTERVENTIONS (D)		BIAS DUE TO MISSING OUTCOME DATA (Mi)		BIAS IN MEASUREMENT OF THE OUTCOME (Me)		BIAS IN SELECTION OF THE REPORTED RESULT (S)		OTHER BIAS	OVERALL RISK OF BIAS (O)
	Support for judgement	Risk of bias judgement	Support for judgement	Risk of bias judgement	Support for judgement	Risk of bias judgement	Support for judgement	Risk of bias judgement	Support for judgement	Risk of bias judgement		
Aguiar et al. 2018	Randomisation was performed by pharmacist researcher using a computer-generated randomized list from the research randomizer programme (https://www.randomizer.org) and followed the allocation sequence according to the referral of the physicians. Baseline characteristics were similar between the intervention and control groups.	L	Patients and the pharmacist were aware of the allocation of the control and intervention groups. The medical team was unaware of the patients who were randomised to the control group; but they knew on the intervention group, because any suggestions for adjustment of therapy were discussed with them. All eligible patients were informed by pharmacist. It is unclear whether the intervention was monitored, as no details are provided on how deviations from the protocol were identified or addressed.	?	Attrition rate < 10%, reasons for loss to follow-up were described. Only data of the patients who completed this study were analysed. Lost to follow-up was balanced between the groups and no difference was noted for the characteristics of patients and reasons for withdrawal.	?	The outcome assessors were blinded. Adherence was measured using 4-item Morisky–Green test, a validated tool; however it relies on self-report data.	?	No previously registered protocol was accessible, which prevents a comparison between the reported outcomes and those originally planned. While the authors present results relevant to the stated objectives, the lack of access to a registered protocol limits the ability to fully rule out selective reporting bias. However, the authors report that primary and secondary outcomes were analysed, and the lack of economic analysis due to missing data is acknowledged.	?	?	SOME CONCERNS
Ahmad et al. 2012/ Van der Heijden et al. 2019	Pharmacies were electronically randomised as a control or intervention pharmacy. Explicit description of random sequence generation and allocation concealment is not provided.	H	Both participants and personnel delivering the interventions were aware of the intervention assignment. It is unclear whether the intervention was monitored, as no details are provided on how deviations from the protocol were identified or addressed. Missing data were handled by multiple imputation. Additional analyses were done on complete cases.	?	Reasons for missing data were not detailed. 35 patients (10.3%) died during the follow-up. Only 63.5% of patients (216 out of 340) completed at least one cost calendar, and 36.2% of patients completed all six cost calendars during the first six months.	?	Outcomes were assessed blindly by two clinical pharmacologists. DRPs were assessed both subjectively (via self-report) and objectively (via medication analysis). Costs and healthcare utilization were self-reported by patients.	?	No evidence of selective reporting.	L	?	HIGH RISK OF BIAS
Bernsten et al. 2001	Half of the recruited sites were randomly assigned as control sites and half as intervention sites and, where possible, control and intervention sites were matched as closely as possible according to size (total number of patients served), situation (city centre vs village) and type (owned by a single proprietor vs part of a national chain). Lack of information about random sequence generation and allocation concealment.	H	There is no explicit mention of blinding patients/ personnel. Training of the pharmacists was not standardised and was not rigurouisly controlled. It is unclear whether the intervention was monitored, as no details are provided on how deviations from the protocol were identified or addressed	H	High attrition rate, reasons for follow-up were described. Despite patients who were lost to follow-up being older and reporting poorer quality of life compared with those who completed the study, the profiles of control and intervention patients who dropped out were otherwise similar.	H	No information of whether the outcome assessors were blinded. Knowledge of medicines, conmpliance, physician visits were self-reported by the patient.	?	No previously registered protocol was accessible, which prevents a comparison between the reported outcomes and those originally planned. While the authors present results relevant to the stated objectives, the lack of access to a registered protocol limits the ability to fully rule out selective reporting bias.	?	?	HIGH RISK OF BIAS
Biswas et al. 2018	Using online random number generator (www.random.org), a simple randomisation plan for this study was done for allocation of patients. Lack of information on how the randomisation sequence was concealed until interventions were assigned is a concern. The baseline characteristics were balanced except for the sex distribution.	?	The investigator did not attempt to treat or modify the ongoing management by their physicians. However, dropouts were noted, and attempts to contact them were made. Handling deviations and adherence were partially addressed. The analysis used the Last Observation Carried Forward (LOCF) method for handling missing data.	?	High attrition rate. No reasons for dropout were provided. No Intention To Treat Analysis was performed.	H	There is no explicit mention of blinding the outcome assessors. Lack of blinding could introduce measurement bias, especially in self-reported outcomes like medication adherence. The use of validated tools like the Charlson Comorbidity Index, Morisky Medication Adherence Scale, but the absence of blinding remains a concern.	?	No previously registered protocol was accessible, which prevents a comparison between the reported outcomes and those originally planned. While the authors present results relevant to the stated objectives, the lack of access to a registered protocol limits the ability to fully rule out selective reporting bias.	?	?	HIGH RISK OF BIAS
Bolas et al. 2004	Patients were randomised into intervention or control groups using computer-generated random numbers. It is not explicitly mentioned whether allocation was concealed from the researchers or patients before assignment.	L	No information of blinding of participants or personnel. The study does not specify if intended intervention was delivered as planned. The study does not describe how missing data were handled.	H	High attrition rate. Reasons for loss to follow-up were provided.	H	No information of whether the outcome assessors were blinded. The methods for assessing outcomes (patient recall and medication history accuracy) were subjective.	?	No previously registered protocol was accessible, which prevents a comparison between the reported outcomes and those originally planned. While the authors present results relevant to the stated objectives, the lack of access to a registered protocol limits the ability to fully rule out selective reporting bias.	?	?	HIGH RISK OF BIAS
Briggs et al. 2015	The study used block randomisation by strata with block sizes of 4 or 6, performed by an independent statistical unit using a random number generator with a seed. The randomisation list was held by the study coordinator . Stratification was done based on the ISAR score. Baseline characteristics were evenly distributed.	?	Lack of allocation concealment and potential awareness of intervention by clinicians could lead to deviations. All the patients allocated to intervention group received the intervention and all patients in the usual care group received usual care.	?	All included patients completed the study.	L	Outcomes were assessed by contacting patients, their GPs, and community pharmacies to obtain an updated medication history and to determine whether the patient had re-presented to the ED. Outcome assessors were blinded to the group allocation.	L	No previously registered protocol was accessible, which prevents a comparison between the reported outcomes and those originally planned. While the authors present results relevant to the stated objectives, the lack of access to a registered protocol limits the ability to fully rule out selective reporting bias.	?	?	SOME CONCERNS
Campins et al. 2017	Randomisation was based on a list of random numbers generated by a statistical program. Allocation was concealed using sealed, opaque envelopes prepared by the research unit, not at primary care centers, ensuring that allocation was not known before it was assigned. Baseline characteristics were similar between the intervention and control groups.	L	Open-label study. Lack of periodic intervention reminders could lead to deviations. Intention-to-treat analysis was performed.	?	Low attrition rate. All patients were included in analysis as randomised.	L	Outcome assessors were not blinded. It may influence self-reported outcomes such quality of life (measured using EuroQoL-5D) and adherence (measured using the Morisky-Green test). However, the impact of lack of blinding on objective outcomes (hospitalisation rates, primary care and emergency department consultation rate for acute conditions) is low.	?	There is no evidence of selective reporting.	L	CONTAMINATION BIAS	SOME CONCERNS
Chrischilles et al. 2014	This was a single-center open-label parallel-group study with unequal randomisation (3:1 ratio). No information about the random sequence generation nor allocation concealment. The study groups were well balanced at baseline.	?	Ope-label study. Participants in the intervention group were provided with access to the Iowa PHR system and supporting materials (e.g., quick-start guide, login credentials, and reminder letters). However, there was variable engagement with the system: only 61.2% attempted to log in, and 55.2% performed any activity, with 42.5% actually entering health information. As-treated analyses were performed comparing high users to low/non-users.	?	Acceptable attrition rate. Reasons for dropout were described. No intention to treat analysis, characteristics of losses to follow-up were not described.	?	Open-label study. Outcome measures were obtained from both self-reported questionnaire data and objective system log data (login attempts, activity logs, medication entries, and warning messages). These outcomes are largely objective (number of medications entered, system engagement) and were assessed using standardised methods.	L	No evidence of selective reporting.	L	?	SOME CONCERNS
Del Cura-González et al. 2022	Simple randomisation was performed centrally using the software Epidat 4. No relevant differences were found between study arms at baseline in demographic or clinical characteristics or in the study's primary or secondary outcomes.	L	Physicians were aware of their treatment allocation. Strategies to improve the protocol adherence of FPs were implemented, including individual follow-up on the protocol's milestones and queries, as well as incentives such as messages of appreciation via e-mail, acknowledgement of their contribution through an invitation to co-author scientific reports, and continuous professional development certified training sessions. Missing data were analysed using the last observation carried forward (LOCF), as well as through multiple imputation.	?	Losses to follow-up were small, less than 8% at the 12-month follow-up. The losses in the intention-to-treat analysis were imputed by the multiple imputation. A final sensitivity analysis (complete cases, ITT LOCF, and multiple imputed) was added. No difference was found in the results, probably due to limited losses.	L	All analyses were performed by the trial statisticians, who were blinded to the group assignment. Medication adherence was measured using Morisky-Green test and quality of life was measured using EuroQol 5D-5L (validated tools) Use of health services was measured as unplanned and/or number of hospitalisations, number of visits to emergency services, and number of FP and primary care nurse visits.	L	The study did not report results for the therapeutic adherence test (Haynes-Sackett test) , despite being pre-specified in the protocol, without providing a clear justification.	H	CONTAMINATION BIAS	HIGH RISK OF BIAS
Geurts et al. 2016	Randomisation occurred based on unique patient identification numbers in the pharmacy computer system (odd number: intervention group; even number: control group). Concealment not explicitly stated The baseline characteristics between groups were comparable.	?	Pharmacists and GPs were aware of the group assignments since they had to perform the interventions. Participants in the intervention group were invited to consult their pharmacist for a CMR, which makes them aware of their group assignment. A learning module of the W-PCP application was provided by the researchers to all participating pharmacists and GPs. During the study period technical assistance was available. All participating pharmacists received a 1-day training course on communication skills with GPs and patients. Additional written information about performing a CMR was provided. During the study period, researchers visited study sites regularly in order to monitor the time schedule of the study and provide assistance. The study allowed each site to develop its own organizational approach to implementing the intervention, leading to variability in execution. Not all intervention patients received the intended intervention due to time limitations. An intention-to-treat analysis was performed.	?	Although 70 patients from the intervention group did not receive any part of the intervention, they were analyzed separately. There were no significant baseline differences between those who received the intervention and those who did not. .	?	There is no explicit mention of blinding of the outcome assessors. Two researchers independently coded all individual care plans. All codes were compared, and inconsistencies were discussed until consensus was reached. DRPs were resolved based on the researchers' interpretation, which introduces subjectivity. For control group patients, information on care interventions was collected retrospectively.	?	No previously registered protocol was accessible, which prevents a comparison between the reported outcomes and those originally planned. While the authors presented results relevant to their stated objectives, the lack of access to a registered protocol limits the ability to rule out selective reporting bias completely.	?	?	SOME CONCERNS

SUPPLEMENTARY TABLE 6a. Risk Of Bias RCTs

Author/Year	BIAS ARISING FROM THE RANDOMISATION PROCESS (R)		BIAS DUE TO DEVIATIONS FROM INTENDED INTERVENTIONS (D)		BIAS DUE TO MISSING OUTCOME DATA (Mi)		BIAS IN MEASUREMENT OF THE OUTCOME (Me)		BIAS IN SELECTION OF THE REPORTED RESULT (S)		OTHER BIAS	OVERALL RISK OF BIAS (O)
	Support for judgement	Risk of bias judgement	Support for judgement	Risk of bias judgement	Support for judgement	Risk of bias judgement	Support for judgement	Risk of bias judgement	Support for judgement	Risk of bias judgement		
Heaton et al. 2019	Patients were randomised using a random number generator. There is no specific mention of whether allocation concealment was implemented. All variables were balanced between the groups except for age, sex, and insurance type.	?	The study does not mention blinding of patients or healthcare providers. Only 62 patients out of 213 received the intervention as intended. It includes both an intent-to-treat (ITT) analysis and a per-protocol (PP) analysis.	H	The high dropout rate affected the results of the ITT analysis.	H	No mention of blinding of outcome assessors. The primary outcome (readmission rate) is objective and less likely to be influenced by lack of blinding. Primary medication non-adherence (PMN) used the Kroger dispensing system to capture unfilled prescriptions that were sent electronically to the pharmachy and secondary non-adherence was measured by proportion of days covered (PDC), according to the Pharmacy Quality Alliance definitions.	L	No previously registered protocol was accessible, which prevents a comparison between the reported outcomes and those originally planned. While the authors present results relevant to the stated objectives, the lack of access to a registered protocol limits the ability to fully rule out selective reporting bias.	?	CONTAMINATION BIAS	HIGH RISK OF BIAS
Herrinton et al. 2023	Randomisation was conducted using simple random sampling by a research data analyst after physician authorization. Baseline characteristics were balanced.	L	This was a pragmatic trial, and blinding of participants and personnel was not feasible. The intervention included several components delivered by pharmacists, and the process was well-documented. However, pharmacists' adherence to the detailed intervention protocols was not explicitly reported. Intention To Treat analysis was performed.	?	Acceptable attrtition rate. Reasons for loss to follow-up were provided. Patients were analysed as randomised.	L	Blinding of outcome assessors was not reported. Outcomes were obtained from recorded clinical data, reducing the risk of measurement bias.	L	There is no evidence of selective reporting.	L	CONTAMINATION BIAS	LOW RISK OF BIAS
Holland et al. 2005	Patients were randomised using a computer-generated sequence in blocks of varying length. Allocation was managed through third-part telephone randomisation. Baseline characteristics between the intervention and control groups were similar.	L	Participants and personnel were not blinded due to the nature of the intervention. 67 patients (16%) in the intervention group did not receive the planned intervention. In addition, there was variability in the recommendation of adherence aids and in pharmacists' perceptions of the usefulness of the visits. Intention To Treat analysis was performed.	?	Acceptable attrtition rate. Reasons for loss to follow-up were provided. Patients were analysed as randomised.	L	The impact of lack of blinding of outcome assessors on objective outcomes (ED visits, deaths) is low. It may influence self-reported outcomes such quality of life (measured using EuroQoL-5D).	?	Medication costs (a secondary outcome) were not reported and primary care data (not pre-specified) were reported.	H	L	HIGH RISK OF BIAS
Insel et al. 2012	Block randomisation was performed. Lack of details on allocation concealment and whether the randomisation sequence was generated appropriately.	?	Patients or participants blinding was not mentioned. Nurses' adherence to the protocol was monitored using voice recordings during visits. Any deviations from the intervention protocol were reviewed and corrected with the project coordinator. The study does not describe how missing data were handled.	?	Causes for dropout were described. Acceptable attrition rate. No Intention To Treat Analysis was performed.	H	Blinding of outcome assessors was not reported. Medication adherence was measured using the MEMS® system , which provides an objective measure of when the medication container is opened.	L	No previously registered protocol was accessible, which prevents a comparison between the reported outcomes and those originally planned. While the authors present results relevant to the stated objectives, the lack of access to a registered protocol limits the ability to fully rule out selective reporting bias.	L	L	HIGH RISK OF BIAS
Jarab et al. 2012	Study participants were randomly assigned to intervention and control groups via a minimisation technique using MINIM software. There is no specific information about allocation concealment.	?	The study followed an open-label design; both participants and pharmacists were aware of group allocation. Adherence monitoring relied on self-report and no intention-to-treat analysis was performed.	H	The attrition rate was below 5 % and balanced between groups. The reasons for withdrawal were not reported. Analyses were conducted on participants who completed follow-up.	?	Open-label trial; most endpoints (quality of life, medication adherence) were self-reported.	H	No prospective protocol or trial registration was identified. Reported outcomes were consistent with study objectives, but selective reporting cannot be excluded.	?	CONTAMINATION BIAS	HIGH RISK OF BIAS
Jerant et al. 2009	Block randomisation, implemented by nurse at home visit, in blocks of 12 participants with sealed opaque envelopes containing group assignments.	?	Participants and intervention providers were not blinded. The intervention was delivered according to the protocol and was closely monitored . Other than sessions missed by early dropouts, 100% of HIOH intervention sessions were completed. The study does not describe how missing data were handled.	?	Causes for dropout were not described. Participant dropout was greater in the intervention groups. Low attrition rate. All randomised patients were analysed.	?	Standardised questionnaires and scales to assess outcomes (PT/PP, SF-36, EQ-5D). It is not clear whether those conducting the assessments were blinded to participants' group assignments.	?	Healthcare utilisation was reported as an outcome but was not specified in the pre-registered protocol.	H	L	HIGH RISK OF BIAS
Köberlein-Neu et al. 2016	The independent biometrician randomised the clusters. The study does, however, include a random regional sample, which includes medical practices that were willing to participate. This may lead to selection bias. In addition, patients were selected by the physicians themselves.	H	Patients blinding is not mentioned. All Advanced Care practitioners assigned to the intervention group received training on how to use and implement the CCDSS (Clinical Decision Support System) and were then tested for their competency. Adherence to the intervention was not clearly monitored or reported. Intention To Treat analysis included only patients whose MAI score was determined at baseline.	?	High attrition rate. Reasons for dropout were detailed. Dropouts were imbalanced across cohorts. Sample size was not achieved.	H	The pharmacists had been blinded when calculating scores as to which cohort a patient was allocated to, but they were involved in some cases in conducting the medication review. Outcomes were measured consistently using validated tools (subjective methods).	?	Some of the secondary outcomes specified in the pre-registered protocol were not reported.	H	?	HIGH RISK OF BIAS
Kouladjian O'Donnell et al. 2021	Randomisation was done with a 1:1 allocation using an online random number generator.	L	Pharmacists and patients were not blinded to the intervention. Pharmacists in the intervention arm received additional training and competency assessment. The methods for monitoring adherence to intervention were not reported. The study does not describe how missing data were handled.	?	Dropout rates were 28.4% for the intervention group and 15.0% for the comparison group. Causes for dropout were described. The study did not achieve the prespecified sample size. Only the patients who completed the study were included in anlysis.	H	Outcome measurement was done by pharmacists who were not blinded, this may influence self-reported outcomes such as adherence, impact on functioning, instititutionalisation or falls, despite validated questionnaires and scales were used.	?	Some of the secondary outcomes specified in the pre-registered protocol were not reported.	H	CONTAMINATION BIAS	HIGH RISK OF BIAS
Lee et al. 2006	Patients were randomised using a computer-generated random number sequence with allocation concealed from both participants and those enrolling them. Randomisation was done in blocks based on baseline adherence levels. Control and intervention groups were similar.	L	The trial clearly defined the intended interventions and followed up with participants to ensure adherence. Not possible to blind either the participants or personnel. Intention-to-treat principle and imputation of missing data using the last observation carried forward method was performed.	L	Acceptable attrition rate. Reasons for loss to follow-up were described. Among patients who did not complete the study phases, dropouts were more likely to be men.	?	The clinical pharmacists assessing the outcomes were not blinded. Outcomes such as medication adherence, were measured using objective methods (pill counts).	L	There is no evidence of selective reporting	L	L	LOW RISK OF BIAS
Lembeck et al. 2019	Computer-generated randomisation to allocate patients to the intervention or control group was used. Department staff were blinded to randomisation until the day before discharge. Control and intervention groups were similar.	L	Intervention was not blinded to the project nurse, municipal nurse and patient 88% of the intervention group received the planned intervention, but some patients did not due to unstable conditions at discharge. The study used intention-to-treat analysis and performed per-protocol analyses as a secondary measure.	?	The study reports complete follow-up with no loss to follow-up, as data were collected from nationwide registers.	L	The statistician who performed all analyses was blinded to the placement of patients in control or intervention group. Outcomes were measured using national registers which may be accurate.	L	The protocol was retrospectively registered.	?	CONTAMINATION BIAS	SOME CONCERNS
Lenaghan et al. 2007	Randomisation was carried out by a third party and was stratified by whether the patient lived alone. Groups were similar in baseline characteristics.	L	Blinding of participants and personnel was not mentioned. 94% of participants received the first visit, while four did not due to early withdrawal, refusal, or hospital admission; additionally, five more patients did not complete the second visit for similar reasons, potentially leading to deviations from the intended intervention. Results were analysed according to the Intention To Treat principle.	?	Primary outcome data were available for 99% of participants. Reasons for withdrawal were provided and were similar between groups. Quality-of-life data had high follow-up rates.	L	Hospital admissions and mortality data are objective and unlikely to be influenced by knowledge of intervention. Quality of life (EQ-5d) could be influenced by lack of blinding.	?	No previously registered protocol was accessible, which prevents a comparison between the reported outcomes and those originally planned. While the authors present results relevant to the stated objectives, the lack of access to a registered protocol limits the ability to fully rule out selective reporting bias.	?	L	SOME CONCERNS

SUPPLEMENTARY TABLE 6a. Risk Of Bias RCTs

Author/Year	BIAS ARISING FROM THE RANDOMISATION PROCESS (R)		BIAS DUE TO DEVIATIONS FROM INTENDED INTERVENTIONS (D)		BIAS DUE TO MISSING OUTCOME DATA (Mi)		BIAS IN MEASUREMENT OF THE OUTCOME (Me)		BIAS IN SELECTION OF THE REPORTED RESULT (S)		OTHER BIAS	OVERALL RISK OF BIAS (O)
	Support for judgement	Risk of bias judgement	Support for judgement	Risk of bias judgement	Support for judgement	Risk of bias judgement	Support for judgement	Risk of bias judgement	Support for judgement	Risk of bias judgement		
Lenander et al. 2014	No details on the method used for random sequence generation. The study does not specify whether allocation was concealed.<br><br>The two groups were similar at baseline except from phsychiatric disease. However, patients in the intervention group used a greater number of drugs.	H	Blinding of participants and personnel was not feasible due to the nature of the intervention.<br>The medication review was performed by a certified geriatrics pharmacist. The method had been tested in a pilot study. It involved a standardised semi-structured protocol that was open for patients' questions and remarks.<br>The methods for monitoring adherence to intervention were not reported.<br>The study does not describe how missing data were handled.	?	High attrition rate.<br>Reasons for dropout were not described.<br>Only the patients who completed the study were included in analysis.	H	An independent certified geriatrics pharmacist (BE), blinded to patient group allocation, analysed the DRPs. Utilisation of medical care was measured using records from Stockholm County Council, providing objective data.	L	No previously registered protocol was accessible, which prevents a comparison between the reported outcomes and those originally planned. While the authors present results relevant to the stated objectives, the lack of access to a registered protocol limits the ability to fully rule out selective reporting bias.	?	?	HIGH RISK OF BIAS
Martínez-Mardones et al. 2023	Simple randomisation was performed using MS Excel, with centres as the unit of randomisation.<br>Randomisation was conducted with at least one representative from each municipality acting as a witness.<br>Clusters were matched to reduce imbalance.	L	Patients were unaware of their group assignment, but pharmacists and clinical teams were aware.<br>Pharmacists in the intervention group were trained in the Polaris MRF method and supported by practice change facilitators.<br>Partial Intention To Treat and Multiple imputation was used for missing data.	?	Five centres dropped out within the first three months, and seven additional centres were lost due to pharmacists not conducting patient visits.<br>Patient dropout was balanced between groups (20% in the MRF group, 18% in the usual care group).	H	Pharmacists in the MRF group performed patient recruitment, interventions and data collection.	?	No evidence of selective reporting.	L	L	HIGH RISK OF BIAS
McCarthy et al. 2022	Minimisation with MinimPy software for randomisation was performed.<br>The randomisation was conducted by a trial statistician with no knowledge of the practices.<br>There were some differences in baseline characteristics (number of medicines). However, these were adjusted for in the analysis.	L	It was not possible to blind GPs or patients due to the nature of the intervention.<br>21.63% of the patients did not receive the intended intervention.<br>There was some evidence of deviations in the control group, with three control practices showing significant reductions in medicines.	H	8.66% of patients were lost to follow-up, and there was a difference in characteristics between those lost and those who remained.<br>Data analysis based on the Intention To Treat principle.<br>Sensitivity analyses were conducted.	?	Outcomes were measured by an independent blinded pharmacist for the primary outcomes.<br>However, self reported outcomes (collected through postal questionnaires) can lead to bias.	?	The protocol registered secondary outcomes, but some were modified or added during the study.	?	L	HIGH RISK OF BIAS
Messerli et al. 2016	The patients were assigned by 2 x 4 block randomisation into intervention or control group. Initially, each study pharmacist received two blocks containing eight dossiers (four intervention and four control) each packed in sealed and unlabelled envelopes.<br>There were no differences in baseline characteristics between groups.	?	Patients were aware of intervention.<br>A telephone interviewer's coaching and monitoring of compliance with the study protocol was continuously provided by an independent academic psychologist as external expert.<br>Missing data were handled by Intention To Treat analysis.	L	Only 18 patients (4.0 %) withdrew from the study.<br>Causes for dropout were described.<br>Attrition was balanced between groups.<br>Recruitment was stopped before the intended number of patients was recruited.	?	Telephone interviews were performed by trained independent clinical psychologists, blinded to the intervention. Patients' written self-reports were blinded to the pharmacists<br>Adherence was measured using several validated instruments (objective and subjective methods).	L	Healthcare resource utilisation was not specified in the pre-registered protocol, and its inclusion as an outcome could indicate selective reporting	H	CONTAMINATION BIAS	HIGH RISK OF BIAS
Morales Suárez-Varela et al. 2009	The study mentions random allocation in blocks but does not detail how allocation was concealed.<br>Baseline differences between groups in number of medications per day were reported.	?	Participants and caregivers were likely aware of the intervention due to the nature of the study (using a pill organizer).<br>The methods for ensuring adherence to the intervention were not explicitly reported.<br>Intention To Treat Analysis was performed.	?	The study does not indicate significant missing outcome data.<br>Drop-out causes were not described.<br>All patients were included in analysis.<br>Sample size was not achieved.	?	Outcome assessors were aware of the intervention. This may impact self-reported adherence, measure through Morisky-Green questionnaire, a validated instrument for measuring therapeutic adherence.	?	No previously registered protocol was accessible, which prevents a comparison between the reported outcomes and those originally planned. While the authors present results relevant to the stated objectives, the lack of access to a registered protocol limits the ability to fully rule out selective reporting bias.	?	?	SOME CONCERNS
Muth et al. 2018	Each practice was assigned in an allocation ratio of 1:1 using a block randomisation of variable block length. An external researcher generated the allocation sequence using the random number generator of Microsoft EXCEL.	L	The adherence to the protocol was monitored.<br>Due to the nature of the intervention, it was not possible to blind General Practitioners, Health care assistants, patients and the study team.<br>Treatment allocation was blinded to the clinical pharmacologist conducting medication reviews for the primary outcome.<br>The primary analysis was performed in accordance with the intention-to-treat principle, and additional sensitivity analyses were conducted on a per-protocol analysis set.	L	Low attrition rate.<br>Reasons for dropout were described.	L	Statistician was blinded to allocation. However, patients were not blinded so, self-reported adherence, patients' beliefs about medicines and functioning may be impacted (although they were measured using validated scales).	?	There is no evidence of selective reporting.	L	L	LOW RISK OF BIAS
Nazareth et al. 2001	Computer-generated random numbers for randomisation were used.<br>Randomisation was performed by the health authority's central community pharmacy office.	L	The study does not mention whether participants were blinded. Given the nature of the intervention (home visits by pharmacists), blinding participants was not possible.<br>Hospital and community pharmacists delivering the intervention were aware of the intervention.<br>A self-reported method was used to monitor the delivery of the intervention.<br>The study does not describe how missing data were handled.	?	High attrition rate.<br>Losses to follow up were balanced across groups and reasons for dropout were given.<br>No Intention To Treat Analysis was performed.	H	The outcomes were measured using standard procedures, such as hospital administration systems and validated questionnaires.<br>The research assistant collecting hospital outcome data was blinded to the allocation, which reduces the risk of differential measurement bias.<br>However, self-reported adherence may be influenced.	?	No previously registered protocol was accessible, which prevents a comparison between the reported outcomes and those originally planned. While the authors present results relevant to the stated objectives, the lack of access to a registered protocol limits the ability to fully rule out selective reporting bias.	?	?	HIGH RISK OF BIAS
Olesen et al. 2014	Envelopes were prepared with each containing a study inclusion code. At the first home visit by a project nurse, patients were asked to select one envelope.<br><br>No specific information is given about allocation concealment.<br><br>There were no significant differences between groups in baseline characteristics.	H	Blinding was not possible for participants or pharmacists in the intervention group due to the nature of the intervention.<br><br>Nine different pharmacists were involved in the MICMI study and adhered to the Danish manual for pharmaceutical care: 'Medication Review—Managing Medicine Manual' . The pharmacists must have some practical experience or courses in Medication Review' but no further training or standardisation was arranged.<br>No Intention To Treat analysis performed.	H	Overall dropout rates were similar to those of patients included in the final analysis with regard to sex, but different in relation to age (dropouts were older). More patients in the pharmaceutical care group dropped out due to patients' 'lack of interest'. The number of other reasons for dropping out was similar in the two groups.<br>The sample size for the primary outcome was not achieved due to a higher number of drop-outs than expected and it may also have been underpowered for the other outcomes.<br>There is no explicit mention of how missing data was handled.	H	The assessors were not blinded to the participants' allocation.<br>Adherence was measured by pill count with a 'counter pen.'<br>DRPs in relation to the patient's entire medicinal regimen were assessed by pharmacists at home-visits and classified into eight categories.<br>Data on unplanned hospital admissions to medical departments were obtained from the Danish e-Health Portal.<br>Mortality data were obtained from the hospital electronic record. It automatically records information on all deceased patients.	?	No previously registered protocol was accessible, which prevents a comparison between the reported outcomes and those originally planned. While the authors present results relevant to the stated objectives, the lack of access to a registered protocol limits the ability to fully rule out selective reporting bias.	?	?	HIGH RISK OF BIAS
Poorcheraghi et al. 2023	The study used block randomisation with a block size of four and an online randomisation tool.<br><br>The use of opaque envelopes for allocation concealment is appropriate.<br><br>The case and control groups were homogeneous in terms of demographic variables and drug adherence level before the intervention.	?	The description does not specify whether participants and researchers were blinded to group allocation.<br>The study took measures to ensure that the intervention was delivered as intended (training on app use, monitoring adherence, and app issues). The study does not describe how missing data were handled.	?	Reasons for dropout were reported.<br>Attrition rate was low and balanced between groups.<br>Data were analysed per protocol.	?	The Morisky Medication Adherence Scale and pill count method were used for adherence. These are valid methods, but self-report scales may introduce measurement bias.<br><br>A new researcher-made questionnaire was used to measure adverse events (it underwent validity testing, however it was self-reported). To neutralize this effect, the investigator compared the answer with their medical records and physician confirmation.	?	The lack of clarity regarding what is meant by "drug consequences" in the protocol and the explicit description of adverse events in the study raises concerns about selective reporting. It is unclear whether adverse events were initially planned as part of "drug consequences" but were reported separately or added later. This discrepancy prevents a full assessment of whether the outcomes reported in the study align with those originally planned in the protocol.	?	CONTAMINATION BIAS	SOME CONCERNS
Sáez de la Fuente et al. 2011	Patients were randomly assigned to intervention and control groups using a block method 1:1.<br>There is no information about allocation concealment.<br>There were no significant differences between groups in baseline characteristics.	?	Participants and personnel were not blinded.<br>The methods for ensuring adherence to the intervention were not explicitly reported.<br>An Intention To Treat Analysis was performed.	?	Reasons for dropout were reported.<br>High attrition rate.	H	The telephone interviewer was blinded to the treatment allocation, which minimizes detection bias.<br>Adherence was measured using the Morisky-Green test, which relies on self-reported data and this may lead to reporting bias.	?	No registered or mentioned protocol was identified in the manuscript, which prevents comparing the reported outcomes with those originally planned. The authors present results relevant to the stated objectives, but the absence of a registered protocol limits the ability to fully rule out selective reporting bias.	?	CONTAMINATION BIAS	HIGH RISK OF BIAS
Sánchez Ulayar et al. 2011	The study used closed envelopes containing cards with random numbers for group assignment.<br>If the envelopes were kept sealed until assignment the risk of bias would be low low.<br>There were no significant differences between groups in baseline characteristics.	?	The study does not specify whether participants or personnel were blinded to the group allocation.<br>No Intention To Treat analysis.<br>The methods for ensuring adherence to the intervention were not explicitly reported.	?	The study reports 18% losses and provides reasons. Characteristics of those lost to follow-up were not reported.	H	No information is given about blinding of the outcome assessors.<br>Objective outcomes such as hospitalisation rate may not be impacted; however medication discrepancies, assessed by a self-report questionnaire may be influenced by lack of blinding.	?	No registered or mentioned protocol was identified in the manuscript, which prevents comparing the reported outcomes with those originally planned. The authors present results relevant to the stated objectives, but the absence of a registered protocol limits the ability to fully rule out selective reporting bias.	?	CONTAMINATION BIAS	HIGH RISK OF BIAS

SUPPLEMENTARY TABLE 6a. Risk Of Bias RCTs												
**Author/Year**	**BIAS ARISING FROM THE RANDOMISATION PROCESS (R)**		**BIAS DUE TO DEVIATIONS FROM INTENDED INTERVENTIONS (D)**		**BIAS DUE TO MISSING OUTCOME DATA (Mi)**		**BIAS IN MEASUREMENT OF THE OUTCOME (Me)**		**BIAS IN SELECTION OF THE REPORTED RESULT (S)**		**OTHER BIAS**	**OVERALL RISK OF BIAS (O)**
	**Support for judgement**	**Risk of bias judgement**	**Support for judgement**	**Risk of bias judgement**	**Support for judgement**	**Risk of bias judgement**	**Support for judgement**	**Risk of bias judgement**	**Support for judgement**	**Risk of bias judgement**		
Shim et al. 2018	Participants were assigned to control or intervention groups using a computerised random number generator (Research Randomizer). There is no specific information about allocation concealment.<br><br>The study reports no significant differences between the intervention and control groups in terms of baseline characteristics.	?	Participants and personnel delivering the intervention were not blinded.<br>The protocol was followed for both groups.<br>Mechanisms to ensure follow-up such as phone call reminders, were applied.<br>A generali¡sed estimating equation (GEE) analysis was performed to confirm the effects of the collaborative interventions on participants' medication adherence and medication appropriateness based on MAI scores.	L	Low attrition rate, higher in the intervention group.<br>The reasons for dropout were not detailed.<br>No Intention To Treat Analysis.	?	The research assistant who assessed the outcomes was blinded to the group allocation.<br>Standardised and validated instruments (e.g., MALMAS for medication adherence) were used.	?	There is no evidence of selective reporting	L	CONTAMINATION BIAS	SOME CONCERNS
Syafhan et al. 2021	Random sequence was generated using random.org<br>The method used to conceal the allocation is not described.<br>There were no significant differences between groups at baseline; however, the number of active medical problems was slightly lower in the intervention group.	?	Pharmacists followed a structured protocol, including a pharmacist intervention guide to ensure uniformity. Forms were completed at different stages, helping to track adherence to the intervention.<br>Regular bi-weekly teleconferences and site visits ensured fidelity monitoring, reducing variation in how pharmacists delivered the intervention.Intention-to-treat analysis included all patients who were randomised and recruited (only for some outcomes).	?	Higher than expected loss to follow-up reduced statistical power.<br>The intervention schedule was perceived as burdensome, possibly leading to non-random dropout (systematic differences between those who remained and those who withdrew).<br>Deceased<br>patients were excluded from the healthcare resource utilisation aspect of the research, and therefore the latter<br>was not a pure ITT analysis.	H	Data collected from electronic records and standardised tools like MARS, BMQ, EQ-5D-5L. These are validated tools, but self-report scales may introduce bias.	?	There is no evidence of selective reporting	L	L	HIGH RISK OF BIAS
Taylor et al.<br>2003	Participants were randomly allocated to intervention or control groups, but the process used for random sequence generation and allocation concealment was not described.	H	Blinding was not possible due to the nature of the intervention. The study did not document the percentage of pharmacist recommendations that were implemented to assess whether differences existed between physicians and pharmacists in recommendation acceptance. Therefore, adherence to the intended intervention could not be verified. No intention-to-treat analysis was performed.	H	Twelve out of eighty-one participants (approximately 15%) did not complete the study. The reasons for loss to follow-up were not reported, and it was not specified whether attrition was balanced between the intervention and control groups. Moreover, no intention-to-treat analysis was conducted, and only participants who completed the 12-month follow-up were analysed.	H	Outcome assessors were not blinded. Outcomes included both self-reported measures (adherence and medication knowledge) and objective indicators extracted from medical records (hospitalisations and emergency department visits).	?	No prospective protocol or trial registration was identified. Reported outcomes were consistent with study objectives, but selective reporting cannot be excluded.	?	CONTAMINATION BIAS	HIGH RISK OF BIAS
Wu et al. 2006	The pharmacist was blinded to the randomisation sequence, which were computer generated by our statistician and sealed in envelopes labelled with consecutive numbers. The envelopes were opened by the clinic nurse.	L	Blinding was not possible because the intervention was complex and caregivers were involved.<br>For the primary analysis of the randomised group, an intention to treat analysis was performed. Deviations from the protocol that could have impacted the outcomes were not specified.	?	There is a high proportion of missing outcome data, as 60 patients (defaulters) were lost to follow-up. Baseline characteristics of defaulters were similar to those of randomised patients. However, defaulters had significantly worse outcomes, including higher mortality, lower compliance, and increased healthcare utilisation compared to those who completed the study.	H	Compliance was determined using a structured questionnaire (delivered by the pharmacist).<br>Information was checked against the dispensing information on Health Authority's clinical management system.<br>Hard endpoints such as all cause mortality and rates of admission to hospital were measured by review of death certificates and hospital records.	?	There is no evidence of selective reporting	L	L	HIGH RISK OF BIAS
Yang et al. 2022	The randomisation sequence was generated by using the website Randomization.com (http://www.randomization.com).<br><br>The research assistant blinded to the allocation order will assign the treatment group using sealed opaque envelopes.	L	Nurses and participants were not feasible to be blinded to the group allocation because of the nature of the intervention.<br>The delivery of intervention components was also checked using<br>the intervention logbooks. To ensure intervention fidelity of motivational interviewing, the second face-to-face educational session was audiotaped. A random 20% sample<br>of each nurse's session was reviewed using Motivational Interviewing<br>Treatment Integrity Coding Manual 4.2.1. Further training for nurses was considered if the intervention was not delivered as protocol.<br>The average proportions of completed components for face-to-face and phone call sessions were 97.1%<br>and 98.8% respectively.<br>The effects of the intervention on the study outcomes were assessed using<br>generalised estimating equation (GEE) model.	L	Losses to follow-up were disclosed and primary and secondary outcomes were analysed following the intention to treat principle.<br>High attrition rate. No statistically significant difference was found between participants who completed the study and those who dropped out for socio-demographic characteristics. However, participants who dropped out had more CHC visits and fewer emergency department visits than those who completed the study.	?	The researcher collecting baseline and follow-up data was blind to participant allocation.<br>Self-reported outcomes (adherence and quality of life) may be impacted by patients' lack of blinding.	?	All prespecified outcomes were reported	L	?	SOME CONCERNS

L: LOW
H: HIGH
?: SOME CONCERNS

SUPPLEMENTARY TABLE 6b. Risk Of Bias of Quasiexperimental studies

Author, Year	PRE-INTERVENTION DOMAINS			AT-INTERVENTION DOMAIN		POST-INTERVENTION DOMAINS					OVERALL RISK OF BIAS
	SUPPORT FOR JUDGEMENT	Bias due to confounding	Bias in selection of participants into the study	SUPPORT FOR JUDGEMENT	Bias in classification of interventions	SUPPORT FOR JUDGEMENT	Bias due to deviations from intended interventions	Bias due to missing data	Bias in measurement of the outcome	Bias in selection of the reported result	
Al-Rashed et al. 2002	To address potential confounding, wards that were similarly managed and patients who met the same criteria were recruited.<br>However, the lack of randomisation may still lead to some confounding factors that were not controlled.	PY	PN	The intervention group received additional pharmaceutical counseling and a medicine reminder card in addition to the standard discharge summary (MIDS). The control group received the standard discharge summary and usual care. The interventions and control were well-defined, and adherence to the intervention protocols was monitored.	N	Patients in both groups received the interventions as planned. Adherence to the intervention was monitored and follow-up visits were conducted.<br><br>There was some attrition (deaths and withdrawals), which might affect the results.<br><br>Outcomes were assessed using structured questionnaires and home visits which were clearly described.<br><br>A wide range of outcomes were reported, including drug knowledge, compliance, GP visits, and readmissions.<br><br>There is no evidence of selective reporting; the study results are reported in detail.	N	PY	PN	N	HIGH RISK OF BIAS
Karapinar-Çarkıt et al. 2019	The study used a prospective interrupted time-series design with multiple pre- and post-intervention measurements and included potential confounders (e.g. age, comorbidities) in the segmented regression model. However, as no external control group was included, residual confounding by time trends, seasonality, or organisational changes cannot be excluded.<br>All admitted internal-medicine patients with at least one chronic medication were invited to participate according to predefined inclusion and exclusion criteria.	PY	PN	Intervention (COACH) clearly distinguished from usual care by study period.	N	Fidelity of the COACH intervention was checked by counting completed checklists for medication reconciliation, counselling, and information transfer. No major deviations from the intended procedures or contamination of usual care were reported. Administrative outcomes were nearly complete, but older and more severely ill patients were less likely to give informed consent, which may have led to an underestimation of rehospitalisation rates. Questionnaire response rates were limited despite follow-up calls.<br><br>The primary outcome (unplanned rehospitalisation within six months) was obtained from hospital records, providing objective data. Secondary outcomes (adherence, attitudes, satisfaction) were based on validated self-report questionnaires (MARS, BMQ).<br><br>A published study protocol was available, and reported outcomes were consistent with prespecified objectives. No evidence of selective reporting.	PN	PY	PN	N	HIGH RISK OF BIAS
Hugtenburg et al. 2009	Self-selection could result in systematic differences between the groups that could affect outcomes. Confounding variables such as the baseline characteristics of the pharmacists or the pharmacies could influence the results.	Y	Y	The intervention was clearly defined as the implementation of the IBOM-1 protocol and included an extensive medication review and drug counselling at the patient's home. Control pharmacies provided usual care.	PN	Some pharmacists did not fully implement the IBOM-1 protocol.<br>High loss to follow-up.<br>The reasons for missing data were balanced between the groups.<br><br>Outcomes such as drug discontinuation, and mortality were objectively measured.<br>Self-reported data on protocol adherence may lead to measurement bias.<br><br>No evidence of selective reporting.	PY	PY	PY	N	HIGH RISK OF BIAS
Leendertse et al. 2013	The study was originally a randomised controlled trial (RCT) but was converted to a controlled open study due to participant non-compliance.<br><br>Participants were selected based on specific high-risk criteria based on old age, use of five or more medicines, non-adherence and type of medication used were included.	Y	PY	The intervention consisted of a patient interview, a review of the pharmacotherapy and the execution and follow-up evaluation of a pharmaceutical care plan. The patient's own pharmacist and GP carried out the intervention and it was documented through care plans.<br>The control group received usual care and was cared for by a GP other than the intervention GP.	PN	The intervention was performed as planned. The implementation rate was high (73%).<br>Data were analysed using mixed-effects models, and analyses were conducted considering the effect of both pharmacists and GPs. The results and bias evaluations were rigorous and blinded.<br>Quality of life and adverse events were measured using standardised tools.<br>No evidence of selective reporting.	N	PY	N	N	HIGH RISK OF BIAS

SUPPLEMENTARY TABLE 6b. Risk Of Bias of Quasiexperimental studies

Author, Year	PRE-INTERVENTION DOMAINS			AT-INTERVENTION DOMAIN		POST-INTERVENTION DOMAINS					OVERALL RISK OF BIAS
	SUPPORT FOR JUDGEMENT	Bias due to confounding	Bias in selection of participants into the study	SUPPORT FOR JUDGEMENT	Bias in classification of interventions	SUPPORT FOR JUDGEMENT	Bias due to deviations from intended interventions	Bias due to missing data	Bias in measurement of the outcome	Bias in selection of the reported result	
Matzke et al. 2018	Both groups were matched using propensity scores to control for confounding factors such as age, sex, race, insurance status, and number of chronic conditions. Residual confounding may still be present.<br><br>Patients were selected for the collaborative care group based on specific criteria and were identified using EMR algorithms and referrals. The usual care group was matched on similar characteristics. The process for selecting patients and the matching procedure are well-documented, which reduces selection bias. However, the use of EMR algorithms and referrals may still introduce some selection bias if not all relevant factors were accounted for.	PY	PY	Intervention: comprehensive medication management by clinical pharmacists and chronic disease state management. Control group received usual care. The intervention and control conditions were clearly defined. However, if the implementation of the intervention was not consistent across all participants or clinics, this could introduce bias.	PY	Some patients in the collaborative care group did not attend scheduled encounters.<br>No detailed information on how deviations were handled or accounted for.<br><br>The article does not specify how missing data were managed or imputed.<br><br>Outcomes were measured using validated methods (EMR data).<br><br>The comprehensive reporting of outcomes and statistical analysis reduces the risk of selective reporting.	PY	PY	PN	PY	HIGH RISK OF BIAS
Moczygemba et al. 2011	The study accounts for confounders such as patient demographics, baseline health status, and other potential confounders through the use of propensity score matching, but some residual confounding may still be present.<br>Patients were selected based on specific criteria such as poorly controlled diabetes or hypertension. Selection was based on clinical criteria before the intervention was known to the participants.	PY	N	The interventions (telephone medication therapy management) and comparator (usual care) were clearly defined.<br>There is no evidence that the intervention status was misclassified.	PY	Intervention delivery and adherence to the protocol were well described.<br><br>Detailed information on missing data is not provided.<br><br>Standardised measures for medication adherence and health-related problems were used.<br>It is unclear if the outcome assessors were blinded to the intervention status.<br><br>It is unclear if multiple analyses were conducted and selectively reported.	PY	PY	PY	PY	HIGH RISK OF BIAS
Moreno et al. 2021	The study employed propensity score matching to adjust for confounding factors, such as age, gender, race-ethnicity, comorbidities, and other relevant variables. However, some residual confounding may still be present.<br><br>Participants were selected based on their diabetes control and use of clinical pharmacists. The selection of the 14 intervention practices and the comparator practices could introduce selection bias if these practices were different in their willingness to adopt new interventions or their patient populations.	PY	PY	The intervention (pharmacist consultation) and the comparator (usual care) were clearly defined. The study used electronic health records to track and classify the intervention and control conditions.	N	The clinical pharmacist consultations were provided according to a standardised protocol.<br>No details on deviations from intended interventions were provided.<br>A substantial sample size and statistical methods were used to address missing data.<br><br>Outcomes were measured using electronic health records for hospitalizations and emergency room visits.<br>The study presents results for both primary outcomes (ER visits and hospitalizations) and uses appropriate statistical methods to analyse the data. There is no evidence of selective reporting.	PY	N	N	N	HIGH RISK OF BIAS

SUPPLEMENTARY TABLE 6b. Risk Of Bias of Quasiexperimental studies

Author, Year	PRE-INTERVENTION DOMAINS			AT-INTERVENTION DOMAIN		POST-INTERVENTION DOMAINS					OVERALL RISK OF BIAS
	SUPPORT FOR JUDGEMENT	Bias due to confounding	Bias in selection of participants into the study	SUPPORT FOR JUDGEMENT	Bias in classification of interventions	SUPPORT FOR JUDGEMENT	Bias due to deviations from intended interventions	Bias due to missing data	Bias in measurement of the outcome	Bias in selection of the reported result	
Odeh et al. 2019	The study acknowledges potential confounders such as patient comorbidities and baseline medication adherence. Propensity score matching was employed to minimize these confounders. Participants were selected from a specific patient group (those discharged from the hospital and receiving 10 or more prescribed medicines).	N	N	The interventions (pharmacist-led telephone follow-up) and control conditions are well-defined.<br>Details on nature and delivery of the interventions were given.	PN	The study protocol ensured that the pharmacist-led intervention was delivered consistently.<br><br>Not significant amount of missing data; however, no details on missing data handling were provided.<br><br>The outcomes, including readmission rates, medication adherence, and patient satisfaction, were measured using standard and validated tools.<br>There is no evidence of selective reporting.	N	PY	N	N	HIGH RISK OF BIAS
Perman et al. 2021	Quasi-experimental design without randomisation. Baseline characteristics (age, sex, comorbidities, frailty) were comparable between groups, and analyses were adjusted for potential confounders. However, residual confounding by unmeasured factors cannot be excluded.<br><br>Inclusion and exclusion criteria were clearly defined and applied consistently. All eligible frail older adults identified by primary care teams were invited to participate. Group allocation depended on geographic area, which had comparable socioeconomic and [illegible]	PY	PN	Intervention clearly defined (structured home-based assessment by social-health counsellor vs usual care).	N	Intervention delivered consistently but variable intensity of follow-up visits; adherence to protocol not quantified.<br>Minimal loss to follow-up; primary outcomes obtained for all. Outcomes (hospitalisations, mortality) from institutional records; assessed by blinded researcher.<br>No study protocol or trial registration was identified. Reported outcomes were consistent with stated objectives, but selective reporting cannot be fully excluded.	PY	PN	N	PN	HIGH RISK OF BIAS
Reidt et al. 2016	Quasi-experimental concurrent comparison (natural experiment); assignment by weekday of discharge. Baseline characteristics between intervention and control groups were comparable (no significant differences reported). However, potential selection bias cannot be excluded due to non-random assignment by discharge day and residual confounding may persist related to timing or clinical workflow. Adjusted for age, sex, race/ethnicity, payor, and Charlson comorbidity.	PY	PY	Intervention (pharmacist review and coordination pre-discharge) vs usual care clearly defined.<br>Group assignment determined by weekday of discharge.	N	10/87 patients declined home visit and received telephone follow-up.<br>Same Nurses/geriatrician managed both groups.<br>Two participants lost to follow-up (2/87).<br>Objective outcome data completed from EHR.<br>All prespecified outcomes (hospitalizations, ED visits) described and reported as stated in Methods; however, no registered or published protocol was available.	PN	PN	PN	PN	HIGH RISK OF BIAS

SUPPLEMENTARY TABLE 6b. Risk Of Bias of Quasiexperimental studies

Author, Year	PRE-INTERVENTION DOMAINS			AT-INTERVENTION DOMAIN		POST-INTERVENTION DOMAINS					OVERALL RISK OF BIAS
	SUPPORT FOR JUDGEMENT	Bias due to confounding	Bias in selection of participants into the study	SUPPORT FOR JUDGEMENT	Bias in classification of interventions	SUPPORT FOR JUDGEMENT	Bias due to deviations from intended interventions	Bias due to missing data	Bias in measurement of the outcome	Bias in selection of the reported result	
Westberg et al. 2014	The study adjusted for relevant confounding variables: sex, age, Charlson Comorbidity Index (CCI), and the number of medications at discharge through group matching.<br>Patients were selected based on specific criteria before the intervention was known.<br>Group matching was performed.<br>To control for the effect of systemwide program and policy changes, 6-month periods of patient hospital discharge were included as a covariate in all analyses.<br>* Subjects recruited for the control group were contacted during normal business hours. Potential subjects being discharged on evening and weekends were not contacted. Differences between these patient groups that may biased the results are unknown.	N	PN	The interventions were well-defined (comprehensive medication management versus standard care)<br><br>Classification of interventions was accurate since the pharmacist interactions were scheduled and recorded.	N	The intervention group received comprehensive medication management as planned and control subjects received standard care.<br><br>There was no indication of significant missing data.<br><br>The analysis included all patients who received the initial comprehensive medication management assessment.<br><br>Outcomes were measured using electronic health records (EHR), reducing subjective bias.<br><br>There is no evidence of selective reporting.<br>Both significant and non-significant results were reported.	N	N	N	N	MODERATE RISK OF BIAS

ROBVIS TOOL
Y: Yes=Critical
PY: Probably Yes= Serious
PN: Probably No=Moderate
N: No= Low
