# Supplementary material for: Interventions to enhance in-home taking medication among older adults with multimorbidity/polypharmacy: a systematic review and meta-analysis
Source: Front Public Health. 2026 Jan 28;13:1701622. doi: 10.3389/fpubh.2025.1701622 (PMC12891206; doi:10.3389/fpubh.2025.1701622)
Supplement: Supplementary file 1 [file Data_Sheet_1.zip › Supplementary Table 7. Primary Outcomes.pdf]

ADHERENCE-MMAS-4/MMAS-8						INTERVENTION				CONTROL				EFFECT SIZE						
Authors/ Year	Design	Outcome		Tool	Time point (months)	Mean score (SD)	%	n	N	Mean score (SD)	%	n	N	OR/ Adjusted Difference/ MD	CI 95% lower limit	CI 95% upper limit	P value	Results	Interpretation	Risk of Bias
Aguiar et al. 2018	RCT	Medication Adherence (Adherence score)		MMAS-4	12	-	-	-	36	-	-	-	37	-	-	-	-		The intervention group demonstrated a significant increase in both the percentage of adherent patients and the average score from the Morisky–Green test compared to	SOME CONCERNS
Campins et al. 2017	RCT	Medication Adherence		MMAS-4	Baseline	-	61.8	-	252	-	60.2	-	251	-	-	-	0.713		While no significant differences were found at baseline, the intervention group showed higher adherence rates at six months.	SOME CONCERNS
					6	-	76.4	-	252	-	64.1	-	251	-	-	-	0.005			
Chrischilles et al. 2014	RCT	Medication Adherence		Modified MMAS-4	Baseline	14.2 (1.8)	-	-	802	14.1 (1.9)	-	-	273	-	-	-	-		The study reported no significant changes in medication adherence between the groups at baseline or at six months.	SOME CONCERNS
					6	13.8 (1.9)	-	-	802	13.9 (1.9)	-	-	273	-	-	-	0.9821			
Del Cura-González et al. 2022	Cluster- RCT	Medication Adherence		MMAS-4	Baseline	-	46.0	137	298	-	35.6	105	295	-	-	-	-		There was no significant change in adherence between the evaluated groups.	HIGH RISK OF BIAS
					12	-	26.8	73	272	-	25.4	71	280	Adjusted Difference: -0.048	-0.65	0.56	0.87			
Jarab et al. 2012	RCT	Medication Adherence		MMAS-4	Baseline	-	36.4	24	66	-	40.3	27	67	-	-	-	0.68		There was a significant decrease in the proportion of non-adherent patients in the intervention group when compared to the control group at 6 months.	HIGH RISK OF BIAS
					6	-	71.4	45	63	-	51.6	33	64	-	-	-	17			
Kouladjian O'Donnell et al. 2021	Cluster-RCT	Medication Adherence		MMAS-4	Baseline	3.5 (0.8)	-	-	63	3.3 (1.2)	-	-	96	-	-0.26	-	-		There were no statistically significant differences between the comparison and intervention groups for medication adherence.	HIGH RISK OF BIAS
					3	3.5 (0.8)	-	-	63	3.4 (1.2)	-	-	96	MD: -0.06	-0.26	0.12	-			
Morales Suárez-Varela et al. 2009	RCT	Medication Adherence		MMAS-4	Baseline	-	6.7	-	89	-	11.8	-	93	-	-	-	-		The percentage improvements in adherence were not statistically significant between groups.	SOME CONCERNS
					2	-	13.5	-	89	-	14.0	-	93	-	-	-	-			
Muth et al. 2018	Cluster-RCT	Medication Adherence		MMAS-4	Baseline	3.7 (0.6)	-	-	250	3.7 (0.8)	-	-	252	-	-		-		At 6 months, the intervention group scored 3.6 compared to 3.8 in the control group, with a significant p-value of 0.044.	LOW RISK OF BIAS
					6	3.6 (0.8)	-	-	237	3.8 (0.5)	-	-	238	MD: -0.1	-0.2	0.0	0.044			
					9	3.7 (0.7)	-	-	231	3.7 (0.6)	-	-	225	MD: 0.0	-0.2	0.1	0.629			
Sáez de la Fuente et al. 2011	RCT	Medication Adherence		MMAS-4	Baseline	-	62.1	-	26	-	83.3	-	24	OR: 0.33	0.1	1.1	0.7		The odds ratio indicated a notable difference in adherence favoring the intervention group at 1-1.6 months.	HIGH RISK OF BIAS
					1-1.6	-	88.5	-	26	-	62.5	-	24	OR: 4.6	1.1	19.8	0.03			
Biswas et al. 2018	RCT	Medication Adherence		MMAS-8	Baseline	2.45	-	-	40	-	2.97	-	35	-	-	-	-		There was a statistical significant change in the Morisky's 8 point Adherence Scale score (p<0.0001) between the supervised and unsupervised group when compared between baseline and end of study [Table/Fig-10]. The impact of supervision on medication adherence was evident.	HIGH RISK OF BIAS
					6	0.7	-	-	40	-	1.81	-	35	-	-	-	-			
					12	0.39	-	-	35	-	01.03	-	31	-	-	-	< 0.001			
Messerli et al. 2016	RCT	Medication Adherence	Low	MMAS-8[1]	2	-	18.9	37	198	-	18.3	37	202	-	-	-	0.817		No significant difference in adherence between the two groups could be observed at 2 months.	HIGH RISK OF BIAS
			Moderate			-	20.8	83	198	-	22.3	89	202	-	-	-				
			High			-	39.4	78	198	-	37.6	76	202	-	-	-				
		Medication Adherence		MMAS-8[1]	7	6.85 (1.226)	-	-	202	6.82 (1.237)	-	-	198	-	-	-	0.817		No significant differences in adherence were observed between the intervention and control groups at 7 months.	
Poorcheraghi et al. 2023	RCT	Medication Adherence	Low	MMAS-8	Baseline	-	52.08	50	96	-	48.96	47	96	-	-	-	0.919		No significant differences in adherence were detected between the intervention and control groups at baseline.	SOME CONCERNS
			Moderate			-	35.42	34	96	-	38.54	37	96	-	-	-				
			High			-	-	-	96	-	12.5	12	96	-	-	-				
		Medication Adherence	Low	MMAS-8	2	-	23.92	22	92	-	43.48	40	92	-	-	-	< 0.001		A significant increase in adherence is noted at 2 months post-intervention.	
			Moderate			-	31.52	29	92	-	40.22	37	92	-	-	-				
			High			-	44.56	41	92	-	16.3	15	92	-	-	-				

%: proportion of adherent patients
n: number of adherent patients
MD: Mean Difference
OR: Odds Ratio
1. Only two questions were used

Indicates a significant improvement in the outcome measure for the intervention group compared to the control group.

Indicates a non-significant effect or no clear difference in the outcome measure between the intervention and control groups.

Indicates a negative effect, meaning a worsening of the outcome measure in the intervention group compared to the control group.

Among the nine studies that measured medication adherence using the MMAS-4, four reported statistically significant improvements in adherence levels.
Aguiar et al. reported a significant increase in the percentage of adherent patients in the intervention group, along with a higher average adherence score compared to the control group at 12 months. Similarly, Sáez de la Fuente et al. indicated a notable difference in adherence shortly after the intervention and, Campins et al. and jarab et al. reported higher adherence rates in the intervention group at 6 months.
In contrast, Muth et al. showed a decrease in adherence scores among the intervention participants at the six-month follow-up.
The remaining studies found no significant differences in medication adherence between intervention and control groups. Chrischilles et al., Del Cura-González et al., and Kouladjian O'Donnell et al., reported no significant changes in adherence rates. Additionally, Morales Suárez-Varela et al. found no statistically significant differences in adherence improvements between groups.
In the three studies that assessed medication adherence using the MMAS-8, significant improvements in adherence scores were observed in the studies by Biswas et al. and Poorcheraghi et al. In contrast, Messerli et al. found no significant differences between the intervention and control groups.

ADHERENCE-OBJECTIVE METHODS					INTERVENTION				CONTROL				EFFECT SIZE						
**Authors/ Year**	**Design**	**Outcome**	**Tool**	**Time point (months)**	**Mean % (SD)**	**%**	**n**	**N**	**Mean % (SD)**	**%**	**n**	**N**	**OR/ Diff. proportion/ MD/ β**	**CI 95% lower limit**	**CI 95% upper limit**	**P value**	**Results**	**Interpretation**	**Risk of Bias**
Insel et al. 2012	RCT	Medication Adherence	MEMS	Baseline	57.4(29.8)	-	-	58	67.8(28.5)	-	-	58	-	-	-	-		The intervention group showed a significant increase in adherence from baseline to the end of nurse visits (p < 0.001), although a decrease was noted during the extended monitoring phase. The control group exhibited no significant change.	HIGH RISK OF BIAS
				1.25	77.8(24.4)	-	-	58	68.3(30.2)	-	-	58	-	-	-	Intervention: >0.001 Control: 0.90			
				5	59.0 (32.7)	-	-	58	61.1(29.9)	-	-	58	-	-	-	Intervention: <0.001 Control: 0.02			
Jerant et al. 2009	RCT	Medication Adherence	Pill count	6	89.0(18.0)	-	-	139	93.0(13.0)	-	-	138	-	-	-	-		There were no significant differences between groups at 6 and 12 months.	HIGH RISK OF BIAS
				12	93.0 (12.0)	-	-	139	91.0(15.0)	-	-	138	-	-	-	-			
Lee et al. 2006	RCT	Medication Adherence	Pill count	Baseline (Run-in-2 months)	61.4(13.0)	-	-	83	61.1(14.1)	-	-	76	-	-	-	-		A substantial increase in adherence was observed at 8 months, maintaining high levels at 14 months	LOW RISK OF BIAS
				8	96.9 (5.2)	-	-	159	-	-	-	-	-	-	-	<0.001			
				14	95.5 (7.7)	-	-	83	69.1 (16.4)	-	-	76	-	-	-				
Olesen et al. 2014	RCT	Medication Non-adherence	Pill count	12	-	89.0	225	253	-	238	90.0	264	OR: 1.00	-	-	-		No significant differences in non-adherence rates between intervention and control groups over 12 months (11% vs. 10%).	HIGH RISK OF BIAS
Al-Rashed et al. 2002	Quasiexperimental	Medication Adherence Compliant % of total items	Pill count/ Home medicine stocks-refill records	15-22 days postdischarge	-	48.4	-	340 (items)	-	15.9	-	331 (items)	-	-	-	< 0.001		A significant improvement in adherence post-intervention was found.	HIGH RISK OF BIAS
				3	-	70.0	-	342 (items)	-	15.8	-	328 (items)	-	-	-				
Hugtenburg et al. 2009	Quasiexperimental	Medication Adherence	Medication record review	9	-	64.0	-	336	-	58.0	-	379	-	-	-	-		Over a 9-month period, a few more patients of pharmacies in the intervention group ceased using the drugs that were first prescribed in hospital for the chronic disease for which they had been treated. The difference, however, was not significant.	HIGH RISK OF BIAS
Messerli et al. 2016	RCT	Medication Adherence	MPR	7	88.3(19.03)	-	-	493 (therapies)	87.5 (20.75)	-	-	527 (therapies)	-	-	-	0.811		Both groups showed similar MPR scores with no significant improvement in the intervention group.	HIGH RISK OF BIAS
			DPPR	7	88.0(13.31)	-	-	146	87.5(20.75)	-	-	147	-	-	-	0.906		Both groups had similar DPPR scores, the intervention did not result in a significant improvement in adherence when compared to the control group.	
Moczygemba et al. 2011	Quasiexperimental	Medication Adherence	MPR/ Refill records	Baseline	67.0(16.0)	-	-	60	67.0(17.0)	-	-	60	-	-	-	0.79		There was no significant change in MPR for the intervention group from baseline to follow-up. The control group showed a slight increase in MPR, but this change was also not significant. The regression model indicated that MTM did not have a statistically significant impact on medication adherence when controlling for other factors.	HIGH RISK OF BIAS
				6	68.0(15.0)	-	-	60	70.0(17.0)	-	-	60	-	-	-				
Muth et al. 2018	RCT	Medication Adherence **Dose score**	Discrepancies between medicines actually taken and medicines prescribed	Baseline	-	54.0	134	248	-	49.8	125	251	-	-	-	-		There were no statistically significant differences in medication adherence between the intervention and control groups across all measured scores at any of the time points evaluated (baseline, 6 months, and 9 months).	LOW RISK OF BIAS
				6	-	57.6	136	236	-	54.5	128	235	OR: 1.1	0.7	1.6	0.756			
				9	-	63.3	145	229	-	54.5	121	222	OR: 1.4	0.9	2.0	0.119			
		Medication Adherence **Drug score**		Baseline	-	34.8	87	250	-	40.2	101	251	-	-	-	-			
				6	-	32.9	78	237	-	42.6	101	237	OR: 0.7	0.5	1.0	0.051			
				9	-	36.8	85	231	-	39.3	88	224	OR: 0.9	0.6	1.4	0.736			
		Medication Adherence **Regimen score**		Baseline	-	52.6	131	249	-	49.4	124	251	-	-	-	-			
				6	-	56.8	134	236	-	49.8	117	235	OR: 1.3	0.8	2.0	0.297			
				9	-	59.8	137	229	-	51.4	114	222	OR: 1.4	0.9	2.1	0.148			
Heaton et al. 2019	RCT	Primary Medication Non-Adherence	Refill Records	1	8.3(20.8)	-	-	213	8.9(21.8)	-	-	187	-	-	-	0.87		Primary medication non-adherence showed no significant difference between groups.	HIGH RISK OF BIAS
		Secondary Medication Non-Adherence	PDC	6	-	60.5	-	213	-	37.5	-	187	-	-	-	0.04		Secondary non-adherence revealed a significant difference with 60.5% adherence in the intervention group compared to 37.5% in the control group.	

OR: Odds Ratio

Among the ten studies using objective measures of medication adherence, several reported significant improvements in adherence within the intervention groups.

OR: Odds Ratio [illegible]
MD: Mean Difference
β: Group effect
Diff. proportion: Difference in proportion between groups

Indicates a significant improvement in the outcome measure for the intervention group compared to the control group.

Indicates a non-significant effect or no clear difference in the outcome measure between the intervention and control groups.

Indicates a negative effect, meaning a worsening of the outcome measure in the intervention group compared to the control group.

[illegible]
Insel et al., using the MEMS, observed a significant increase in adherence during nursing visits, although adherence decreased during prolonged follow-up. Similarly, Al-Rashed et al. using a combination of tablet counts and home medication stock records, observed a substantial improvement in adherence shortly after the intervention, with a significant increase in the percentage of adherers in the intervention group compared to the control. Lee et al., who also used tablet counts, observed a substantial increase in adherence at eight months, and participants in the intervention group maintained high levels of adherence over the 14-month follow-up period. Similarly, Heaton et al. using PDC reported significantly higher proportions of optimal adherence in the intervention group compared to the control group at six months.

In contrast, some studies reported no significant differences in adherence between the groups. Jerant et al., Olesen et al. and Messerli et al. using pill counts and medication possession ratios (MPRs) found comparable levels of adherence between the intervention and control groups over time. Moczygemba et al., using MPRs and refill records, also found no significant changes from baseline to follow-up, and both groups showed similar adherence trends.

Hugtenburg et al., using medication record reviews, found that although a few more patients in the intervention group stopped using medications prescribed in hospital for chronic conditions over a nine-month period, this difference was not statistically significant. Similarly, Muth et al., using discrepancy scores between medications taken and prescribed, found no significant differences in adherence between the intervention and control groups at various time points (baseline, six months and nine months).

ADHERENCE-OTHER TOOLS					INTERVENTION				CONTROL				EFFECT SIZE						
**Authors/ Year**	**Design**	**Outcome**	**Tool**	**Time point (months)**	**Mean score (SD)**	**%**	**n**	**N**	**Mean score (SD)**	**%**	**n**	**N**	**OR/ Diff. proportion/ MD/ β**	**CI 95% lower limit**	**CI 95% upper limit**	**P value**	**Results**	**Interpretation**	**Risk of Bias**
Bernsten et al. 2001	RCT	Medication Adherence	Self-reported	Baseline	-	33.9	-	1290	-	38.6	-	1164	-	-	-	-		While no significant differences in overall adherence rates were observed between intervention and control groups across the study duration, a significantly higher proportion of initially noncompliant patients in the intervention group transitioned to compliant status by 18 months (15.2% vs. 12.2%, p = 0.028).	HIGH RISK OF BIAS
				6	-	38.5	-	1024	-	36.6	-	953	-	-	-	-			
				12	-	43.8	-	863	-	37.3	-	764	-	-	-	-			
				18	-	38.2	-	704	-	39.4	-	636	-	-	-	-			
Biswas et al. 2018	RCT	Medication Adherence	Self-reported	Baseline	-	-	16	40	-	-	11	35	-	-	-	0.023		The study reported a statistically significant improvement in the number of patients without missed doses in the supervised intervention group compared to the control group.	HIGH RISK OF BIAS
				12	-	-	18	35	-	-	10	31	-	-	-				
Wu et al. 2006	RCT	Medication Adherence	Pill count/ Computerised information	Baseline	-	12.0	14	117	-	15.0	18	119	-	-	-	-		Participants in the intervention group showed significantly higher adherence rates at 24 months compared to controls.	HIGH RISK OF BIAS
				24	-	81.0	95	117	-	58.0	69	119	-	-	-	0.038			
Taylor et al. 2003	RCT	Medication Adherence¹	Self-reported/ Refill records **MIXED METHODS**	Baseline	84.9 (6.7)	-	-	33	88.9 (5.8)	-	-	36	-	-	-	0.728		Medication compliance scores improved in the intervention group but not in the control group, but they did not differ significantly between groups.	HIGH RISK OF BIAS
				12	100	-	-	33	88.9 (6.3)	-		36	-	-	-	115			
Martínez-Mardones et al. 2023	Cluster-RCT	Medication Adherence	Chilean MAQ	Baseline	-	46.4	81	174	-	32.1	48	150	-	-	-	-		A significant difference in medication adherence was noted at the final visit, with the intervention group showing higher adherence.	HIGH RISK OF BIAS
				12	-			174	-			150	OR: 6.60	1.36	31.9	0.022			
Shim et al. 2018	RCT	Medication Adherence	MALMAS scale	Baseline	-	35.6	26	73	-	32.9	26	79	-	-	-	-		Medication adherence among participants in the intervention group was significantly higher than that of the control group .	SOME CONCERNS
				6	-	69.9	51	73	-	31.6	25	79	-	-	-	<0.001			
Messerli et al. 2016	RCT	Medication Adherence	VASAD	Baseline	0.962 (0.0862)	-	-	211	0.968 (0.0705)	-	-	232	-	-	-			No significant differences in adherence between two groups at baseline, 4 or 7 months.	HIGH RISK OF BIAS
				4	0.985 (0.0556)	-	-	198	0.978 (0.0764)	-	-	202	-	-	-	0.400			
				7	0.955 (0.1208)	-	-	178	0.963 (0.0951)	-	-	186	-	-	-	0.338			
Nazareth et al. 2001	RCT	Medication Adherence	The Prescribed Medicine Interview	Baseline	0.80 (0.31)	-	-	123	0.77 (0.30)	-	-	122	-	-	-	-		No significant differences in adherence were observed at baseline, 3, 6 months.	HIGH RISK OF BIAS
				3	0.75 (0.30)	-	-	91	0.75 (0.28)	-	-	92	-	-	-	-			
				6	0.78 (0.30)	-	-	90	0.78 (0.30)	-	-	88	-	-	-	-			
Syafhan et al. 2021	RCT	Medication Adherence	MARS	Baseline	-	-	-	181	-	-	-	175	-	-	-	-		At both baseline and the end of the study, median MARS scores were of 24, indicating high self-reported medication adherence among patients in both the intervention and control groups.	HIGH RISK OF BIAS
				6	-	-	-	119	-	-	-	130	-	-	-	-			
Yang et al. 2022	RCT	Medication Adherence	MARS-5	Baseline	15.43 (2.80)	-	-	67	15.70 (2.84)	-	-	69	-	-	-	-		No significant differences in adherence scores were observed.	SOME CONCERNS
				3	17.88 (2.41)	-	-	67	16.98 (2.70)	-	-	69	β: -0.03	-1.12	01.06	0.955			
Karapinar-Çarkıt et al. 2019	Quasiexperimental	Medication Adherence	MARS	Baseline	23.38 (2.8)			104	23.40 (2.4)			106				0.95		No significant differences between groups were observed.	HIGH RISK OF BIAS
				1	23.88 (2.2)			62	23.57 (2.1)			66				0.42			
Odeh et al. 2019	Quasiexperimental	Medication Adherence	MARS	Baseline	-	-	-	83	-	-	-	83	-	-	-	-		There was a statistically significant improvement in self-reported adherence, with a mean adherence scale difference of 1.4 (p < 0.001) pre- and post-intervention.	HIGH RISK OF BIAS
				3	-	-	-	83	-	-	-	83	-	-	-	-			

OR: Odds Ratio
MD: Mean Difference
β: Group effect
Diff. proportion: Difference in proportion between groups

Among the twelve studies that used other self-reported tools to measure medication adherence, six found significant improvements in adherence among the intervention groups.
Bernsten et al., using self-reported measures, found that although overall adherence rates did not differ significantly between intervention and control groups at various points in time, a statistically significant proportion of initially non-adherent patients in the intervention group became adherent at 18 months.
Biswas et al., using non-specific self-reported adherence measures, reported a significant increase in the number of patients in the intervention group who did not miss any doses compared to the control group at 12 months. Wu et al., combining self-reports, pill counts and computerised information, observed significantly higher adherence rates in the intervention group at 24 months compared to controls. Martinez-Mardones et al., using the Chilean MAQ, found that the intervention group showed significantly higher adherence rates at final follow-up. Similarly, Shim et al., using the MALMAS scale, reported a significant increase in adherence in the intervention group after six months, with adherence rates almost twice as high as the control group. Odeh et al., using the MARS scale, also found a statistically significant improvement in self-reported adherence, with a mean increase in the adherence scale after the intervention.

In contrast, other studies observed no significant differences between intervention and control groups. Messerli et al., using the VASAD scale, and Nazareth et al., with the Prescribed Medicine Interview, both reported no significant differences in adherence at various follow-up points. Similarly, Yang et al., Syafhan et al., and Karapinar-Çarkıt et al. using versions of the MARS, and Taylor et al. using a combination of self-reported methods and refill records, found no significant changes in adherence scores between the groups.

Indicates a significant improvement in the outcome measure for the intervention group compared to the control group.

Indicates a non-significant effect or no clear difference in the outcome measure between the intervention and control groups.

Indicates a negative effect, meaning a worsening of the outcome measure in the intervention group compared to the control group.

NUMBER OR HOSPITALISATIONS					INTERVENTION			CONTROL			Effect Size						
Authors/ Year	Design	Outcome	Tool	Time point (months)	Mean (SD)/ Median [IQR]	n	N	Mean (SD)/ Median [IQR]	n	N	Adjusted Difference/RR/β/HR/DID	CI 95% lower limit	CI 95% upper limit	P value	Results	Interpretation	Risk of Bias
Del Cura-González et al. 2022	Cluster-RCT	Hospitalisations [1]	-	6	1 [1-2]	-	287	1 [1-1]	-	287	Adjusted Difference: -0.098	-0.644	0.449	0.726		No significant difference in number of hospitalisations was observed between intervention and control groups at both 6 and 12 months.	HIGH RISK OF BIAS
				12	1 [0-1]	-	272	1 [1-2]	-	280	Adjusted Difference: -0.14	-0.57	0.30	0.52			
Jerant et al. 2009	RCT	Hospitalisations	Hospital Episode Statistics	12	-	-	139	-	-	138	-	-	-	-		No significant difference in number of hospitalisations was observed between intervention and control groups.	HIGH RISK OF BIAS
Lenaghan et al. 2007	RCT	Hospitalisations [2]	Hospital Episode Statistics	6	-	21	69	-	20	67	RR: 0.92	0.50	1.70	0.80		A non-significant reduction in hospitalisations of 8% was observed.	SOME CONCERNS
		Hospitalisations [3]	Records of County Council	12	1.7	-	75	2.7	-	66	-	-	-	-		No significant differences were found; however, there was a general trend towards lower hospitalisation in the intervention group.	HIGH RISK OF BIAS
Lenander et al. 2014	RCT	Hospitalisations [3]	Records of County Council	12	1.7	-	75	2.7	-	66	-	-	-	-		No significant differences were found; however, there was a general trend towards lower hospitalisation in the intervention group.	HIGH RISK OF BIAS
Muth et al. 2018	RCT	Hospitalisations [4]	-	6	1.4 (0.5)	-	34	1.4 (0.7)	-	45	RR: 1.2	0.6	2.3	0.646		Number of hospitalisations were comparable between both groups, no significant differences were found.	LOW RISK OF BIAS
				9	1.3 (0.6)	-	28	1.2 (0.4)	-	25	RR: 1.0	0.3	3.1	0.949			
Syafhan et al. 2021	RCT	Hospitalisations [5]	Electronic record system	Baseline [7]	0.2(0.51)	-	169	0.3 (0.67)		161						No statistically significant differences were found between the intervention and control groups. However, the intervention group showed a significant reduction in unplanned hospitalisations when comparing the 6 months post-intervention to the 6 months pre-intervention ($p < 0.05$), while the control group exhibited a smaller, non-significant reduction.	HIGH RISK OF BIAS
				6	0.1 (0.40)	-	169	0.2 (0.58)	-	161	-	-	-	-			
Yang et al. 2022	RCT	Hospitalisations [3]	Self-report questionnaire	3	0.51 (0.66)	-	67	0.33 (0.56)	-	69	β: 0.03	-0.29	0.35	0.864		The group effect was not statistically significant.	SOME CONCERNS
Leendertse et al. 2013	Quasiexperimental	Hospitalisations [6]	Medical History	12	-	6	364	-	10	310	HR: 0.50	0.12	1.59	0.20		No significant differences between groups	HIGH RISK OF BIAS
Matzke et al. 2018	Quasiexperimental	Hospitalisations	Electronic Medical Record	Baseline [8]	-	1,673	1,969	-	355	1,969	-	-	-	-		The reduction in hospitalizations was significantly higher in the intervention group (p>0.0001)	HIGH RISK OF BIAS
				12	-	1,283	1,969	-	324	1,969	-	-	-	-			
Moreno et al. 2021	Quasiexperimental	Hospitalisations	Electronic Health Record	Baseline [8]	0.066 (0.0067)*	-	648	0.045(0.0022)*	-	1,944	-	-	-	-		Predicted reduction in hospitalisations in the intervention group was not statistically significant.	HIGH RISK OF BIAS
				12	0.057(0.0044)*	-	648	0.044(0.0021)*	-	1,944	DID: 0.003(0.0076)*	-	-	0.674			
Perman et al. 2021	Quasiexperimental	Hospitalisations [9]	Electronic health records	12	-	-	121	-	-	121	HR = 0.503	0.340–	0.746	0.001		After adjustment, the intervention was associated with a reduced hazard of first hospitalisation	HIGH RISK OF BIAS

RR:Relative Risk
DID (Difference-in-Differences)
HR: Hazard Ratio
β: Group effect
1.Unplanned and/or number of hospitalisations
2.Non-elective hospital admissions
3.Hospital admissions
4.All-cause Hospitalisation
5.Unplanned hospitalisations
6.Medication-related hospital admissions
7.6 months pre-study
8.12 months pre-intervention
9. Time to first hospital admission
* Expressed as monthly rates (predicted hospitalisations)

Of the ten studies that reported on the total number of hospitalisations, two found statistically significant differences between the intervention and control groups, while some trends were observed in others.

Del Cura-Gonzalez et al. used an unspecified tool and reported no significant differences in hospitalisations at both 6 and 12 months. Similarly, Jerant et al., using hospital episode statistics, found no significant differences in hospitalisation rates between the groups. Lenaghan et al., also using hospital episode statistics, observed a non-significant 8% reduction in hospitalisations, while Lenander et al., using county council records, observed an overall trend towards a reduction in hospitalisations in the intervention group at twelve months, although this was not statistically significant.

Muth et al., using an unspecified tool, found comparable hospitalisation rates between the two groups, with no significant differences at six and nine months. Syafhan et al., obtaining data from the electronic record system, found no statistically significant differences overall, but reported a significant reduction in unplanned hospitalisations in the intervention group when comparing six months post-intervention with six months pre-intervention. Similarly, Matzke et al. reported a significant reduction in hospitalisations in the intervention group based on electronic health record data.
One study, Perman et al., reported time to first hospitalisation using electronic health records and found a significantly lower hazard of first hospital admission in the intervention group.

Indicates a significant improvement in the outcome measure for the intervention group compared to the control group.

Indicates a non-significant effect or no clear difference in the outcome measure between the intervention and control groups.

Indicates a negative effect, meaning a worsening of the outcome measure in the intervention group compared to the control group.

PATIENTS WITH ONE OR MORE HOSPITALISATIONS					INTERVENTION		CONTROL		Effect Size						
**Authors/ Year**	**Design**	**Outcome**	**Tool**	**Time point (months)**	**%**	**N**	**%**	**N**	**DID/ OR**	**CI 95% lower limit**	**CI 95% upper limit**	**P value**	**Results**	**Interpretation**	**Risk of Bias**
Bernsten et al. 2001	RCT	Hospitalisations [1]	Self-report tool	Baseline	41.7	1290	41.3	1164	-	-	-	-		A lower proportion of intervention patients reported one or more hospitalisations compared with control patients, but the difference was not statistically significant.	HIGH RISK OF BIAS
				18	35.6	704	40.4	636	-	-	-	>0.05			
Campins et al. 2017	RCT	Hospitalisations	Electronic primary care clinical histories	3	7.0	245	8.0	248	-	-	-	0.672		No significant difference in hospitalisation rates was observed between the intervention and control groups throughout the follow-up period.	SOME CONCERNS
				6	13.5	199	11.6	173	-	-	-	0.530			
				12	23.3	242	25.2	246	-	-	-	0.616			
Herrinton et al. 2023	RCT	Hospitalisations	Electronic Health Records	6	-	1237	-	1233	DID: 1.6	-1.9	5.1	0.37		No significant difference was found in hospitalisation rates between intervention and control groups at 6 months.	LOW RISK OF BIAS
Jarab et al. 2012	RCT	Hospitalisations [2]	Electronic Medical Record	Baseline[3]	9.1	66	11.9	67	-	-	-	0.77		A significant difference was found in hospitalisation rates between intervention and control groups at 6 months.	HIGH RISK OF BIAS
				6	4.5	66	16.4	67	-	-	-	0.031			
Olesen et al. 2014	RCT	Hospitalisations [3]	E-Health Portal	24	30.0	253	28.0	264	OR: 1.14	0.78	1.67	-		Pharmaceutical care produced no significant effect on the percentages of patients with at least one hospitalisation.	HIGH RISK OF BIAS
Reidt et al. 2016	Quasiexperimental	Hospitalisations	Electronic Health Record	1	9.2	85	19.6	189	OR: 0.47	0.21	01.08	-		Lower odds of hospitalisation in the intervention group, although the difference did not reach statistical significance.	HIGH RISK OF BIAS

OR: Odds Ratio
DID: Difference-in-Differences
1.Excluding Emergency Room visits
2.Hospitalisations for acute exacerbation
3.Inpatient visits

Of the six studies that reported on patients with one or more hospitalisations, only one found a statistically significant difference between the intervention and control groups, although several showed non-significant trends. Bernsten et al. used self-reported data and noted that a lower proportion of intervention patients had one or more hospitalisations compared to control patients, though this difference was not statistically significant. Campins et al., using data from electronic primary care records, also found no significant differences in hospitalisation rates at three, six, or twelve months.

Jarab et al., using data from electronic medical records, found significantly fewer hospitalisations in the intervention group at six months compared with the control group. Reidt et al., similarly reported lower odds of hospitalisation in the intervention group, although this difference did not reach statistical significance.

In contrast, Olesen et al. reported that pharmaceutical care produced no significant effect on hospitalisation rates, but there was a trend suggesting higher rates in the intervention group at 24 months. Additionally, Herrinton et al. observed a trend toward increased hospitalisations in the intervention group at six months, although this difference was not statistically significant. Both Olesen et al. and Herrinton et al. collected data from electronic health records.

Indicates a significant improvement in the outcome measure for the intervention group compared to the control group.

Indicates a non-significant effect or no clear difference in the outcome measure between the intervention and control groups.

Indicates a negative effect, meaning a worsening of the outcome measure in the intervention group compared to the control group.

NUMBER OF READMISIONS					INTERVENTION		CONTROL		Effect size						
**Authors/ Year**	**Design**	**Outcome**	**Tool**	**Time point (months)**	**n**	**N**	**n**	**N**	**Rate Ratio/ OR**	**CI 95% lower limit**	**CI 95% upper limit**	**P value**	**Results**	**Interpretation**	**Risk of Bias**
Bolas et al. 2004	RCT	Number of Readmissions	-	3	-	81	-	81	-	-	-	-		"There was no significant difference (P 0.05) in the number of readmissions between groups . An incidental finding was that there was a significant correlation ($P < 0.02$) between increased number of drugs on discharge and the readmission rates within 3 months"	HIGH RISK OF BIAS
Holland et al. 2005	RCT	Number of Readmissions [1]	Hospital episode statistics	6	234	415	178	414	Rate Ratio: 1.30	01.07	1.58	0.009		Intervention group had significantly higher readmissions.	HIGH RISK OF BIAS
Sáez de la Fuente et al. 2011	RCT	Number of Readmissions	Patient Interview/ Medical History	1-1.6	5	26	7	24	OR: 0.7	0.2	2.7	0.66		No significant difference in readmissions between groups.	HIGH RISK OF BIAS

n: number of readmissions
1.Emergency readmissions

Indicates a significant improvement in the outcome measure for the intervention group compared to the control group.

Indicates a non-significant effect or no clear difference in the outcome measure between the intervention and control groups.

Indicates a negative effect, meaning a worsening of the outcome measure in the intervention group compared to the control group.

Of the three studies that reported on the total number of readmissions, one found significant differences and two did not.
Holland et al. collected data from hospital episode statistics and reported that the intervention group had significantly higher readmission rates.
In contrast, both Bolas et al. and Sáez de la Fuente et al. found no significant difference in readmission rates between the intervention and control groups. Bolas et al., using data from an unspecified source, observed that, although there were no significant differences, they identified a significant correlation between a higher number of medications at discharge and readmission rates within three months. Similarly, Sáez de la Fuente et al., collecting data from medical records and patient interviews, also reported no significant differences in readmissions between groups.

PATIENTS WITH ONE OR MORE READMISSIONS					INTERVENTION		CONTROL		Effect Size						
**Authors/ Year**	**Design**	**Outcome**	**Tool**	**Time point (months)**	**n**	**N**	**n**	**N**	**OR/Diff. Proportion**	**CI 95% lower limit**	**CI 95% upper limit**	**P value**	**Interpretation**	**Results**	**Risk of Bias**
Heaton et al. 2019	RCT	Readmissions	Electronic Health Records	1	24	213	20	187	-	-	-	0.49	No significant differences in the proportion of patients readmitted were found at one month between the intervention and control groups, with both groups exhibiting stable readmission rates		HIGH RISK OF BIAS
Lembeck et al. 2019	RCT	Readmissions	National Health Service Register	1	80	270	70	267	OR: 1.18	0.81	1.73	0.38	No significant difference was found in propotion of patients readmitted at six months.		SOME CONCERNS
				6	150	270	144	267	OR: 1.07	0.75	1.51	0.71			
Nazareth et al. 2001	RCT	Readmissions	Patient administration system	3	64	164	69	176	Diff. Proportion: 0.18	-10.6%	10.2%	-	No observed difference in propotion of patients readmitted between groups at 3 and 6 months.		HIGH RISK OF BIAS
				6	38	136	43	151	Diff. Proportion: 0.18	-11.0%	9.9%	-			
Poorcheraghi et al. 2023	RCT	Readmissions[1]	Self report / Medical records	2	17	92	43	92	-	-	-	<0.001	Significant reduction in propotion of patients readmitted due to disease exacerbation and medication errors in the intervention group.		SOME CONCERNS
		Readmissions[2]	Self report/ Medical records	2	9	92	22	92	-	-	-	0.017			
Sánchez Ulayar et al. 2011	RCT	Readmissions	Hospital's computer records	1	3	41	10	41	-	-	-	<0.05	Significant decrease in propotion of patients readmitted in the intervention group compared to control at two months.		HIGH RISK OF BIAS
				2	3	41	13	41	-	-	-	<0.01			
Van der Heijden et al. 2019	Cluster-RCT	Readmissions	Self-report	6	26	56	14	67	-	-	-	<0.05	Significant reduction in propotion of patients readmitted in the intervention group at six months.		HIGH RISK OF BIAS
Al-Rashed et al. 2002	Quasiexperimental	Readmissions	-	3	3	43	15	40	OR: 0.57	0.36	0.90	<0.05	Significant reduction in propotion of patients readmitted in the intervention group at three months.		HIGH RISK OF BIAS
Karapinar-Çarkıt et al. 2019	Quasiexperimental	Readmissions[3]	Hospital information systems	6	121	365	93	341	-	-	-	>0.05	No significant differences between groups. Both in unadjusted and adjusted linear regression models the results remained non-significant.		HIGH RISK OF BIAS
Odeh et al. 2019	Quasiexperimental	Readmissions	Hospital's computer records	1	38	211	59	211	OR: 0.53	0.36	0.79	<0.001	Significant reduction in propotion of patients readmitted at one and three months in the intervention group.		HIGH RISK OF BIAS
				3	71	211	103	211	-	-	-	0.021			
Westberg et al. 2014	Quasiexperimental	Readmissions	Electronic Health Records	1	4	134	15	268	OR: 0.510	0.164	1.584	-	No significant intervention/control difference was found when evaluating propotion of patients readmitted.		MODERATE RISK OF BIAS
				2	10	134	29	265	OR: 0.678	0.318	1.449	-			
				6	30	134	64	262	OR: 0.958	0.580	1.582	-			

Indicates a significant improvement in the outcome measure for the intervention group compared to the control group.

Indicates a non-significant effect or no clear difference in the outcome measure between the intervention and control groups.

Indicates a negative effect, meaning a worsening of the outcome measure in the intervention group compared to the control group.

1.Readmissions due to disease aggravation
2.Readmissions due to error in medication consumption
3.Unplanned rehospitalisations
n: number of patients with event
OR: Odds Ratio
Diff. proportion: Difference in proportion between groups

Of the ten studies reporting patient readmissions, five found significant differences in readmission rates. Poorcheraghi et al. reported a statistically significant reduction in readmission rates related to disease exacerbation and medication errors, measured by self-reported questionnaires, in the intervention group. Sánchez Ulayar et al. used hospital computer records and documented significant decreases in readmission rates in the intervention group compared to controls at two months. Similarly, Van der Heijden et al. used self-reported measures and found a significant reduction in readmission rates at six months.

In contrast, Heaton et al. used electronic health records and found no significant difference in the proportion of patients readmitted between the intervention and control groups at one month, with both groups showing stable readmission rates. Similarly, Lembeck et al. with data from the National Health Service Register reported no significant differences in readmission rates at six months and Karapinar-Çarkıt et al., based on data from the hospital information systems, found no significant differences in unplanned rehospitalisations at 6 months. Nazareth et al., using a patient management system, also observed no difference in readmission rates at three and six months, supporting the conclusion that the intervention did not have a significant impact on reducing readmissions. Finally, Westberg et al. found no significant difference between intervention and control in readmission rates when using Electronic Health Records.

EMERGENCY DEPARTMENT VISITS					INTERVENTION			CONTROL			Effect Size						
Authors/ Year	Design	Outcome	Tool	Time point (months)	Mean (SD)/ Median [IQR]	n	N	Mean (SD)	n	N	Adjusted Diff. /ATET/IRR/OR	CI 95% lower limit	CI 95% upper limit	p value	Results	Interpretation	Risk of Bias
Campins et al. 2017	RCT	ED visits	Electronic primary care clinical histories	3	0.27 (0.94)	-	245	0.22 (0.53)	-	248	-	-	-	0.726		No statistically significant differences between groups were found.	SOME CONCERNS
				6	0.47 (1.02)	-	199	0.43 (0.81)	-	173	-	-	-	0.985			
				12	0.9 (1.5)	-	242	1.1 (1.5)	-	246	-	-	-	0.061			
Del Cura González et al. 2022	Cluster-RCT	ED visits	-	6	1 [1-2]	-	287	1 [1-2]	-	287	Adjusted Diff. : 0.227	-0.057	0.512	0.118		The adjusted difference suggests a minor trend towards fewer visits in the intervention group, although these differences were not statistically significant.	SOME CONCERNS
				12	1[1-3]	-	272	1 [1-2]	-	280	Adjusted Diff.: 0.18	-0.06	0.41	0.14			
Mc Carthy et al. 2022	RCT	ED visits	Electronic Medical Record	6	0.46 (1.01)	-	188	0.33 (0.85)	-	171	IRR: 1.31	0.699	2.477	0.394		The incident rate ratio (IRR) of 1.31 suggests that the intervention group experienced more ED visits compared to the control group, but this was not statistically significant (p = 0.394).	SOME CONCERNS
Sáez de la Fuente et al. 2011	RCT	ED visits	Patient Interview/ Medical History	1-1.6	-	-	26	-	-	24	OR: 0.8	0.2	2.6	0.74		An odds ratio of 0.8 indicates a potential reduction in ED visits for the intervention group, although it was not significant.	HIGH RISK OF BIAS
Syafhan et al. 2021	RCT	ED visits	Electronic record system at Gp practice	6	0.2 (0.4)	-	169	0.4 (0.8)	-	161	-	-		0.326		No statistically significant differences in ED visits were found between groups	SOME CONCERNS
Yang et al. 2006	RCT	ED visits	Self-report questionnaire	3	0.45 (0.60)	-	67	0.59 (0.54)	-	69	-	-	-	0.251		No statistically significant differences in ED visits were found between groups	SOME CONCERNS
Karapinar-Çarkıt et al. 2019	Quasiexperimental	ED visits	Hospital information systems	6	-	54	365	-	62	341	-	-	-	>0.05		Fewer emergency department visits among patients in the intervention group compared with those in the control group, although the difference was not statistically significant.	HIGH RISK OF BIAS
Matzke et al. 2018	Quasiexperimental	ED visits	Electronic Medical Record	12	-	2021	1969	-	437	1969	-	-	-	-		A small increase of 1.3% in ED visits was noted in the intervention group over 12 months, with no significant change compared to the control group.	HIGH RISK OF BIAS
Moreno et al. 2021	Quasiexperimental	ED visits	Electronic Health Record	Baseline[1]	0.096(0.0091)	-	1944	0.048 (0.0022)	-	648	-	-	-	-		A statistically significant reduction in ED visits of 0.021 per month (p = 0.035) was found for patients in the intervention group who received the intervention.	HIGH RISK OF BIAS
				12	0.070 (0.0050)*	-	1944	0.049 (0.0023)*	-	648	ATET: 0.021*	-	-	0.035*			
Reidt et al. 2016	Quasiexperimental	ED visits	Electronic Health Record	1	-	12.6	85	-	24.9	189	OR: 0.46	0.22	0.97	-		Patients in the intervention group had significantly fewer emergency department visits compared with the control group.	HIGH RISK OF BIAS
Westberg et al. 2014	Quasiexperimental	ED visits	Electronic Health Records	1	0.01 (0.09)	-	134	0.04 (0.21)	-	266	-	-	-	0.641		No statistically significant differences in ED visits were found between groups at 1,2 or 6 months.	MODERATE RISK OF BIAS
				2	0.08 (0.30)	-	134	0.11 (0.40)	-	262	-	-	-	0.521			
				6	0.44 (1.03)	-	133	0.41 (0.94)	-	258	-	-	-	0.641			

						INTERVENTION			CONTROL		Effect Size						
Authors/ Year	Design	Outcome	Tool	Time point (months)	Mean (SD)/ Median [IQR]	%	N	Mean (SD)/ Median [IQR]	%	N	DID/ OR	CI 95% lower limit	CI 95% upper limit	P value	Results	Interpretation	Risk of Bias
Jarab et al.	RCT	ED visits for	Electronic Medical	Baseline[3]	-	16.7	66	-	16.4	67	-	-	-	0.96		There were no significant differences between	HIGH RISK OF

2012	RCT	exacerbation	Record	6	-	15.2	66		17.9	67	-	-	-	0.79		groups in ED visits for acute exacerbation.	BIAS

1.12 months pre-intervention
ATET: Average Treatment Effect on the Treated
* Expressed as Monthly rates (predicted ED visits)

The eleven studies that evaluated the impact of interventions on emergency department (ED) visits had mixed results. Moreno et al. reported significant reductions in ED visits among the intervention group, using electronic health records for data collection. Reidt et al., also found a significant decrease in ED visits in the intervention group compared with the control group.

In contrast, Karapinar-Çarkıt et al., Campins et al., Del Cura González et al., McCarthy et al., Sáez de la Fuente et al., Syafhan et al., Yang et al., Matzke et al., and Westberg et al. failed to demonstrate statistically significant differences in the number of ED visits between the intervention and control groups. These studies used a variety of measurement tools, including electronic health records and self-reported questionnaires.

Indicates a significant improvement in the outcome measure for the intervention group compared to the control group.

Indicates a non-significant effect or no clear difference in the outcome measure between the intervention and control groups.

Indicates a negative effect, meaning a worsening of the outcome measure in the intervention group compared to the control group.

LENGTH OF STAY					INTERVENTION		CONTROL		Effect Size						
Author/Year	Design	Outcome	Tool	Time point (months)	Mean (SD)	N	Mean (SD)	N	MD/RR/IRR/β	CI 95% lower limit	CI 95% upper limit	P value	Results	Interpretation	Risk of Bias
Bolas et al. 2004	RCT	Length of stay	-	3	-	81	-	81	-	-	-	>0.05		There were no significant differences in length of stay between groups.	HIGH RISK OF BIAS
Briggs et al. 2015	RCT	Length of stay	-	4	6 (12)	525	6 (11)	496	MD: 0.09	-0.08	0.25	0.31		The mean difference in length of stay between groups was 0.09 days, indicating an slight increase in length of stay in the intervention group, which was not statistically significant	SOME CONCERNS
Lembeck et al. 2019	RCT	Length of stay [1]	National Health Service Register	1	545	270	440	267	β: 0.36	-0.34	01.06	-		The effect size (β = 0.36) suggests a positive trend toward longer stays in the intervention group at 1 month, although this finding was not statistically significant./ The effect size (β = -0.76) suggests a positive trend toward shorter stays in the intervention group at 6 months, although this finding was not statistically significant.	SOME CONCERNS
				6	1660	270	1830	267	β: -0.76	-2.70	1.18	-			
Lenander et al. 2014	RCT	Length of stay	Records of County Council	12	12 (-)	75	17.01 (-)	66	-	-	-	-		No statistically significant differences in length of stay between the intervention and control groups were found.	HIGH RISK OF BIAS
McCarthy et al. 2022	RCT	Length of stay [2]	Electronic Medical Record	6	2.43 (6.16)	188	03.07 (9.79)	171	IRR: 1.50	0.522	4.308	0.451		An incidence rate ratio of 1.50, indicating an increase in the length of stay in the intervention group compared to the control group; however, the result was not statistically significant.	HIGH RISK OF BIAS
			-	0	19.0 (12.2)	42	14.9 (12.9)	40	-	-	-	-			
				6	9.8 (8.9)	34	13.1 (11.5)	45	RR:1.1	0.5	2.3	0.850			

Author/Year	Design	Outcome	Tool	Time point (months)	INTERVENTION Mean (SD)	N	CONTROL Mean (SD)	N	Effect Size MD/RR/IRR/β	CI 95% lower limit	CI 95% upper limit	P value	Results	Interpretation	Risk of Bias
Muth et al. 2018	RCT	Length of stay [3]	-	9	28.0 (11.6)	28	9.7 (8.2)	25	RR: 0.4	0.1	2.8	0.336		The mean number of days spent in hospital had dropped by half after 6 months, but in both groups the event rate was too small to show significant differences.	LOW RISK OF BIAS
Syafhan et al. 2021	RCT	Length of stay	Electronic record system	6	4.4 (3.8)	169	4.0 (3.9)	161	-	-	-	0.733		No significant differences in length of stay between groups were found.	HIGH RISK OF BIAS
Yang et al. 2022	RCT	Length of stay [4]	Self-report questionnaire	3	3.54 (4.61)	67	2.49 (3.53)	69	β: 0.45	-1.69	2.60	0.678		The effect size (β = 0.45) suggests a positive trend toward longer stays in the intervention group,although this finding was not statistically significant.	SOME CONCERNS
Odeh et al. 2019	Quasiexperimental	Length of stay [5]	Hospital's computer records	3	8.3 (-)	211	6.7 (-)	211	-	-	-	<0.001		The intervention group had a significantly longer length of stay compared to the control group.	HIGH RISK OF BIAS

MD: Mean Differences
RR: Relative Risk
IRR: Incidence Rate Ratio
β: Group effect
1.Total number of days in the hospital
2.Number of inpatient days
3.Number of days spent in hospital
4.Days in the hospital
5.Length of stay on first readmission

Indicates a significant improvement in the outcome measure for the intervention group compared to the control group.

Indicates a non-significant effect or no clear difference in the outcome measure between the intervention and control groups.

Indicates a negative effect, meaning a worsening of the outcome measure in the intervention group compared to the control group.

Among the nine studies assessing length of stay as an outcome, most indicated no significant differences between intervention and control groups, although some reported trends suggesting longer stays in the intervention group.

Bolas et al. employed unspecified measures and found no significant differences in length of stay between the groups. Similarly, Lenander et al. analysed records from the County Council and also observed no significant differences in length of stay over 12 months.

Briggs et al., without specifying the measurement tool, reported a slight increase in length of stay in the intervention group, though this was not statistically significant. Lembeck et al., with data from the National Health Service Register, found no signifficant differences in length of stay between groups at 1 or 6 months, though trends toward longer stays in the intervention group were observed at 1 month .McCarthy et al. employed Electronic Medical Records and reported a trend toward increased length of stay in the intervention group, although this result was not statistically significant. Muth et al. found that the mean number of days spent in the hospital dropped after 6 months, but without significant differences.

Syafhan et al. used an electronic record system and reported no significant differences in length of stay between the groups. Yang et al. employed a self-reported questionnaire and reported an effect size suggesting a positive trend toward longer stays in the intervention group, but without statistical significance.

Notably, Odeh et al. used the hospital computer system and found that the intervention group had a significantly longer length of stay compared to the control group, indicating a clear difference with statistical significance.

NUMBER OF DRPs					INTERVENTION		CONTROL				
Authors/ Year	Design	Outcome	Tool	Time point (months)	Mean (SD)/ Mean [95%CI] / Median [IQR]	N	Mean (SD)/ Mean [95%CI]	N	Results	Interpretation	Risk of Bias
Ahmad et al. 2012	Cluster-RCT	DRPs	Medication analysis	Baseline	1.51 (-)	180	1.58 (-)	160		The mean number of DRPs identified with the medication analysis decreased from baseline to follow-up in the intervention group. In the control group, the number of DRPs increased from baseline to follow-up. The mean number of DRPs identified through the patient interview in the intervention group decreased from baseline to follow-up. In the control group, the mean number of DRPs increased from baseline to follow-up.	HIGH RISK OF BIAS
				12	1.37 (-)	167	1.62 (-)	152			
		DRPs	Patient Interview	Baseline	3.88 (-)	180	2.73 (-)	160			
				12	2.33 (-)	167	2.80 (-)	152			
Chrischilles et al. 2014	RCT	DRPs	Self-report	Baseline	1.4 (1.4)	802	1.5 (1.5)	273		No significant differences in the mean number of DRPs were observed between the intervention and control groups at any time point.	HIGH RISK OF BIAS
				6	1.4 (1.4)	802	1.6 (1.5)	273			
Lenander et al. 2014	RCT	DRPs	Self-report questionnaire	Baseline	1.73 [1.42,2.05]	75	1.37 [1.07,1.69]	66		Significant changes were seen in the before-and-after comparison in the intervention group, but not in the control group. A between-group analysis of the change in number of DRPs revealed no significant differences (p = 0.72).	HIGH RISK OF BIAS
				12	1.31 [1.02,1.59]	75	1.11 [0.84,1.37]	66			
Syafhan et al. 2021	RCT	DRPs	Electronic record system	Baseline	3.0 [2–4]	118	-	-		There was a significant decrease (p < 0.001) in median [IQR] numbers of MRPs per patient between baseline and 6 months.	HIGH RISK OF BIAS
				6	0.5 [0–1]	118	-	-			
Moczygemba et al. 2011	Quasiexperimental	DRPs	Electronic Medical Chart	Baseline	4.8 (2.7)	60	9.2 (2.9)	60		A reduction of 48% was showed in the intervention group while in the control group there was a reduction of 14%. The intervention group had significantly more MHRPs resolved (P = 0.0003).	HIGH RISK OF BIAS
				6	2.5 (2.0)	60	7.9 (3.0)	60			

Among the five studies that assessed drug-related problems (DRPs), Ahmad et al. employed medication analysis and found that the mean number of DRPs identified decreased from baseline to follow-up in the intervention group, while the control group experienced an increase. Additionally, through patient interviews, the intervention group showed a reduction in DRPs, contrasting with an increase in the control group at 12 months. Chrischilles et al., using self-reported questionnaires, found no significant differences in the mean number of DRPs between the intervention and control groups at any time point, indicating that the intervention did not have a measurable impact on DRPs. Similarly, Lenander et al. also employed a self-reported questionnaire and observed significant changes in the intervention group in the before-and-after comparison. However, the between-group analysis revealed no significant differences.
Syafhan et al. used an electronic record system to assess medication-related problems , finding a significant decrease in the median number of DRPs per patient between baseline and six months; and Moczygemba et al., employing an electronic medical chart, found that the intervention group had significantly more Medication Health Related Problems resolved.

Authors/ Year	Design	Outcome	Tool	Time point (months)	%	n	N	%	N	Results	Interpretation
**PROPORTION OF PATIENTS WITH DRPS**											
Ahmad et al. 2012	Cluster-RCT	Increased number of DRPs	Medication analysis	12	8.4	-	167	20.2	152		The intervention group had a significantly lower proportion of patients with an increased number of DRPs (8.4%) compared to the control group (20.2%).
		Unchanged number of DRPs	Medication analysis	12	69.0	-	167	61.7	152		
		Decreased number of DRPs	Medication analysis	12	22.6	-	167	18.1	152		
**PROPORTION OF SOLVED DRPs**											
Geurts et al. 2016	RCT	Solved DRPs	-	12	47.2	394	-	-	-		Only the proportion of solved DRPs in the intervention group was reported, reaching 42%.
Köberlein-Neu et al. 2018	Cluster-RCT Stepped-wedge design	Selection of substance	Patient file (prescribed drugs)	15	26.0	791	-	-	-		The intervention aimed at solving various categories of DRPs with notable rates of resolution in issues such as medication not available/ incorrect dispense.
		Galenic formulation			0.0	6	-	-	-		
		Seleceted dosage			30.1	312	-	-	-		
		Duration of treatment			32.0	25	-	-	-		
		Problems with use			27.2	320	-	-	-		
		Medication not available, incorrect dispense			60.0	10	-	-	-		
		Forgets to take medication, medication kept in inappropriate place			28.6	21	-	-	-		
		Other reason			33.0	103	-	-	-		

Ahmad et al. employed medication analysis to assess the proportion of patients with increased, unchanged, and decreased numbers of DRPs at 12 months. They found a significant difference, with only 8.4% of the intervention group experiencing an increased number of DRPs, compared to 20.2% in the control group.

Among studies assessing the resolution of DRPs, Köberlein-Neu et al., using data from patient files, reported substantial success in addressing issues such as medication availability and dosage selection. Notably, 60% of patients experiencing medication availability issues had their DRPs resolved.

Additionally, Geurts et al. reported that 47.2% of DRPs were resolved in their intervention group, although the specific tool used was not mentioned.

ADRs/ADES/ADWEs	INTERVENTION	CONTROL	Effect Size	

Authors/ Year	Design	Outcome	Tool	Time point (months)	Mean [95%CI]/ Median (IQR)	%	n	N	Mean [95%CI]/ Median (IQR)	%	N	OR/ Adjusted Diff.	CI 95% lower limit	CI 95% upper limit	P value	Results	Interpretation	Risk of Bias
Del Cura-González et al. 2022	Cluster-RCT	ADRs[1]	Reported by the Family Physicians	6	1(1-1)	-	-	287	1(1-1)	-	285	Adjusted Diff. : 0.49	-0.30	1.28	0.223		There was also no evidence that the intervention reduced the number of adverse drug events	SOME CONCERNS
				12	1 (1-2)	-	-	269	1 (1-1)	-	277	Adjusted Diff. : 0.49	-0.12	1.11	0.11			
Lenander et al. 2014	RCT	ADEs	Self-report	Baseline	0.64[0.46, 0.82]	-	-	75	0.53 [0.33, 0.73]	-	66	-	-	-	-		No significant changes were noted in the number of adverse drug events pre- and post-intervention.	HIGH RISK OF BIAS
				12	0.52 [0.36, 0.68]	-	-	75	0.50 [0.34, 0.66]	-	66	-	-	-	-			
Leendertse et al. 2013	Quasiexperimental	ADEs	Self-report	12	-	28.6	-	364	-	23.5	310	OR: 1.02	0.94	01.08	-		The number patients with one or more adverse events in the intervention group seemed slightly higher than in the control group but not significant.	HIGH RISK OF BIAS
Herrinton et al. 2023	RCT	ADWEs	Internal Data Safety Monitoring Committee[2]	6	-	-	-	1237	-	-	1233	-	-	-	-		No statistically significant Difference-in-differences in ADWEs.	LOW RISK OF BIAS
McCarthy et al. 2022	RCT	ADWEs	Self-report/ Reported by the GP	6	-	1.81	826	-	-	-	-	-	-	-	-		1 serious event (depression)<br>10 events likely related to drug withdrawal; mild reactions resolved with reinstitution of the drug.	SOME CONCERNS
Taylor et al. 2003	RCT	Medication misadventure[3]	Self-report/ Reported by nurses, physicians/ Chart reviews	12	-	2.8	33	3.0	36	-	-	-	-	-	0.731		There were no significant differences between groups aT 12 months in medication misadventures.	HIGH RISK OF BIAS

n = number of medications discontinued
1. ADRs: Adverse drug reactions
2. Included a biostatistician, cardiologist, gastroenterologist, and pulmonologist who met every 6 months to compare the rates of ADWEs in the intervention and usual care groups
3. Patients with at least one medication misadventure

Among studies evaluating ADRs and ADEs, Del Cura-González et al. found no significant reduction in ADRs at 6 and 12 months. Similarly, Lenander et al. reported no significant changes in ADEs between baseline and 12 months. Leendertse et al. indicated a slight increase in ADEs in the intervention group, but the difference was not significant. Taylor et al. also found no significant differences between the intervention and control groups in medication misadventures at twelve months.Lastly, McCarthy et al. noted 1 serious ADWE (depression) and ten mild reactions related to drug withdrawal among 826 medications discontinued.

*Each study defines and measures Drug-Related Problems (DRPs), Adverse Drug Events (ADEs), Adverse Drug Reactions (ADRs), and Adverse Drug Withdrawal Events (ADWEs) differently, reflecting the variability in classification and assessment methods across studies. This variation, must be considered when interpreting and comparing the results.	
**AUTHORS/ YEAR**	**CLASSIFICATION/ ASSESSMENT**
Ahmad et al. 2012	Drug-related problems were categorized using the Pharmaceutical Care Network Europe DRP-score form.5 A
Chrischilles et al. 2014	The mean number of medication management problems was calculated from endorsed items based on the Use Self-Evaluation (MUSE) tool (Chang et el., 2011)
Del Cura-González et al. 2022	Medication safety was measured as the incidence (number of events per patient year) of adverse drug reactions reported by the FP and potentially hazardous interaction using the taxonomy proposed by Otero-López et al., 2003
Geurts et al. 2016	Drug Related Problems (DRPs) and Pharmaceutical care Issues (PCIS)
12	Adverse Drug Withdrawal Events (ADWEs) were defined as adverse clinical outcomes potentially related to loss of disease control following medication withdrawal, including emergency department visits and hospitalizations for respiratory, cardiovascular, gastrointestinal, or metabolic disorders
Köberlein-Neu et al. 2018	Classification according to Pharmaceutical Care Network Europe (PCNE) version 6.2
Lenander et al. 2014	DRPs were classified based on Beers' criteria and the structure proposed by Strand et al. (1990)
McCarthy et al. 2022	Adverse Drug Withdrawal Events (ADWEs) were defined as either recurrence of the condition for which the drug was prescribed or a physiological withdrawal reaction. Events were identified through GP reports and patient self-reports. Causality was assessed using an adapted version of the Naranjo Adverse Drug Reaction (ADR) Probability Scale, tailored for withdrawal events within the SPPiRE deprescribing protocol

Syafhan et al. 2021	Medication Related Problems (MRPs) according to the classification devised by AbuRuz et al. (2006)
Moczygemba et al. 2011	Medication and Health Related Problems (MHRPs): The problems included: (1) therapeutic duplication; (2) drug efficacy; (3) drug safety; (4) cost/formulary interchange; (5) precaution/contraindication/interaction related to age, disease, or drug; and (6) preventive care need
Taylor et al. 2003	Medication errors, ADES, and ADRS are collectively defined as medication misadventures, in which an iatrogenic incident occurs that may be attributable to "error, immunologic response, or idiosyncratic response and is always unexpected or undesirable to the patient.

PRIMARY CARE CONTACTS				INTERVENTION			CONTROL		Effect size						
**Outcome**	**Tool**	**Timepoint**	**Mean (SD)/ Median [IQR]**	**n**	**N**	**Mean (SD)/ Median [IQR]**	**n**	**N**	**OR/ Adjusted DIfference / IRR/ Diff. Proportion/ β**	**CI 95% lower limit**	**CI 95% upper limit**	**P value**	**Results**	**Interpretation**	**Risk of Bias**
Contacts with GP²	Self-report	0	4.79 (8.39)	-	1290	4.27(6.17)	-	1164	-	-	-	-		In the pooled data, there were no significant differences between the control and intervention patients at any assessment point with regard to contacts with GPs	HIGH RISK OF BIAS
		6	3.97 (5.69)	-	1024	3.57 (4.58)	-	953	-	-	-	>0.05			
		12	4.00 (7.00)	-	863	3.53 (5.54)	-	764	-	-	-	>0.05			
		18	4.25 (7.98)	-	704	3.24 (4.03)	-	636	-	-	-	>0.05			
Primary care visits	Electronic primary care clinical histories	3	7.32 (5.48)	-	252	6.02 (4.69)	-	251	-	-	-	0.001		The intervention group had significantly higher primary care visits compared to the control group at 3 and 6 months, but no significant difference at 12 months.	SOME CONCERNS
		6	12.92 (9.59)	-	252	11.4 (8.01)	-	251	-	-	-	0.048			
		12	24.0 (16.8)	-	252	23.0 (14.1)	-	251	-	-	-	0.670			
Gp Visits	-	6	3 [2-6]	-	287	4 [2-6]	-	287	Adjusted Difference: 0.073	-0.109	0.256	0.432		The adjusted difference values suggest a very small, non-significant difference in GP visits and primary care nurse consultations between the intervention and control groups at 6 and 12 months, indicating a trend toward more visits in the intervention group.	HIGH RISK OF BIAS
		12	7 [4-11]	-	272	7 [4-10]	-	280	Adjusted Difference : 0.07	-0.11	0.25	0.44			
Primary Care Nurse Consultations	-	6	3 [2-6]	-	287	2 [1-5]	-	287	Adjusted Difference: 0.168	-0.081	0.416	0.186			
		12	4 [2-8]	-	272	4 [2-7]	-	280	Adjusted Difference : 0.10	-0.15	0.35	0.43			
Primary care visits	Records of County Council	12	-	-	75	-	-	66	-	-	-	-		"No significant differences were observed between the intervention and control groups regarding primary care utilization during the 12-month follow-up."	HIGH RISK OF BIAS
Gp visits	Electronic Medical Record	6	4.42 (3.51)	-	188	3.83 (3.26)	-	171	IRR:1.06	0.840	1.348	0.608		An IRR of 1.06 suggests a slight increase in the rate of GP visits in the intervention group compared to the control group; however, the intervention group had a higher rate of GP telephone consultations (IRR: 1.75) with a p-value of 0.052, indicating a trend towards significance.	HIGH RISK OF BIAS
GP Telephone consultations	Electronic Medical Record	6	1.55 (2.12)	-	188	1.41 (2.21)	-	171	IRR: 1.75	0.995	3.081	0.052			
GP attendances	Patient administration system	3	-	101	130	-	108	144	Diff. prop: 2.7	-2.4	12.7%	-		There was a 2.7% higher proportion of GP attendances in the intervention group compared to the control group at 3 months; however, this difference was not statistically significant. At 6 months, a very smallnon-significant difference was found between groups.	HIGH RISK OF BIAS
		6	-	76	102	-	82	116	Diff. prop: 0.3	11.6	12.3%	-			

				INTERVENTION			CONTROL		Effect size						
**Outcome**	**Tool**	**Timepoint**	**Mean (SD)/ Median [IQR]**	**n**	**N**	**Mean (SD)/ Median [IQR]**	**n**	**N**	**OR/ Adjusted DIfference / IRR/ Diff. Proportion/ β**	**CI 95% lower limit**	**CI 95% upper limit**	**P value**	**Results**	**Interpretation**	**Risk of Bias**
Primary care visits	Patient Interview/ Medical History	1-1.6	-	-	26	-	-	24	OR: 1.3	0.4	3.8	0.63		An odds ratio of 1.3 indicates a greater number of primary care visits in the intervention group, although this finding does not achieve statistical significance.	HIGH RISK OF BIAS
Face and Telephone Consultations	Electronic record system at Gp practice	6	5.7 (4.3)	-	169	6.5 (5.3)	-	161	-	-	-	0.284		No significant differences were observed in total face-to-face and telephone consultations between the intervention and control groups.	HIGH RISK OF BIAS
GP visits	Self-report	6	3.8 (3.3)	-	56	2.9 (4.3)	-	67	-	-	-	>0.05		No significant differences were found in GP visits or home visits between the intervention and control groups.	HIGH RISK OF BIAS
GP Home visits	Self-report	6	1.4 (1.9)	-	56	1.2 (2.6)	-	67	-	-	-	>0.05			
Community Health Centre visits	Self-report questionnaire	3	0.98 (1.0)	-	67	1.22 (0.93)	-	69	β: 0.21	-0.11	0.54	0.193		No significant differences were found in GP visits or home visits between the intervention and control groups.	SOME CONCERNS

n: number of contatcs with Primary care
OR: Odds Ratio
IRR: Incidence Rate Ratio
β: Group effect
Diff. proportion: Difference in proportion between groups

Indicates a significant improvement in the outcome measure for the intervention group compared to the control group.

Indicates a non-significant effect or no clear difference in the outcome measure between the intervention and control groups.

Indicates a negative effect, meaning a worsening of the outcome measure in the intervention group compared to the control group.

Among the 10 studies that assessed contacts with primary care as an outcome,most suggested a small increase in visits within the intervention group; however, only one study achieved statistical significance. Bernsten et al.employed self-reported measures and found no significant differences in contacts with general practitioners between the intervention and control groups at any time point. In contrast, Campins et al. used Electronic Primary Care Clinical Histories and reported significantly higher primary care visits in the intervention group at 3 and 6 months, but no significant difference at 12 months. Lenander et al. analysed records from the County Council and also observed no significant differences in primary care utilization over 12 months.

Del Cura-González et al. did not specify the tool used for assessment but found a very small, non-significant adjusted difference in GP visits and primary care nurse consultations between the intervention and control groups at both 6 and 12 months, suggesting a trend toward more visits in the intervention group.

Nazareth et al. reported a difference in proportions of 2.7% favouring the intervention group at 3 months, though this was not statistically significant; a small difference remained at 6 months. McCarthy et al. used Electronic Medical Records and reported a slight increase in GP visits in the intervention group, although the differences were not statistically significant; the intervention group exhibited a higher rate of GP telephone consultations, indicating a trend toward significance.

Sáez de la Fuente et al. through patient interviews and found a greater number of primary care visits in the intervention group, but this without statistical significance. Syafhan et al. used data from electronic record system and reported no significant differences in total face-to-face and telephone consultations between groups. Van der Heijden et al. used self-reported questionnaires and found no significant differences in GP visits or home visits. Lastly, Yang et al. employed a self-reported questionnaire and similarly reported no significant differences in community health centre visits between groups.

OUTPATIENT VISITS				INTERVENTION		CONTROL		Effect Size						
**Design**	**Outcome**	**Tool**	**Timepoint (months)**	**Mean (SD)**	**N**	**Mean (SD)**	**N**	**DID/ IRR/ β**	**CI 95% lower limit**	**CI 95% upper limit**	**p value**	**Results**	**Interpretation**	**Risk of Bias**
RCT	Outpatient visits	Electronic Health Records	6	-	1237	-	1231	DID: 0.4	-0.3	1.1	0.26		The intervention group had an average of 0.4 more outpatient visits than the control group. However, this difference was not statistically significant.	LOW RISK OF BIAS
RCT	Outpatient visits	Electronic Medical Record (Reported by the GP)	6	2.28 (5.54)	188	2.39 (2.62)	171	IRR: 0.97	0.712	1.333	0.869		The IRR of 0.97 suggests that the intervention group had a slightly lower rate of outpatient visits compared to the control group; however, this result wasalso not statistically significant.	HIGH RISK OF BIAS
RCT	Outpatient visits	Electronic record system at Gp practice	6	2.5 (3.1)	169	2.8 (2.9)	161	-	-	-	0.841		There were no differences between the two groups at 6 months.	HIGH RISK OF BIAS
RCT	Outpatient visits	Self-report questionnaire	3	0.61 (0.56)	67	0.67 (0.66)	69	β: 0.09	-0.23	0.41	0.587		The group effect indicates a slight increase in outpatient visits in the intervention group; however, this increase was not statistically significant.	SOME CONCERNS

				INTERVENTION		CONTROL		Effect Size						
**Design**	**Outcome**	**Tool**	**Timepoint (months)**	**n**	**N**	**n**	**N**	**Diff. Proportion**	**CI 95% lower limit**	**CI 95% upper limit**	**p value**	**Results**	**Interpretation**	**Risk of Bias**
RCT	Outpatient visits	Patient administration system	3	75	164	84	176	2	-12.6	8.6%	-		There were no differences regarding outpatient visits between the two groups after 3 or 6 months.	HIGH RISK OF BIAS
			6	39	137	40	151	2	-8.3	12.3%				

IRR: Incidence Rate Ratio
DID: Difference-in-Differences
HR: Hazard Ratio
β: Group effect
n: number of patients with outpatient visits
Diff. proportion: Difference in proportion between groups

Indicates a significant improvement in the outcome measure for the intervention group compared to the control group.

Indicates a non-significant effect or no clear difference in the outcome measure between the intervention and control groups.

Indicates a negative effect, meaning a worsening of the outcome measure in the intervention group compared to the control group.

The studies examining the impact of interventions on outpatient visits produced mixed results. Herrinton et al. reported that the intervention group had an average of 0.4 more outpatient visits than the control group; however, this difference was not statistically significant. McCarthy et al. found an incident rate ratio (IRR) of 0.97, suggesting a slightly lower rate of outpatient visits in the intervention group, which also did not reach statistical significance. Syafhan et al. found no differences between the groups after six months. All of these studies used Electronic Health Records tocollect data.
In contrast, Yang et al. employed self-reported questionnaires and noted a slight increase in outpatient visits for the intervention group, but this finding too was not statistically significant.

In the analysis of patients with outpatient visits, Nazareth et al. found no differences between the intervention and control groups at both 3 and 6 months, assessed through a patient administration system.

SPECIALIST PHYSICIAN VISITS					INTERVENTION			CONTROL			Effect Size						
**Authors/ Year**	**Design**	**Outcome**	**Tool**	**Timepoint**	**Mean (SD)**		**N**	**Mean (SD)**		**N**	**OR**	**CI 95% lower limit**	**CI 95% upper limit**	**P value**	**Results**	**Interpretation**	**Risk of Bias**
Campins et al. 2017	RCT	Specialist Physician visits	Electronic primary care clinical histories	3	1.50 (2.41)		252	1.61 (2.12)		251	-	-	-	0.986		There were no significant differences in the number of specialist physician visits between the intervention and control groups at 3 months, 6 months and 12 months.	SOME CONCERNS
				6	2.89 (3.46)		252	2.81 (3.61)		251	-	-	-	0.253			
				12	6.9 (7.3)		252	6.8 (7.6)		251	-	-	-	0.302			
Sáez de la Fuente et al. 2011	RCT	Specialist Physician visits	Patient Interview/ Medical History	1-1.6	-		25.1	-		23.1	2.7	0.9	8.0	0.08		The OR of 2.7 indicates a trend towards increased visits in the intervention group; however, the difference was not statistically significant.	HIGH RISK OF BIAS
Van der Heijden et al. 2019	Cluster-RCT	Specialist Physician visits	Self-report	6	9.9 (9.6)		56	9.2 (9.6)		67	-	-	-	>0.05		"During the first 6 months after hospital discharge patients in the intervention group more frequently visited their medical specialist, although these differences were not statistically significant"	HIGH RISK OF BIAS

OR: Odds Ratio

Indicates a significant improvement in the outcome measure for the intervention group compared to the control group.

Indicates a non-significant effect or no clear difference in the outcome measure between the intervention and control groups.

Indicates a negative effect, meaning a worsening of the outcome measure in the intervention group compared to the control group.

Studies assessing the impact of interventions on specialist physician visits showed inconclusive results. Campins et al. used Electronic Primary Care Clinical Histories and reported no significant differences between the intervention and control groups at 3, 6, or 12 months. Similarly, Sáez de la Fuente et al., employed patient interviews and medical history, and observed an OR of 2.7, suggesting a trend toward more visits in the intervention group; however, this result was not statistically significant. Van der Heijden et al. used self-reported questionnaire and found that patients in the intervention group visited their specialists more frequently in the first six months post-discharge, but again, these differences were not statistically significant.
