# Supplementary material for: Interventions to enhance in-home taking medication among older adults with multimorbidity/polypharmacy: a systematic review and meta-analysis
Source: Front Public Health. 2026 Jan 28;13:1701622. doi: 10.3389/fpubh.2025.1701622 (PMC12891206; doi:10.3389/fpubh.2025.1701622)
Supplement: Supplementary file 1 [file Data_Sheet_1.zip › Supplementary Table 8.Secondary Outcomes.pdf]

QUALITY OF LIFE					INTERVENTION		CONTROL		Effect Size						
**Authors/Year**	**Design**	**Outcome**	**Tool**	**Timepoint**	**Mean (SD)**	**N**	**Mean (SD)**	**N**	**Adjusted Difference /Diff. in change/ β**	**CI 95% lower limit**	**CI 95% upper limit**	**P value**	**Results**	**Interpretation**	**Risk of Bias**
Campins et al. 2017	RCT	Quality of life	EuroQol-5D	6	-	251	-	251	-	-	-	-		The intervention made no difference in quality of life which, at 6 months, remained largely stable in both groups, with a change from the baseline score (on a scale of 0–100) of −2.09 points in the intervention group and of 0.67 points in the control group (P = 0.324).	SOME CONCERNS
Del-Cura González et al. 2022	Cluster-RCT	Quality of life	EuroQol-5D	6	0.777 (0.221)	286	0.804 (0.170)	287	Adjusted Difference: -0.016	-0.041	0.010	0.222		No significant differences in Quality of life between groups were found.	HIGH RISK OF BIAS
				12	0.763 (0.213)	280	0.780 (0.182)	272	Adjusted Difference: -0.006	-0.034	0.022	0.68			
Holland et al. 2005	RCT	Quality of life	EuroQol-5D	3	0.47 (0.32)	320	0.48 (0.32)	325	-	-	-	-		The quality of life scores for both groups remained similar over time, with no significant differences observed at 3 months or 6 months.	HIGH RISK OF BIAS
				6	0.46 (0.33)	311	0.50 (0.31)	288	Diff. in change: 0.006	-0.048	0.059	0.84			
Jerant et al. 2009	RCT	Quality of life	EuroQol-5D	0	0.73(0.18)	139	0.75(0.16)	138	-	-	-	-		EuroQol-5D scores remained stable across both groups at 6 and 12 months, with no significant differences.	HIGH RISK OF BIAS
				6	0.81 (0.18)	139	0.80 (0.20)	138	-	-	-	-			
				12	0.77 (0.20)	139	0.81 (0.17)	138	-	-	-	-			
Lenaghan et al. 2007	RCT	Quality of life	EuroQol-5D (Utility score)	6	-	56	-	49	Diff. in change: 0.09	-0.19	0.02	0.10		In both groups, the EQ-5d utility score decreased over 6 months follow-up. There was a small difference in the change in utility scores over 6 months in favour of the control group, but this was not statistically significant.	SOME CONCERNS
McCarthy et al. 2022	RCT	Quality of life	EuroQol-5D	6	0.517 (0.382)	118	0.456 (0.357)	111	MD: 0.011	-0.059	0.081	0.753		The mean difference in quality of life scores between the intervention and control groups was minimal and not significant.	HIGH RISK OF BIAS
Muth et al. 2018	RCT	Quality of life	EuroQol-5D	0	0.739 (0.244)	241	0.749 (0.230)	240	-	-	-	-		Non-significant changes were found in quality of life. In the intervention group, patients' self-reported quality of life improved minimally after 6 and 9 months, whereas it continued to decline in the control group .	LOW RISK OF BIAS
				6	0.739 (0.238)	229	0.732 (0.248)	225	MD: 1.4	-2.5	5.3	0.471			
				9	0.748 (0.234)	222	0.728 (0.251)	214	MD: 2.3	-1.6	6.2	0.247			
Syafhan et al. 2021	RCT	Quality of life	EuroQol-5D	6	-	119	-	130	-	-	-	-		Control group patients showed a slight decline from baseline in the EQ-5D-5L utility scores at the end of study (from a median of 0.616 to 0.596), while there was no change in this score in the intervention group patients (medians of 0.647 to 0.648).	HIGH RISK OF BIAS

Yang et al. 2022	RCT	Quality of life	EuroQol-5D	3	0.86 (0.14)	67	0.84 (0.15)	69	β: 0.00	-0.04	0.05	0.912		No statistically significant interaction effect between group and time was found on the EQ-5D-5L index value, self-rated health, or utilisation of health care services at either T1 or T2.	SOME CONCERNS
Leendertse et al. 2013	Quasiexperimental	Quality of life	EuroQol-5D	11.1	-	364	-	310	-	-	-	-		The difference in quality of life between the end of the inclusion period and the start of the inclusion period, measured with the EuroQol EQ5D questionnaire, was 0.02 in the intervention group and 0.00 in the control group [0.16 (95% CI: 0.01–0.42)]	HIGH RISK OF BIAS

Diff. in change: Difference in change
β: Group effect

Among the ten studies assessing quality of life using the EuroQol-5D, none found significant differences between the intervention and control groups. Del-Cura González et al. reported stable quality of life scores at both 6 and 12 months, and Yang et al. found no significant effects on the EQ-5D index.

Lenaghan et al. observed a small, non-significant change favouring the control group over 6 months. Similarly, Syafhan et al. noted a slight decline in the control group's scores, with no change in the intervention groupy. Muth et al. reported minimal improvements in the intervention group after 6 and 9 months, while a decline was noted in the control group.

Five studies suggested positive trends in quality of life. Holland et al. and Jerant et al. found consistent scores across groups, with no significant differences. McCarthy et al. reported minimal, non-significant mean differences, and Campins et al. indicated no significant change in quality of life at 6 months. Lastly, Leendertse et al. identified a small, non-significant improvement favouring the intervention group.

Indicates a significant improvement in the outcome measure for the intervention group compared to the control group.

Indicates a non-significant effect or no clear difference in the outcome measure between the intervention and control groups.

Indicates a negative effect, meaning a worsening of the outcome measure in the intervention group compared to the control group.

DEATHS					INTERVENTION		CONTROL		Effect Size						
**Authors/ Year**	**Design**	**Outcome**	**Tool**	**Timepoint**	**%**	**N**	**%**	**N**	**HR/OR**	**CI 95% lower limit**	**CI 95% upper limit**	**P value**	**Results**	**Interpretation**	**Risk of Bias**
Campins et al. 2017	RCT	Deaths	Electronic primary care clinical histories	3	0.8	252	0.4	251	-	-	-	1.000		No significant effect of the intervention on mortality at 3, 6, or 9 months, with mortality rates being low and stable across both groups	SOME CONCERNS
				6	2.0	252	0.4	251	-	-	-	0.216			
				9	2.8	252	2.4	251	-	-	-	0.784			
Holland et al. 2005	RCT	Deaths	Office for National Statistics	6	-	415	-	414	HR: 0.75	0.52	1.10	0.14		Results were not statistically significant but favoured the intervention group, with a hazard ratio of 0.75.	HIGH RISK OF BIAS
Kouladjian O'Donnell et al. 2021	Cluster-RCT	Deaths	-	3	2.0	63	5.0	96	OR: 0.20	0.007	5.20	-		There were no statistically significant differences between the comparison and intervention groups for mortality, with an odds ratio of 0.20, suggesting a trend favoring the intervention group.	HIGH RISK OF BIAS
Lembeck et al. 2019	RCT	Deaths	Civil Registration System	1	9.0	270	6.0	267	-	-	-	0.26		Slightly different mortality rates at 1 and 6 months were found in both groups, but these differences were not statistically significant.	SOME CONCERNS
				6	23.0	270	22.0	267	-	-	-	0.66			
Nazareth et al. 2001	RCT	Deaths	Patient administration system	3	6.1	164	2.8	176	-	-	-	-		Higher mortality rates were observed in the intervention group at 3 and 6 months, but these differences were not statistically significant.	HIGH RISK OF BIAS
				6	16.1	137	12.6	151	-	-	-	-			
Olesen et al. 2014	RCT	Deaths	Electronic Hospital record	24	7.5	253	5.0	264	OR: 1.41	0.71	2.82	-		An OR of 1.41 was reported, indicating a slight trend toward higher mortality in the intervention group; however, this result was not statistically significant.	HIGH RISK OF BIAS
Sáez de la Fuente et al. 2011	RCT	Deaths	Medical History	1.6	5.2	29	1.7	30	OR: 2.2	0.19	26.1	0.51		An OR of 2.2 was observed, indicating higher mortality in the intervention group; however, the wide confidence interval and lack of statistical significance make this finding inconclusive.	HIGH RISK OF BIAS
Hugtenburg et al. 2009	Quasiexperimental	Deaths	Patient Administration Information System	9	22.0	336	22.0	379	-	-	-	-		"At the end of the 9-month study period, there was no difference between the mortality of patients of intervention pharmacies and patients of control pharmacies (22% in each group)"	HIGH RISK OF BIAS
Karapinar-Çarkıt et al. 2019	Quasiexperimental	Deaths	Hospital information systems	6	6.6	365	7.6	341	-	-	-	> 0.05		Mortality did not differ between groups.	HIGH RISK OF BIAS
Leendertse et al. 2013	Quasiexperimental	Deaths	Medical History	12	3.0	364	2.6	310	HR: 0.78	0.13	1.94	-		A HR of 0.78 was reported, suggesting a slight trend favoring the intervention group in survival rates at 12 months, though this difference was not statistically significant.	HIGH RISK OF BIAS

Westberg et al. 2014	Quasiexperimental	Deaths	Electronic Health Records	1	0.07	134	1.5	270	OR: 0.524	0.057	4.829	-		The reported OR indicated lower mortality at different timepoints in the intervention group, although none of these findings reached statistical significance.	MODERATE RISK OF BIAS
				3	0.7	135	3.3	270	OR: 0.266	0.028	1.821	-			
				6	3.0	133	5.6	270	OR: 0.587	0.188	1.826	-			

HR: Hazard Ratio
OR: Odds Ratio
Diff. proportion: Difference in proportion between groups

The eleven studies assessing intervention effects on mortality generally showed no statistically significant differences between intervention and control groups. Campins et al. used electronic primary care clinical histories and reported stable mortality rates at multiple timepoints. Nazareth et al., using a patient administration system, similarly observed no significant changes. Holland et al., with data from the Office for National Statistics, and Kouladjian O'Donnell et al., who did not specify the measurement tool, noted slight trends favoring the intervention group, but these findings were not significant.
Lembeck et al. with data from a civil registration system, found similar mortality rates in both groups at 1 and 6 months, with no statistically significant differences. Olesen et al., using electronic hospital records, and Sáez de la Fuente et al., via medical history records, observed higher mortality in the intervention group; however, these results were inconclusive due to lack of statistical significance and wide confidence intervals.
Quasi-experimental studies conducted by Hugtenburg et al. using patient data extracted from the patient administration system, Leendertse et al. using medical history records, Karapinar-Çarkıt et al. using hospital information systems, and Westberg et al. employing electronic health records revealed slight trends that favored the intervention group, showing lower odds or hazard ratios for mortality. However, these findings did not reach statistical significance.

Indicates a significant improvement in the outcome measure for the intervention group compared to the control group.

Indicates a non-significant effect or no clear difference in the outcome measure between the intervention and control groups.

Indicates a negative effect, meaning a worsening of the outcome measure in the intervention group compared to the control group.

COSTS					INTERVENTION			CONTROL			P value	Cost-effectiveness	Results	Notes	Risk of Bias
Author/Year	Design	Outcome	Tool	Time point (months)	n	Mean (SD)/ Mean [SE]	N	n	Mean (SD)/ Mean [SE]	N					
Bernsten et al. 2001	RCT	Total costs[1]	$	0-6	-	1,732.30 (-)	1024	-	1,889.62	953	-	-		Between-group analysis indicated that there were no significant differences between the total cost for control and intervention patients in any country (p > 0.05).	HIGH RISK OF BIAS
				6-12		1,723.65 (-)	863	-	2,088.32	764					
				12-18		1,809.63 (-)	704	-	1,893.32	636					
Bernsten et al. 2001	RCT	Cost-saving	$	0-6	-	-157.32 (-)	-	-	-		-	-		In most of the participating countries, cost savings were observed in intervention patients compared with control patients. Many of these cost savings were as a direct result of reduced drug costs or reduced costs associated with hospitalisations.	HIGH RISK OF BIAS
				6-12		-364.67 (-)	-	-	-						
				12-18		-83.70 (-)	-	-	-						
Jerant et al. 2009	RCT	Total Healthcare Costs	$	12	-	12,422 (14, 241)	139		11,493 (10,972)	138	-	-		No significant differences were found in total health care costs between groups.	HIGH RISK OF BIAS
Lenander et al. 2014	RCT	Cost of Intervention[2]	$	12	-	106 (-)		-	-	-	-	The study did not demonstrate savings in terms of reduced hospital admissions or decreased primary healthcare use, as it was primarily designed to detect a 25% reduction in drug-related problems (DRPs), not healthcare utilization.		The pharmacist had booked 30 minutes for each consultation, but including time for preparation and follow-up, each patient required approximately two hours. The cost of implementing this intervention in everyday practice was estimated at €79 ($106) per patient, based on the estimated total cost of one clinically trained, experienced pharmacist	HIGH RISK OF BIAS
Syafhan et al. 2021	RCT	Total healthcare resource utilisation cost [3]	$	Baseline	228,854.60	1,354.21 (1,880.71)	169	246,054.00	1,528.28 (2,105.09)	161	0.032[7] 0.276[8]	"Although the incremental costs and effects density straddled all four quadrants of the cost–effectiveness plane, the majority of the points lay in the dominant (south-east) quadrant, indicating improved outcomes linked with reduced cost."		The total cost of healthcare resource utilisation in intervention group patients, including intervention costs, showed a significant (p = 0.032) decrease when 6 months of pre-intervention data were compared with 6 months of post-intervention data.	HIGH RISK OF BIAS
				6	188,739.20	1,116.83 (1,606.76)	169	224,399.50	1,393.73 (2,126.50)	161					
Van der Heijden et al. 2019	Cluster-RCT	Total healthcare resource utilisation cost [4]	$	12	-	6,104 [1,157.20]	106	-	4,253.72 [489.84]	110	-	"In the cost-effectiveness analysis, most cost-effect pairs were located in the northeast quadrant, indicating that the Comprehensive Medication Review (CMR) at discharge was associated with higher costs while providing only a marginal increase in effectiveness compared to usual care in terms of reducing Drug-Related Problems"		Costs of resource use in the intervention group were higher than in the control group, but not statistically significant.	HIGH RISK OF BIAS
Yang et al. 2022	RCT	Medical costs	$	Baseline	-	246.30 (186.72)	67	-	251.63 (218.51)	69	0.922	-		No significant difference in medical costs was observed between intervention and control groups at baseline or three months.	SOME CONCERNS
				3	-	206.87 (130.70)	67	-	207.73 (61.68)	69	0.866	-			

Matzke et al. 2018	Quasiexperimental	Cost reduction [5] (ED visits/ Hospitalisations)	$	12	5,156,675	2,619 (-)	1,969	475,071	241 (-)	1,969	-	-		A considerable cost reduction in ED visits and hospitalizations was observed. The total cost of the intervention was estimated at $478 per patient, suggesting a favorable impact on emergency-related costs.	HIGH RISK OF BIAS
		Total cost of intervention	$	12	929,726	478 (-)	1,969	-	-	1,969	-	-			
Moczygemba et al. 2011	Quasiexperimental	Total costs of drug coverage [6]	$	Baseline	-	2,289 (887)	60	-	2,311 (1,148)	60	-	-		Although change in total Part D drug costs between the intervention and control groups was not significant in this study, a decrease in intervention group Part D drug costs was observed.	HIGH RISK OF BIAS
				6	-	2,131 (1,273)	60	-	2,429 (1,697)	60					
Odeh et al. 2019	Quasiexperimental	Cost-benefit	$	1	37.03	-	211	-	-	-	-	-		Cost benefit analysis indicated that for every pound spent on the service there will be an expected saving of £51.19 at the 30-day interval and £38.08 at the 90-day interval using the PP approach. Likewise, there will be an expected saving of £29.62 at the 30-day interval and £23.58 at 90-day interval using the ITT approach for every pound spent on service delivery. Savings resulted from reduced readmission rates and subsequent expenses.	HIGH RISK OF BIAS
				3	29.48	-	211	-	-	-					

1. The most important components for all countries were the costs of drugs and hospitalisations.
2. Cost of implementing the intervention
3. Including intervention cost
4. Adjusted for baseline number of DRP
5. Associated to ED visits/ Hospitalisations
6. "Total Part D Drug Costs" includes all expenses related to prescription medications covered under Medicare Part D.
7. Within Intervention group
8. Within copntrol group

Bernsten et al. reported no significant difference in total costs between intervention and control groups at any measured point. Minor cost savings observed in the intervention group were mostly due to reduced drug and hospitalization costs, but overall healthcare expenses remained similar between groups. Similarly, Jerant et al. found no significant difference in total healthcare costs between groups at 12 month. Lenander et al.'s pharmacist intervention, estimated at $106 per patient, aimed to reduce drug-related problems but did not report substantial savings in hospital admissions or primary care utilisation, indicating limited cost-effectiveness from a broader perspective. Syafhan et al., however, found significant cost reductions in healthcare resource utilisation within the intervention group over six months.

Van der Heijden et al. observed that costs were higher in the intervention group, with only limited improvement in reducing drug-related problems. This intervention's position on the cost-effectiveness plane indicated increased costs without substantial added benefit. In contrast, Yang et al. found no significant difference in medical costs between intervention and control groups at baseline or three months. Matzke et al. reported significant cost reductions in emergency department visits and hospitalisations, suggesting a positive impact on emergency-related expenses.

Moczygemba et al. found no significant difference in total drug coverage costs, though a slight decrease in Part D drug costs was noted in the intervention group. Odeh et al., through cost-benefit analysis, found that the intervention resulted in significant savings, primarily due to reduced readmission rates.

					INTERVENTION			CONTROL					
**Author/Year**	**Design**	**Outcome**	**Tool**	**Time point (months)**	**n**	**Mean (SD)/ Mean [SE]**	**N**	**n**	**Mean (SD)/ Mean [SE]**	**N**	**P value**	**CONVERSION RATE**	**NOTES**
Bernsten et al. 2001	RCT	Total costs	€	0-6	-	1,506.35 (-)		-	1,643.15		-	1 EUR= 1.15 USD	All costs originally reported in euros (EUR) have been converted to U.S. dollars (USD) using the historical exchange rate applicable for the year 1999, as the prices were updated to January 1999 levels.
				6-12	-	1,498.83 (-)		-	1,815.93		-		
				12-18	-	1,573.59 (-)		-	1,646.37		-		
Bernsten et al. 2001	RCT	Cost-saving	€	0-6	-	-136.80 (-)		-	-		-		
				6-12	-	-317.10 (-)		-	-		-		
				12-18	-	-72.78 (-)		-	-		-		
Syafhan et al. 2021	RCT	Total healthcare resource utilisation cost	£	Baseline	176,042	1,041.7 (1446.7)	169	189,272	1175.6 (1619.3)	161	0.276	1 GBP = 1.30 USD	All amounts originally reported in British pounds (£) have been converted to US dollars (USD) using the historical exchange rate applicable for the year the study was completed (2017).
				6	145,184	859.1 (1235.2)	169	172,615	1072.1 (1635.0)	161			
Van der Heijden et al. 2019	Cluster-RCT	Total healthcare resource utilisation cost	€	12	-	5,450 (1035)	106	-	3796 (437)	110	-	1 EUR = 1.12 USD	Since the study was published in 2019 and we do not have data on the exact completion date of the study, we used the average exchange rate from that year for the conversion.
Yang et al. 2022	Quasiexperimental	Medical costs	Chinese Yuan	Baseline	-	1,642.00 (1244.77)	67	-	1677.54 (1456.71)	69	0.922	1 CNY = 0.15 USD	Since the study was published in 2022 and we do not have data on the exact completion date of the study, we used the average exchange rate from that year for the conversion.
				3	-	1,379.15 (871.31)	67	-	1384.88 (411.23)	69	0.866		
Odeh et al. 2019	Quasiexperimental	Cost-benefit	£	1	29.62	-	211	-	-	-	-	1 GBP = 1.25 USD	All amounts originally reported in British pounds (£) have been converted to U.S. dollars (USD) using the historical exchange rate applicable for the year when the data was collected. Since the screening and recruitment for this study took place from February to June 2016, we utilized the average exchange rate from 2016 for the conversion.
				3	23.58	-	211	-	-	-	-		

Indicates a significant improvement in the outcome measure for the intervention group compared to the control group.

Indicates a non-significant effect or no clear difference in the outcome measure between the intervention and control groups.

Indicates a negative effect, meaning a worsening of the outcome measure in the intervention group compared to the control group.

PATIENTS' BELIEFS AND ATTITUDES TOWARDS MEDICATION					INTERVENTION		CONTROL		Effect Size			
**Authors/ Year**	**Design**	**Outcome**	**Tool**	**Timepoint**	**Mean Score (SD)**	**N**	**Mean Score (SD)**	**N**	**MD/ β**	**CI 95% lower**	**CI 95% upper**	**P value**
Karapinar-Çarkıt et al. 2019	Quasiexperimental	Specific Necessities	BMQ	0	18.68 (4.1)	104	18.23 (3.9)	106	-	-	-	0.43
				1	19.11 (4.2)	62	17.87 (4.0)	66	-	-	-	0.11
		Specific Concerns	BMQ	0	16.29 (4.6)	104	16.47 (4.0)	106	-	-	-	0.77
				1	16.78 (5.1)	62	16.77 (4.1)	66	-	-	-	0.98
Messerli et al. 2016	RCT	Difference Beliefs- Concerns	BMQ	4	10.77 (6.360)	188	11.51 (5.705)	183	-	-	-	0.337
Muth et al. 2018	RCT	Specific Necessities	BMQ	0	22.1 (3.19)	240	22.1 (3.3)	233	-	-	-	-
				6	21.8 (3.5)	230	22.0(2.9)	219	MD: -0.2	-1.0	0.7	0.714
				9	21.9 (3.4)	226	21.6(3.6)	207	MD: 0.1	-0.8	1.0	0.838
		Specific Concerns		0	13.4 (5.2)	238	13.4 (5.2)	229	-	-	-	-
				6	12.8 (4.8)	227	13.1(4.8)	223	MD: -0.2	-0.8	0.5	0.637
				9	12.5 (5.1)	226	12.6 (5.0)	211	MD: 0.0	-0.7	0.6	0.917
Syafhan et al. 2021	RCT	Necessity-concerns differential	BMQ	6	-	-	-	-	-	-	-	-
Yang et al. 2022	Quasiexperimental	Specific Necessities	BMQ	0	19.16 (3.16)	67	18.84 (2.81)	69	-	-	-	0.529
				1.5	20.25 (2.33)	67	19.14 (2.40)	69	-	-	-	-
				3	19.95 (3.30)	67	16.61 (3.48)	69	β: -0.10	-1.13	0.94	0.857
		Specific Concerns	BMQ	0	14.49 (4.85)	67	13.31 (3.43)	69	-	-	-	0.107
				1.5	13.58 (3.95)	67	14.45 (2.96)	69	-	-	-	-
				3	12.89 (3.10)	67	13.98 (3.10)	69	β: 0.71	-0.64	2.06	0.301
Odeh et al. 2019	Quasiexperimental	Necessities	BMQ	0	22.5 (2.6)	83	-	-	-	-	-	-
				3	23.1 (2.0)	83	-	-	MD: 0.6	-0.14	1.18	0.12
		Concerns	BMQ	0	14.2 (4.4)	83	-	-	-	-	-	-
				3	11.0 (3.1)	83	-	-	MD: -3.2	-4.22	-2.27	<0.001
		Necessities-concerns differential	BMQ	0	8.3 (5.3)	83	-	-	-	-	-	-
				3	12.1(3.6)	83	-	-	MD: 3.8	2.60	4.93	<0.001

MD: Mean Difference
β: group effect
*The selected BMQ domains (Specific Necessities, Specific Concerns, and Necessities-Concerns differential) were chosen because they were consistently reported across studies, making them the most comparable and relevant for analysis. Not all studies reported all BMQ domains, so focusing on these allowed for meaningful comparisons and a clearer understanding of patients' beliefs about their medication."

Indicates a significant improvement in the outcome measure for the intervention group compared to the control group.

Indicates a non-significant effect or no clear difference in the outcome measure between the intervention and control groups.

Indicates a negative effect, meaning a worsening of the outcome measure in the intervention group compared to the control group.

Six studies assessed patients' beliefs and attitudes towards medication, all employing the Beliefs about Medicines Questionnaire (BMQ), primarily focused on beliefs/ necessities and medication concerns or the necessity-concern differential. While most studies found no significant differences between intervention and control groups on patients' beliefs and attitudes towards medication, Odeh et al., reported a significant positive effect of the intervention on reducing concerns and improving the necessity-concerns balance.

PATIENTS' KNOWLEDGE ABOUT MEDICINES					INTERVENTION		CONTROL		Effect Size						
**Authors/ Year**	**Design**	**Outcome**	**Tool**	**Timepoint**	**Mean Score (SD)**	**N**	**Mean Score (SD)**	**N**	**MD/ β**	**CI 95% lower limit**	**CI 95% upper limit**	**P value**	**Results**	**Interpretation**	**Risk of Bias**
Bernsten et al. 2001	RCT	Patients' Knowledge on medicines	Self-designed questionnaire [1]	0	65.1 ± 14.51	1290	63.2 ± 17.0	1164	-	-	-	-		There were no significant differences between the control and intervention patients at any assessment point with regard to knowledge of medicines.	HIGH RISK OF BIAS
				6	+3.85±14.61	1024	+2.28±16.10	953	-	-	-	-			
				12	+3.73± 14.75	863	+4.46±16.53	764	-	-	-	-			
				18	+3.19±15.18	704	+3.16±16.19	636	-	-	-	-			
Nazareth et al. 2001	RCT	Knowledge about medicines	The Prescribed Medicine Interview	0	0.68 (0.34)	131	0.60(0.31)	139	MD: 0.08	0.0051	0.154	-		No significant differences were observed at any timepeoint between intervention and control groups, with very small effect sizes (MD ranging from 0.01 to 0.08).	HIGH RISK OF BIAS
				3	0.69(0.33)	86	0.62(0.34)	83	MD: 0.07	-0.032	0.173	-			
				6	0.69 (0.35)	65	0.68 (0.32)	68	MD: 0.01	-0.106	0.126	-			
Taylor et al. 2003	Quasiexperimental	Medication Knowledge	Self-reported/ Medication Knowledege score (%) [2]	0	56.3 (9.7)	33	58.2 (10.4)	36	-	-	-	0.709		Mean medication knowledge scores in the intervention group were 36% higher at 12 months. The control group had a medication knowledge score reduction of 15%.	HIGH RISK OF BIAS
				12	92.6 (3.4)	33	42.9 (12.8)	36	-	-	-	0.000			
Yang et al. 2022	Quasiexperimental	Medication Knowledge	PKMUQ	0	13.64 (2.49)	67	13.91 (3.51)	69	-	-	-	0.603		While the intervention group showed slight improvements at 1.5 months, these differences were not statistically significant at the 3-month mark.	SOME CONCERNS
				1.5	17.57 (3.50)	67	15.27 (3.56)	69	-	-	-	-			
				3	17.62 (3.41)	67	15.16 (3.06)	69	β: -0.10	-1.34	0.94	0.873			

MD: Mean Difference
β: group effect
1. Patients' responses to 4 items within a questionnaire designed for use in the study
2. A knowledge score was determined by dividing the number of medications for which a patient reported the correct name, puspose, dose and frequency by the total number of medications and multiplying by 100.

The four studies evaluating the impact of interventions on patients' knowledge about medicines included Bernsten et al., Nazareth et al., Yang et al., and Taylor et al. These studies used various self-reported tools. Three of them generally found no significant differences between the intervention and control groups at any of the measured time points. Yang et al. observed slight trends toward improvement in medication knowledge, although these differences did not reach statistical significance. In contrast, Taylor et al. reported a significant increase in the medication knowledge score in the intervention group after twelve months, while the control group showed a decline in knowledge.

Indicates a significant improvement in the outcome measure for the intervention group compared to the control group.

Indicates a non-significant effect or no clear difference in the outcome measure between the intervention and control groups.

Indicates a negative effect, meaning a worsening of the outcome measure in the intervention group compared to the control group.

FUNCTIONING/FALLS					INTERVENTION			CONTROL			EFFECT SIZE						
**Authors/ Year**	**Design**	**Outcome**	**Tool**	**Time point**	**Mean score (SD)**	**%**	**N**	**Mean score (SD)**	**%**	**N**	**MD/OR**	**CI 95% lower limit**	**CI 95% upper limit**	**P value**	**Results**	**Interpretation**	**Risk of Bias**
Köberlein-Neu et al. 2016	Cluster-RCT Stepped-wedge design	Mobility	Tinneti	15	21.66 [5.23-6.54]	-	59	22.20 [6.27-7.66]	-	73	MD: 0.46	-0.90	1.82	-		No statistically significant differences between intervention and control groups were found in any of the outcomes.	HIGH RISK OF BIAS
		Activities of Daily	Barthel Index	15	94.99 [93.84-96.14]	-	59	94.83 [93.75- 95.91]	-	73	MD: -0.20	-0.43	0.02	-			
		Instrumental	Lawton and Brody	15	6.40 [6.24-6.55]	-	59	6.64 [6.50-6.78]	-	73	MD: -0.37	-1.13	0.40	-			
Kouladjian O'Donnell et al. 2021	Cluster-RCT	Physical function	Short Physical Performance Battery (SPPB)	Baseline	7.3 (3.2)	-	63	7.6(3.6)	-	96	-	-	-	-		At 3 months, the intervention group showed a small improvement compared to the control group, but this was not statistically significant.	HIGH RISK OF BIAS
				3	7.6 (3.6)	-	63	7.1 (3.2)	-	96	MD: 0.41	-0.60	1.43	-			
		Cognitive function	Mini-Cog test	Baseline	4.2 (1.2)	-	63	3.5 (1.4)	-	96	-	-	-	-		No significant differences were found.	
				3	4.2 (1.1)	-	63	3.7(1.3)	-	96	MD: -0.01	-0.32	0.30	-			
Muth et al. 2018	RCT	Functional status	VES-13	0	2.6 (2.7)	-	223	3.0 (2.9)	-	228	-	-	-	-		The intervention group showed small improvements, but only at 9 months the difference reached statistical significance.	LOW RISK OF BIAS
				6	2.6 (2.8)	-	222	3.0 (2.9)	-	217	MD: 0.1	-0.3	0.5	0.681			
				9	2.8 (2.8)	-	204	2.7 (2.8)	-	199	MD: 0.4	0.0	0.8	0.047			
Kouladjian O'Donnell et al. 2021	Cluster-RCT	Falls	Patient/ Carer self-reported	3	-	12	63	-	10	96	OR: 1.22	0.37	04.02	-		No significant difference was found in the incidence of falls between the intervention and control groups.	HIGH RISK OF BIAS
Messerli et al. 2016	RCT	Falls	Self-reported	7	-	15.9	-	-	17.7	-	-	-	-	0. 638		No significant difference was observed for the incidence of falls during the study period.	HIGH RISK OF BIAS
Poorcheraghi et al. 2023	RCT	Falls	Self reported questionnaire/ Medical records	2	-	7.61		-	22.82					0.007		A significant difference between groups was found, with the intervention group reporting fewer falls.	SOME CONCERNS

MD: Mean Difference; OR: Odds Ratio

The studies assessing mobility, functional status, and falls provided mixed findings. Most studies, including Köberlein-Neu et al., Kouladjian O'Donnell et al., and Messerli et al., showed no significant improvements in mobility, functional status, or fall prevention. Köberlein-Neu et al. used the Tinneti Scale for mobility, the Barthel Index for activities of daily living, and the Lawton and Brody Index for instrumental activities of daily living. Kouladjian O'Donnell et al. measured physical function with the Short Physical Performance Battery (SPPB) and cognitive function with the Mini-Cog test. Messerli et al. used self-reported data for falls. However, Muth et al. demonstrated a small, statistically significant improvement in functional status ,measured with the VES-13, at 9 months, while Poorcheraghi et al. observed a significant reduction in falls, employing a self-reported questionnaire and medical records.

Indicates a significant improvement in the outcome measure for the intervention group compared to the control group.

Indicates a non-significant effect or no clear difference in the outcome measure between the intervention and control groups.

Indicates a negative effect, meaning a worsening of the outcome measure in the intervention group compared to the control group.
