# Supplementary material for: Interventions to enhance in-home taking medication among older adults with multimorbidity/polypharmacy: a systematic review and meta-analysis
Source: Front Public Health. 2026 Jan 28;13:1701622. doi: 10.3389/fpubh.2025.1701622 (PMC12891206; doi:10.3389/fpubh.2025.1701622)
Supplement: Supplementary file 1 [file Data_Sheet_1.zip › Supplementary Table 9. Outcome Measures .pdf]

OUTCOME		METHOD	DESCRIPTION	METRICS
ADHERENCE	Objective measures	**Pill count**	A method to assess medication adherence by counting the number of pills remaining in a medication bottle or blister pack and identifying discrepancies between medicines actually taken and medicines prescribed	Adherence Rate=(Actual number of pills taken / pills prescribed) * 100<br>Patients are defined as compliant with a drug if they take 80-120% of the prescribed daily dose
		**Medication Record Review**	The medication record is reviewed with the aim to assess whether drugs for treatment of a chronic disease first prescribed at discharge are still being used	Discontinuation of drugs newly prescribed=<br>(Number of medications discontinued/ Total number of medications prescribed/)* 100
		**Discrepancies between medicines actually taken and medicines prescribed**	Measures the extent to which patients deviate from their prescribed medication regimen. This includes underuse and overuse.	Percentage of deviating patients = (Number of patients with discrepancies / Total number of patients) * 100. Deviation is assessed using drug, dose, and regimen scores.
		**Refill records**	An indirect method that involves analyzing the dates and frequencies of prescription refills. Metrics such as Primary non-adherence (PMN) and Proportion of Days Covered (PDC) are used to quantify adherence.	PMN= (Number of medications not picked up/ Number of medications prescribed per patient) * 100<br>PDC=(Number of days covered by medication / Number of days in the measurement period) * 100
		**Home Medicine Stocks-Refill Records**	It compares medication refill records and current home inventory. It assesses whether patients are refilling prescriptions on time and have an adequate supply of medication at home.	-
		**Medication Possession Ratio (MPR)**	Measures the proportion of time a patient has access to medication based on the total days' supply dispensed during the observation period.	MPR= (Total days' supply of medication dispensed / Number of days in the observation period) * 100
		**Daily Pill Pick-Up Rate (DPPR)**	Measures the frequency at which patients pick up their prescribed medications within a specified time period.	DPPR = (Total number of pill pick-ups / Number of expected pick-ups) * 100
		**Medication Events Monitoring System (MEMS)**	Using electronic devices (pill bottle caps) that record the date and time when the medication container is opened, tracking adherence based on prescribed frequency and acceptable inter-dose intervals.	Adherence Rate (%) = (Total doses taken within the correct time interval / Total doses scheduled) × 100
	[illegible] Self-report tools and scales	**4-item Morisky Medication Adherence Scale (MMAS-4)**	A self-report questionnaire consisting of four yes/no questions focused on common behaviors related to medication-taking:<br>-"Do you ever forget to take your medicine?"<br>-"Are you careless at times about taking your medicine?"<br>-"When you feel better, do you sometimes stop taking your medicine?"	"Yes" is scored as 1 point, and "no" is scored as 0 points. The items are summed to give a range of scores from 0 to 4. Higher scores indicate better adherence.
		**8-item Morisky Medication Adherence Scale (MMAS-8)**	A self-report questionnaire consisting of eight items providing detailed insights into a patient's medication-taking behavior and potential barriers	Questions 1-7 are scored dichotomously (Yes = 1 point, No = 0 points). Question 8 "how often do you have difficulty remembering to take all your medication?" is scored on a 5-point Likert scale (0 = Never/rarely, 1 = Sometimes, 2 = Often, 3 = Always) 8 = high adherence, 6–7.75 medium adherence, <6 low adherence
		**Malaysian Medication Adherence Scale (MALMAS)**	Scale developed based on the MMAS with 9 items (MMAS-9).	The first item of the MALMAS has five responses: (1) All the time, (2) Often, (3) Sometimes, (4) Rarely and (5) Never. The remaining seven items have a dichotomous response of "Yes" or "No".
		**Medication adherence report scale (MARS)**	A 10-item self-report scale that combines elements of both adherence and beliefs about medication. The items are formulated to reflect common issues that affect adherence, such as forgetfulness, intentional non-adherence, and beliefs about the [illegible]	Each item is scored on a scale (e.g., Likert scale),ranging from 0 (always) to 4 (never), with higher total scores indicating better adherence.

OUTCOME	METHOD	DESCRIPTION	METRICS
Subjective measures:	**Medication Adherence Report Scale-5 (MARS-5)**	It consists of 5 items that assess key aspects of medication adherence, including forgetfulness, carelessness, stopping medication when feeling better or worse, and overall adherence behavior.	5-level response format (1-always, 2-often, 3-sometimes, 4-rarely, and 5-never). Responses are summed for a total score ranging between 5 and 25.
	**Chilean Medication Adherence Questionnaire MAQ (Chilean MAQ)**	Self-report questionnaire used to evaluate medication adherence in Chilean populations. It includes questions about patients' medication-taking behaviors and attitudes toward their treatment.	The questionnaire consists of a series of items related to medication adherence, including frequency, attitudes, and barriers. Responses are typically rated on a Likert scale, with higher scores reflecting better adherence. Specific scoring thresholds for adherence or non-adherence
	**Visual Analogue Scale for Adherence (VASAD)**	The VASAD is a patient-reported tool used to gauge adherence to medication. It involves a visual scale where patients indicate their level of adherence to prescribed medication.	Patients mark a point on a continuous line representing the range from "complete non-adherence" to "perfect adherence."<br>The score is typically expressed in meters (or another unit), where a higher score indicates
	**The prescribed Medicine Interview**	Validated self-report semi-structured interview used to collect data on the patients' knowledge and adherence to prescribed drugs in the previous week and their medication hoarding.	Each item is rated 0 (none) or 1 (highest level) and mean scores are calculated.
SECONDARY OUTCOMES	**Quality of life (EuroQol-5D)**	A standardized instrument for measuring health-related quality of life across five dimensions: mobility, self-care, usual activities, pain/discomfort, and anxiety/depression.	The EQ-5D descriptive system is a preference-based Health Related Quality of Life measure with one question for each of the five dimensions that include mobility, self-care, usual activities, pain/discomfort, and anxiety/depression. The answers given to ED-5D permit to find 243 unique health states or can be converted into EQ-5D index an utility scores anchored at 0 for death and 1 for perfect health. The EQ-5D questionnaire also includes a Visual Analog Scale (VAS), by which respondents can report their perceived health status with a grade ranging from 0 (the worst possible health status) to 100 (the best possible health status).
	**Patients' Beliefs about Medications Questionnaire (BMQ)**	The BMQ consists of two five-item scales assessing patients' beliefs about the necessity of prescribed medication for controlling their disease and their concerns about potential adverse consequences of taking it.	Respondents indicate their degree of agreement with each statement on a five-point Likert scale, ranging from 1 = strongly disagree to 5 = strongly agree. Scores obtained for individual items within both scales are summed. Thus, total scores for the Necessity and Concerns Scales range from 5 to 25. Higher scores indicate stronger beliefs. A necessity–concerns differential is calculated as the difference between the necessity and the concerns scales, with a possible range of −20 to +20. This differential can be thought of as the cost–benefit analysis for each patient, for whom costs (concerns) are weighed against their perceived benefits (necessity beliefs).
	**Patient Knowledge of Medication Usage Questionnaire (PKMUQ)**	This self-report questionnaire evaluates patients' knowledge about their prescribed medications, including their purpose, dosage, timing, and potential side effects.	The cumulative score in the PKMUQ is calculated by summing points across categories (purpose, dosage, timing, side effects).<br>The overall knowledge score is the proportion of correct answers, calculated as:<br>Overall score = (Number of correct responses / Total items) × 100<br>High scores suggest strong medication literacy.
	**Tinneti Test**	This performance-based measure evaluates gait and balance to assess fall risk in older adults. The test consists of two parts: one for balance and another for gait.	Scores range from 0 to 28, with higher scores indicating better balance and gait. A score below 19 is indicative of high fall risk.
	**Barthel Index**	This performance-based measure assesses a person's ability to perform basic Activities of Daily Living (ADLs), such as feeding, bathing, dressing, and mobility.	Scores range from 0 to 100, where higher scores indicate greater independence in daily activities.
	**Lawton and Brody**	This questionnaire-based assessment evaluates an individual's ability to perform instrumental activities of daily living (IADLs), which are essential for independent living. These activities include managing finances, using transportation, shopping,	The scale for women ranges from 0 (indicating low function or dependence) to 8 (indicating high function or independence), while for men, the range is 0 to 5. Higher scores indicate greater functional independence.
	**Short Physical Performance Battery (SPPB)**	The SPPB is a performance-based measure that evaluates physical function by combining three key tests: gait speed, balance, and lower limb strength.	Scores range from 0 to 12, with lower scores indicating poorer physical function and higher risk of disability or frailty.
		[illegible]	

OUTCOME	METHOD	DESCRIPTION	METRICS
	**Mini-Cog test**	In the word recall test, the patient is asked to remember and repeat three words after a brief interval.	The test is scored out of a total of 5 points. Scores ≤ 3 suggest possible cognitive impairment.
	**VES-13**	Self-administered or interview-based questionnaire that identifies elderly individuals at risk of health decline based on age, self-rated health, physical function, and functional disabilities.	Scores ≥3 indicate vulnerability.
