# Supplementary figures and images for: Interventions to enhance in-home taking medication among older adults with multimorbidity/polypharmacy: a systematic review and meta-analysis
Source: Front Public Health. 2026 Jan 28;13:1701622. doi: 10.3389/fpubh.2025.1701622 (PMC12891206; doi:10.3389/fpubh.2025.1701622)

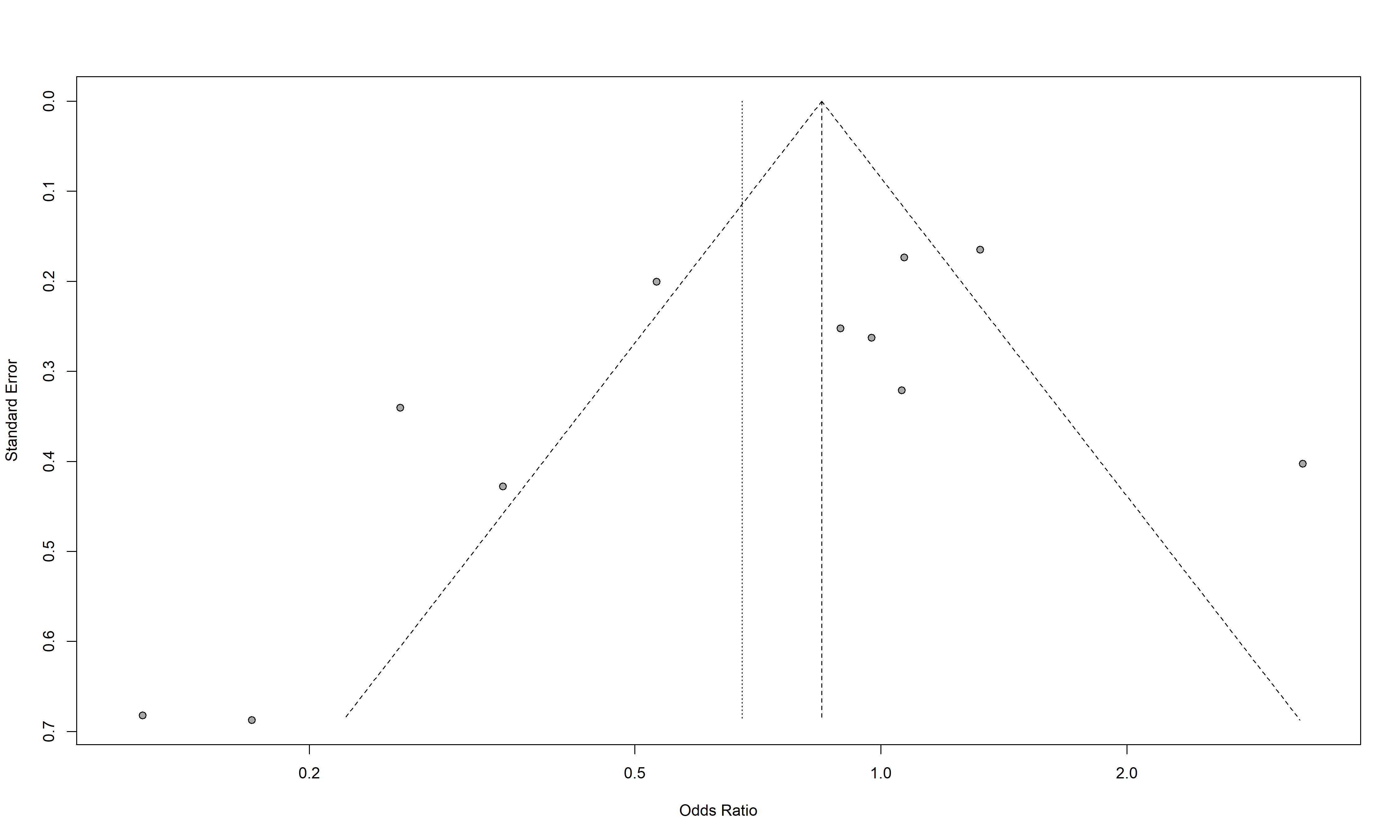

Supplement: Supplementary file 2 [file Presentation_1.zip › Supplementary Figures S1-S6/Supplementary Figure 5. Readmissions funnel plot.tiff]

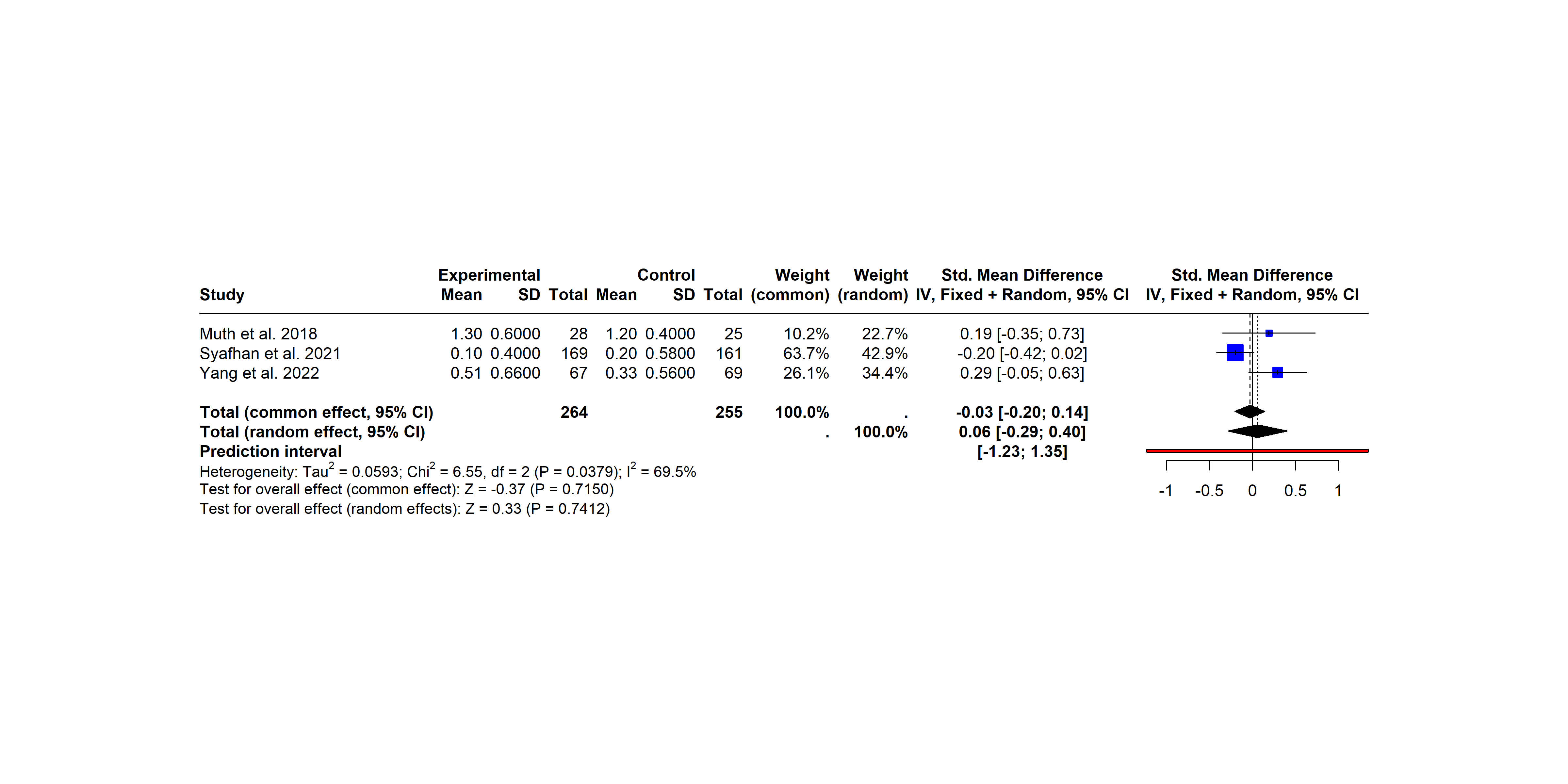

Supplement: Supplementary file 2 [file Presentation_1.zip › Supplementary Figures S1-S6/Supplementary Figure 1.Hospitalisations.tiff]

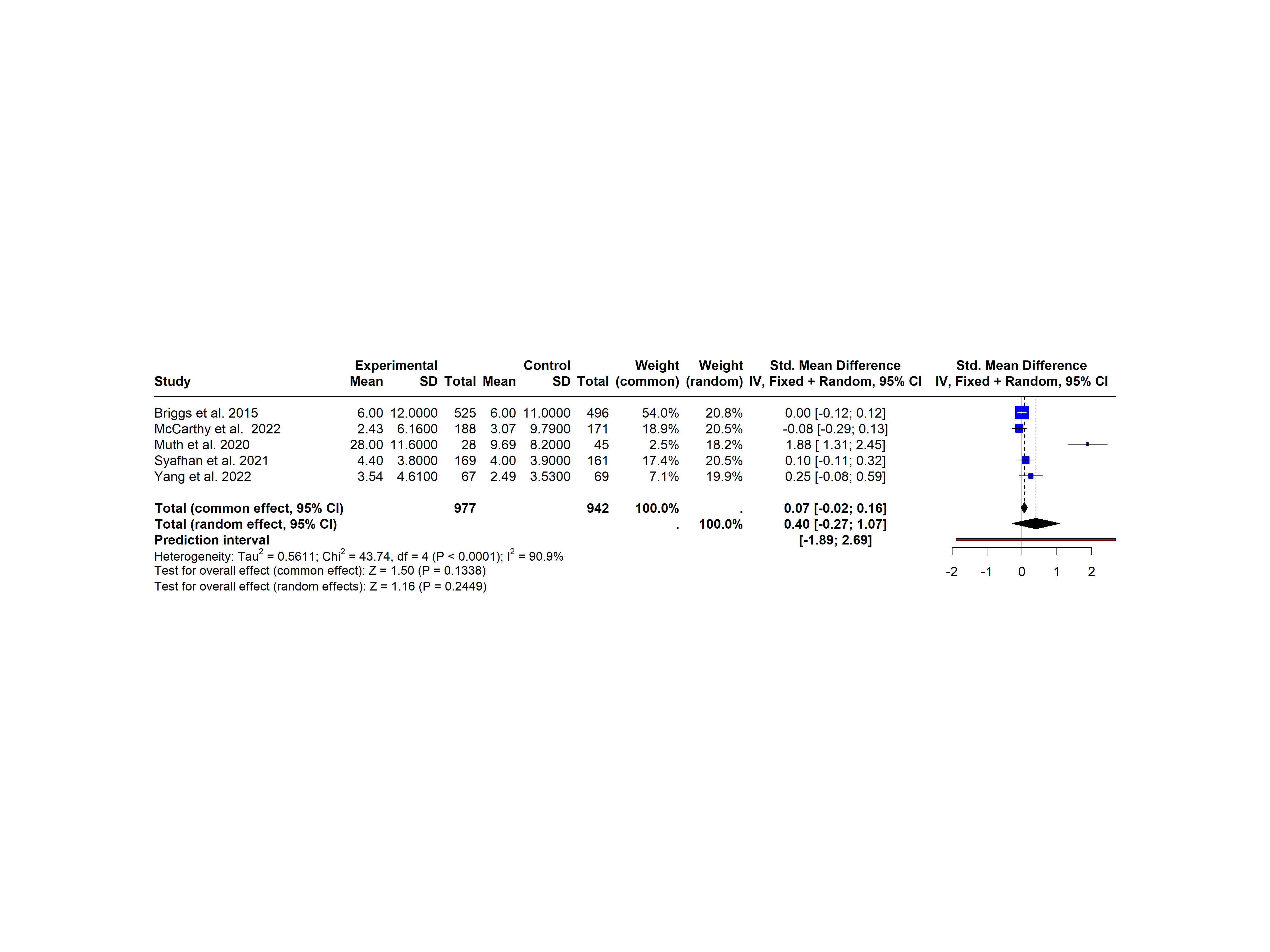

Supplement: Supplementary file 2 [file Presentation_1.zip › Supplementary Figures S1-S6/Supplementary Figure 3.LengthOfstay.tiff]

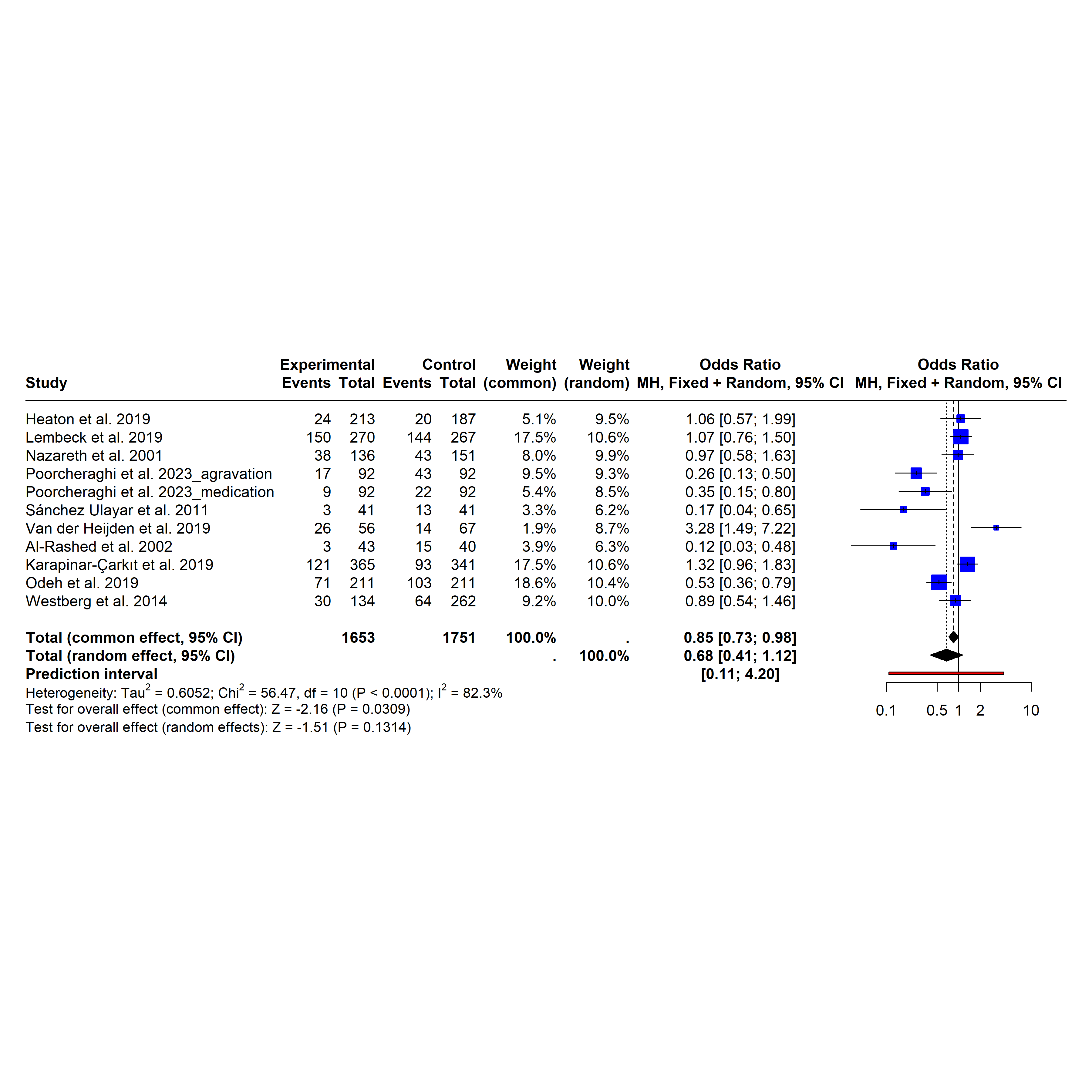

Supplement: Supplementary file 2 [file Presentation_1.zip › Supplementary Figures S1-S6/Supplementary Figure 2.Readmissions overall.tiff]

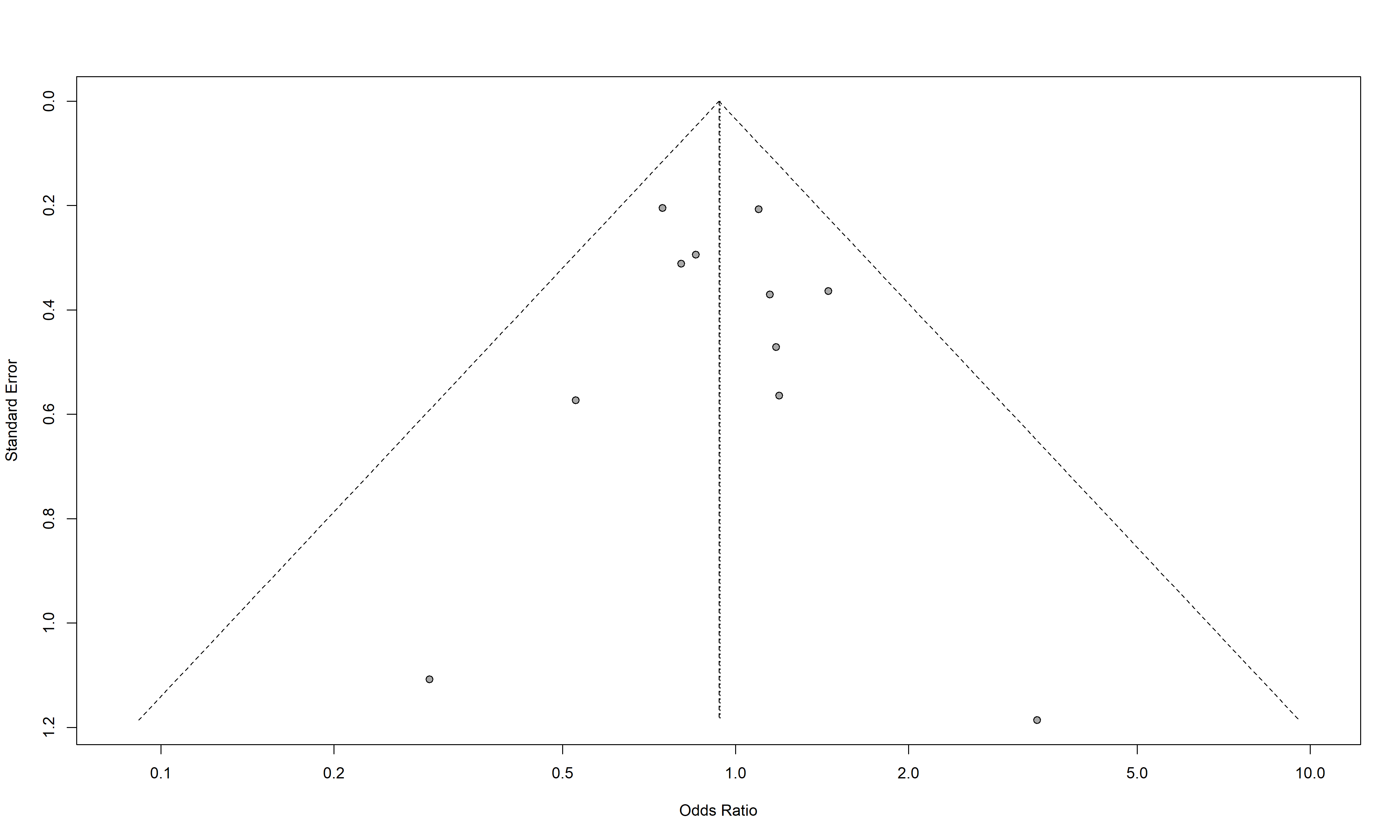

Supplement: Supplementary file 2 [file Presentation_1.zip › Supplementary Figures S1-S6/Supplementary Figure 6. Deaths_funnelplot.tiff]

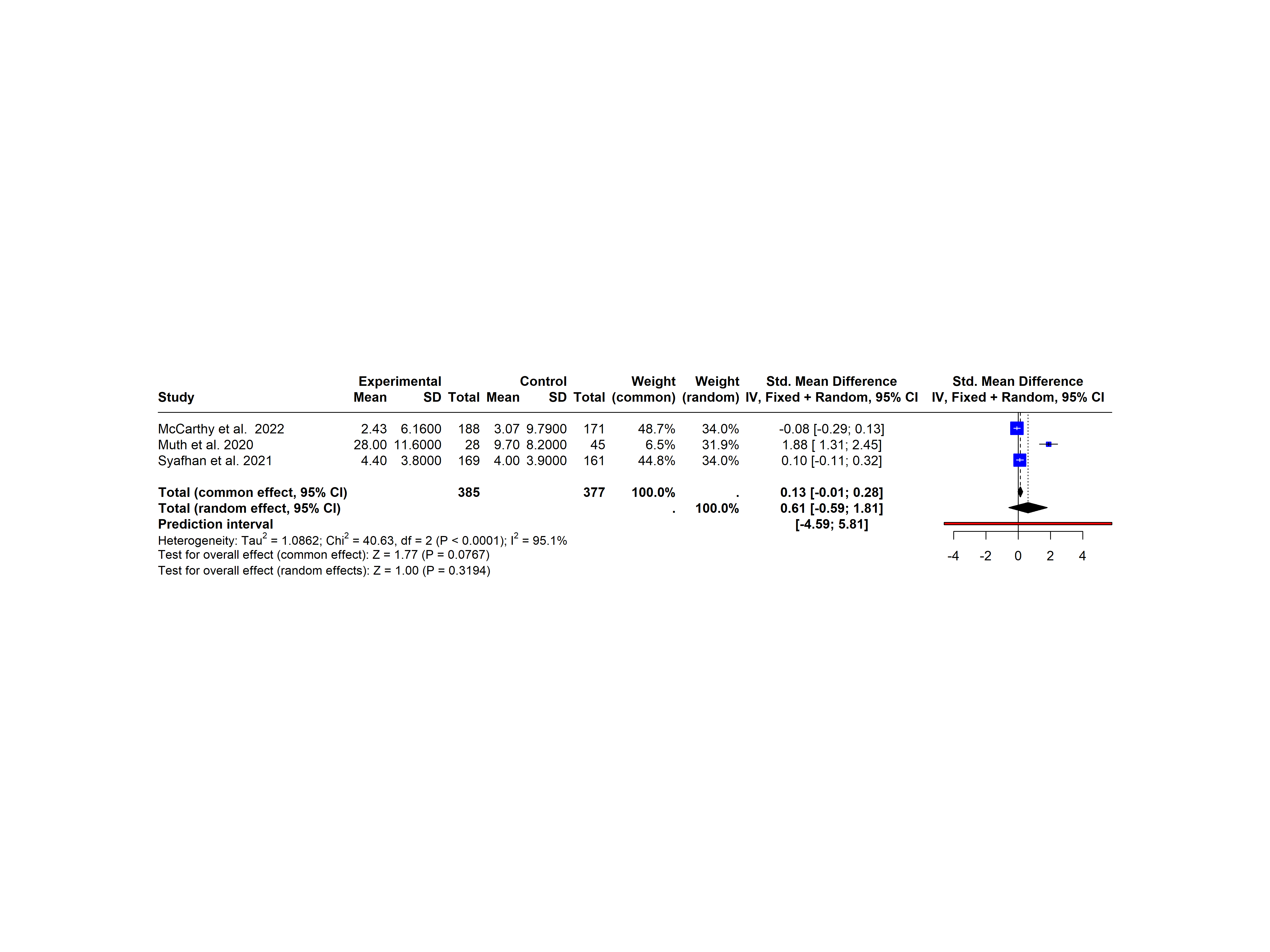

Supplement: Supplementary file 2 [file Presentation_1.zip › Supplementary Figures S1-S6/Supplementary Figure 4.LengthOfstay_sensi.tiff]
